# Hypergeometric decomposition of symmetric K3 quartic pencils

**DOI:** 10.1007/s40687-020-0203-3

**Published:** 2020-03-16

**Authors:** Charles F. Doran, Tyler L. Kelly, Adriana Salerno, Steven Sperber, John Voight, Ursula Whitcher

**Affiliations:** 1 Department of Mathematics, University of Alberta, Edmonton, AB Canada; 2 School of Mathematics, University of Birmingham, Edgbaston, Birmingham B15 2TT UK; 3 Department of Mathematics, Bates College, 3 Andrews Rd., Lewiston, ME 04240 USA; 4 School of Mathematics, University of Minnesota, 206 Church Street SE, Minneapolis, MN 55455 USA; 5 Department of Mathematics, Dartmouth College, 6188 Kemeny Hall, Hanover, NH 03755 USA; 6 Mathematical Reviews, American Mathematical Society, 416 Fourth St, Ann Arbor, MI 48103 USA

## Abstract

We study the hypergeometric functions associated to five one-parameter deformations of Delsarte K3 quartic hypersurfaces in projective space. We compute all of their Picard–Fuchs differential equations; we count points using Gauss sums and rewrite this in terms of finite-field hypergeometric sums; then we match up each differential equation to a factor of the zeta function, and we write this in terms of global *L*-functions. This computation gives a complete, explicit description of the motives for these pencils in terms of hypergeometric motives.

## Introduction

### Motivation

There is a rich history of explicit computation of hypergeometric functions associated to certain pencils of algebraic varieties. Famously, in the 1950s, Igusa [29] studied the Legendre family of elliptic curves and found a spectacular relation between the ${}_2F_1$-hypergeometric Picard–Fuchs differential equation satisfied by the holomorphic period and the trace of Frobenius. More generally, the link between the study of Picard–Fuchs equations and point counts via hypergeometric functions has intrigued many mathematicians. Clemens [11] referred to this phenomenon as “Manin’s unity of mathematics.” Dwork studied the now-eponymous Dwork pencil [20, §6j, p. 73], and Candelas–de la Ossa–Rodríguez-Villegas considered the factorization of the zeta function for the Dwork pencil of Calabi–Yau threefolds in [9, 10], linking physical and mathematical approaches. More recently, given a finite-field hypergeometric function defined over ${\mathbb {Q}}$, Beukers–Cohen–Mellit [3] construct a variety whose trace of Frobenius is equal to the finite-field hypergeometric sum up to certain trivial factors.

### Our context

In this paper, we provide a complete factorization of the zeta function and more generally a factorization of the *L*-series for some pencils of Calabi–Yau varieties, namely families of K3 surfaces. We study certain Delsarte quartic pencils in ${\mathbb {P}}^3$ (also called *invertible pencils*) which arise naturally in the context of mirror symmetry, listed in (1.2.1). Associated to each family, we have a discrete group of symmetries acting symplectically (i.e., fixing the holomorphic form). Our main theorem (Theorem 1.4.1 below) shows that hypergeometric functions are naturally associated to this collection of Delsarte hypersurface pencils in two ways: as Picard–Fuchs differential equations and as traces of Frobenius yielding point counts over finite fields. 
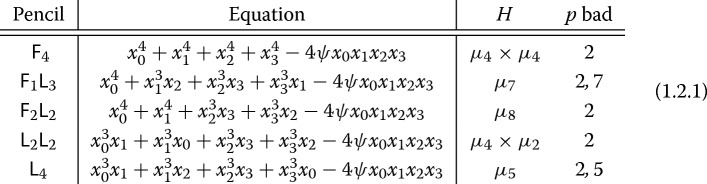
Here, we write $\mu _n$ for the group of roots of unity and *H* is a designated subgroup of symmetries of the family. The labels ${\mathsf {F}}$ and ${\mathsf {L}}$ stand for “Fermat” and “loop,” respectively.

In previous work [18], we showed that these five pencils share a common factor in their zeta functions, a polynomial of degree 3 associated to the hypergeometric Picard–Fuchs differential equation satisfied by the holomorphic form—see also recent work of Kloosterman [36]. Also of note is that the pencils are also related in that one can take a finite group quotient of each family and find that they are then birational to one another [7]. However, these pencils (and their zeta functions) are *not* the same! In this article, we investigate the remaining factors explicitly (again recovering the common factor). In fact, we show that each pencil is associated with a distinct and beautiful collection of auxiliary hypergeometric functions.

### Notation

We use the symbol $\diamond \in {\mathcal {F}}=\{{\mathsf {F}}_4,{\mathsf {F}}_2{\mathsf {L}}_2,{\mathsf {F}}_1{\mathsf {L}}_3,{\mathsf {L}}_2{\mathsf {L}}_2,{\mathsf {L}}_4\}$ to signify one of the five K3 pencils in (1.2.1). Let $\psi \in {\mathbb {Q}}{\smallsetminus } \{0,1\}$. Let $S=S(\diamond ,\psi )$ be the set of bad primes in (1.2.1) together with the primes dividing the numerator or denominator of either $\psi ^4$ or $\psi ^4-1$. Then for $p \not \in S$, the K3 surface $X_{\diamond ,\psi }$ has good reduction at *p*, and for $q=p^r$ we let

1.3.1 $$\begin{aligned} P_{\diamond ,\psi ,q}\left( T\right) :=\det \left( 1 - {{\,\mathrm{Frob}\,}}_p^r T \,|\, H_{\acute{\hbox {e}}\text {t},\text {prim}}^2\left( X_{\diamond ,\psi },{\mathbb {Q}}_\ell \right) \right) \in 1+T{\mathbb {Z}}[T] \end{aligned}$$

be the characteristic polynomial of the *q*-power Frobenius acting on primitive second-degree étale cohomology for $\ell \ne p$, which is independent of $\ell$. (Recall that the primitive cohomology of a hypersurface in ${\mathbb {P}}^n$ is orthogonal to the hyperplane class.) Accordingly, the zeta function of $X_{\diamond ,\psi }$ over ${\mathbb {F}}_q$ is

1.3.2 $$\begin{aligned} Z_q(X_{\diamond ,\psi },T) = \frac{1}{(1-T)(1-qT)P_{\diamond ,\psi ,q}(T)(1-q^2 T)}. \end{aligned}$$

The Hodge numbers of $X_{\diamond ,\psi }$ imply that the polynomial $P_{\diamond ,\psi ,q}(T)$ has degree 21. Packaging these together, we define the (incomplete) *L*-series

1.3.3 $$\begin{aligned} L_S(X_{\diamond ,\psi },s) :=\prod _{p \not \in S} P_{\diamond ,\psi ,p}(p^{-s})^{-1} \end{aligned}$$

convergent for $s \in {\mathbb {C}}$ in a right half-plane.

Our main theorem explicitly identifies the Dirichlet series $L_S(X_{\diamond ,\psi },s)$ as a product of hypergeometric *L*-series. To state this precisely, we now introduce a bit more notation. Let $\varvec{\alpha }=\{\alpha _1,\dots ,\alpha _d\}$ and $\varvec{\beta }=\{\beta _1,\dots ,\beta _d\}$ be multisets with $\alpha _i,\beta _i \in {\mathbb {Q}}_{\ge 0}$ that modulo ${\mathbb {Z}}$ are disjoint. We associate a field of definition $K_{\varvec{\alpha },\varvec{\beta }}$ to $\varvec{\alpha },\varvec{\beta }$, which is an explicitly given finite abelian extension of ${\mathbb {Q}}$. For certain prime powers *q* and $t \in {\mathbb {F}}_q$, there is a *finite-field hypergeometric sum*$H_q(\varvec{\alpha };\varvec{\beta }\,|\,t) \in K_{\varvec{\alpha },\varvec{\beta }}$ defined by Katz [31] as a finite-field analogue of the complex hypergeometric function, normalized by McCarthy [39], extended by Beukers–Cohen–Mellit [3], and pursued by many authors: See section 3.1 for the definition and further discussion, and Sect. 3.2 for an extension of this definition. We package together the exponential generating series associated to these hypergeometric sums into an *L*-series $L_S( H(\varvec{\alpha };\varvec{\beta }\,|\,t), s)$: see Sect. 4.1 for further notation.

### Results

Our main theorem is as follows.

#### Main Theorem 1.4.1

The following equalities hold with $t=\psi ^{-4}$ and $S=S(\diamond ,\psi )$.

- For the Dwork pencil ${\mathsf {F}}_4$,
  $$\begin{aligned} L_S\left( X_{{\mathsf {F}}_4,\psi }, s\right)&= L_S\left( H\left( \tfrac{1}{4}, \tfrac{1}{2}, \tfrac{3}{4}; 0, 0, 0\,|\, t\right) , s\right) \\&\qquad \cdot L_S\left( H\left( \tfrac{1}{4}, \tfrac{3}{4}; 0, \tfrac{1}{2} \,|\, t\right) , s-1, \phi _{-1}\right) ^3 \\&\qquad \cdot L_S\left( H\left( \tfrac{1}{2}; 0 \,|\, t\right) , {\mathbb {Q}}\left( \sqrt{-1}\right) , s-1, \phi _{\sqrt{-1}}\right) ^6, \end{aligned}$$
  where
  1.4.2 $$\begin{aligned} \begin{aligned} \phi _{-1}(p)&:=\biggl (\displaystyle {\frac{-1}{p}}\biggr ) = (-1)^{(p-1)/2}&\text { is associated to } {\mathbb {Q}}(\sqrt{-1}) \,|\, {\mathbb {Q}}, \text { and} \\ \phi _{\sqrt{-1}}({\mathfrak {p}})&:=\biggl (\displaystyle {\frac{\sqrt{-1}}{{\mathfrak {p}}}}\biggr )=(-1)^{({{\,\mathrm{Nm}\,}}({\mathfrak {p}})-1)/4}&\text { is associated to } {\mathbb {Q}}(\zeta _8)\,|\,{\mathbb {Q}}(\sqrt{-1}). \end{aligned} \end{aligned}$$
- For the Klein–Mukai pencil ${\mathsf {F}}_1 {\mathsf {L}}_3$,
  $$\begin{aligned} L_S\left( X_{{\mathsf {F}}_1{\mathsf {L}}_3,\psi }, s\right)&= L_S\left( H\left( \tfrac{1}{4}, \tfrac{1}{2}, \tfrac{3}{4}; 0, 0, 0\,|\, t\right) , s\right) \\&\qquad \cdot L_S\left( H\left( \tfrac{1}{14}, \tfrac{9}{14}, \tfrac{11}{14}; 0, \tfrac{1}{4}, \tfrac{3}{4} \,|\, t^{-1}\right) , {\mathbb {Q}}\left( \zeta _7\right) , s-1\right) , \end{aligned}$$
  where
  $$\begin{aligned} L_S\left( H\left( \tfrac{1}{14}, \tfrac{9}{14}, \tfrac{11}{14}; 0, \tfrac{1}{4}, \tfrac{3}{4} \,|\, t^{-1}\right) , s\right) = L_S\left( H\left( \tfrac{3}{14}, \tfrac{5}{14}, \tfrac{13}{14}; 0, \tfrac{1}{4}, \tfrac{3}{4} \,|\, t^{-1}\right) , s\right) \end{aligned}$$
  are defined over $K={\mathbb {Q}}(\sqrt{-7})$.
- For the pencil ${\mathsf {F}}_2 {\mathsf {L}}_2$,
  $$\begin{aligned} L_S\left( X_{{\mathsf {F}}_2{\mathsf {L}}_2,\psi }, s\right)&= L_S\left( H\left( \tfrac{1}{4}, \tfrac{1}{2}, \tfrac{3}{4}; 0, 0, 0\,|\, t\right) , s\right) \\&\quad \cdot L_S\left( {\mathbb {Q}}\left( \zeta _8\right) \,|\,{\mathbb {Q}},s-1\right) ^2 L_S\left( H\left( \tfrac{1}{4}, \tfrac{3}{4}; 0, \tfrac{1}{2} \,|\, t\right) , s-1, \phi _{-1}\right) \\&\quad \cdot L_S\left( H\left( \tfrac{1}{2};0 \,|\, t\right) , {\mathbb {Q}}\left( \sqrt{-1}\right) , s-1, \phi _{\sqrt{-1}}\right) \\&\quad \cdot L_S\left( H\left( \tfrac{1}{8}, \tfrac{5}{8}; 0, \tfrac{1}{4} \,|\, t^{-1}\right) , {\mathbb {Q}}\left( \zeta _8\right) , s-1, \phi _{\sqrt{2}}\right) , \end{aligned}$$
  where
  $$\begin{aligned} L_S\left( H\left( \tfrac{1}{8}, \tfrac{5}{8}; 0, \tfrac{1}{4} \,|\, t^{-1}\right) , s\right) = L_S\left( H\left( \tfrac{3}{8}, \tfrac{7}{8}; 0, \tfrac{3}{4} \,|\, t^{-1}\right) , s\right) \end{aligned}$$
  are defined over $K={\mathbb {Q}}(\sqrt{-1})$,
  1.4.3 $$\begin{aligned} \begin{aligned} \phi _{\sqrt{2}}\left( {\mathfrak {p}}\right)&:=\biggl (\displaystyle {\frac{\sqrt{2}}{{\mathfrak {p}}}}\biggr ) \equiv 2^{\left( {{\,\mathrm{Nm}\,}}\left( {\mathfrak {p}}\right) -1\right) /4} \pmod {{\mathfrak {p}}}&\text { is associated to } {\mathbb {Q}}\left( \zeta _8,\root 4 \of {2}\right) \,|\, {\mathbb {Q}}\left( \zeta _8\right) , \end{aligned} \end{aligned}$$
  and $L({\mathbb {Q}}(\zeta _8)\,|\,{\mathbb {Q}},s) :=\zeta _{{\mathbb {Q}}(\zeta _8)}(s)/\zeta _{\mathbb {Q}}(s)$ is the ratio of the Dedekind zeta function of ${\mathbb {Q}}(\zeta _8)$ and the Riemann zeta function.
- For the pencil ${\mathsf {L}}_2 {\mathsf {L}}_2$,
  $$\begin{aligned} L_S\left( X_{{\mathsf {L}}_2{\mathsf {L}}_2,\psi }, s\right)&= L_S\left( H\left( \tfrac{1}{4}, \tfrac{1}{2}, \tfrac{3}{4}; 0, 0, 0\,|\, t\right) , s\right) \\&\quad \cdot \zeta _{{\mathbb {Q}}\left( \sqrt{-1}\right) }\left( s-1\right) ^4 L_S\left( H\left( \tfrac{1}{4}, \tfrac{3}{4}; 0, \tfrac{1}{2} \,|\, t\right) , s-1, \phi _{-1}\right) \\&\quad \cdot L_S\left( H\left( \tfrac{1}{8}, \tfrac{3}{8}, \tfrac{5}{8}, \tfrac{7}{8}; 0, \tfrac{1}{4}, \tfrac{1}{2}, \tfrac{3}{4}\,|\, t\right) , {\mathbb {Q}}\left( \sqrt{-1}\right) , s-1, \phi _{\sqrt{-1}}\phi _{\psi }\right) , \end{aligned}$$
  where
  1.4.4 $$\begin{aligned} \begin{aligned} \phi _{\psi }(p)&:=\biggl (\displaystyle {\frac{\psi }{p}}\biggr )&\text { is associated to } {\mathbb {Q}}(\sqrt{\psi })\,|\,{\mathbb {Q}}. \end{aligned} \end{aligned}$$
- For the pencil ${\mathsf {L}}_4$,
  $$\begin{aligned} L_S\left( X_{{\mathsf {L}}_4,\psi }, s\right)&= L_S\left( H\left( \tfrac{1}{4}, \tfrac{1}{2}, \tfrac{3}{4}; 0, 0, 0\,|\, t\right) , s\right) \\&\quad \cdot \zeta \left( s-1\right) ^2 L_S\left( H\left( \tfrac{1}{5}, \tfrac{2}{5}, \tfrac{3}{5}, \tfrac{4}{5}; 0, \tfrac{1}{4}, \tfrac{1}{2}, \tfrac{3}{4} \,|\, t^{-1}\right) , {\mathbb {Q}}\left( \zeta _5\right) , s-1\right) . \end{aligned}$$

We summarize Theorem 1.4.1 for each of our five pencils in (1.4.5): We list the degree of the *L*-factor, the hypergeometric parameters, and the base field indicating when it arises from base change. A Dedekind (or Riemann) zeta function factor has factors denoted by-. 
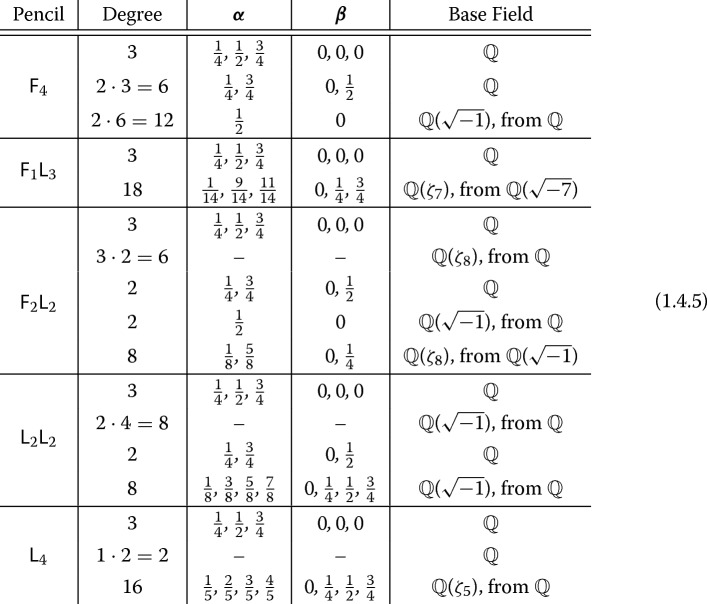


We extensively checked the equality of Euler factors in Main Theorem 1.4.1 in numerical cases (for many primes and values of the parameter $\psi$): For K3 surfaces, we used code written by Costa [14], and for the finite-field hypergeometric sums we used code in Pari/GP and Magma [8], the latter available for download [49]. See also Example 4.8.3.

Additionally, each pencil has the common factor $L_S(H(\frac{1}{4},\frac{1}{2},\frac{3}{4};0,0,0\,|\,t),s)$, giving another proof of a result in previous work [18]: We have a factorization over ${\mathbb {Q}}[T]$

1.4.6 $$\begin{aligned} P_{\diamond ,\psi ,p}(T) = Q_{\diamond ,\psi ,p}(T)R_{\psi ,p}(T) \end{aligned}$$

with $R_{\psi ,p}(T)$ of degree 3 independent of $\diamond \in {\mathcal {F}}$. The common factor $R_{\psi ,p}(T)$ is given by the action of Frobenius on the transcendental part in cohomology, and the associated completed *L*-function $L(H(\tfrac{1}{4},\tfrac{1}{2},\tfrac{3}{4};0,0,0 \,|\, t),s)$ is automorphic by Elkies–Schütt [21] (or see our summary [18, §5.2]): It arises from a family of classical modular forms on ${{\,\mathrm{GL}\,}}_2$ over ${\mathbb {Q}}$, and in particular, it has analytic continuation and functional equation. See also recent work of Naskręcki [43].

The remaining factors in each pencil in Main Theorem 1.4.1 yield a factorization of $Q_{\diamond ,\psi ,p}(T)$, corresponding to the algebraic part in cohomology (i.e., the Galois action on the Néron–Severi group). In particular, the polynomial $Q_{\diamond ,\psi ,p}(T)$ has reciprocal roots of the form *p* times a root of unity. The associated hypergeometric functions are *algebraic* by the criterion of Beukers–Heckman [4], and the associated *L*-functions can be explicitly identified as Artin *L*-functions: See Sect. 4.7. The algebraic *L*-series can also be explicitly computed when they are defined over ${\mathbb {Q}}$ [13, 44]. For example, if we look at the Artin *L*-series associated to the Dwork pencil ${\mathsf {F}}_4$, Cohen has given the following *L*-series relations (see Proposition 4.7.2):

1.4.7 $$\begin{aligned} \begin{aligned} L_S\left( H\left( \tfrac{1}{4}, \tfrac{3}{4}; 0, \tfrac{1}{2}\,|\,\psi ^{-4}\right) , s, \phi _{-1}\right)&= L_S\left( s, \phi _{1-\psi ^2}\right) L_S\left( s,\phi _{-1-\psi ^2}\right) \\ L_S(H\left( \tfrac{1}{2};0\,|\,\psi ^{-4}, {\mathbb {Q}}\left( \sqrt{-1}\right) ,s, \phi _{\sqrt{-1}}\right)&= L_S\left( s, \phi _{2\left( 1-\psi ^4\right) }\right) L_S\left( s,\phi _{-2\left( 1-\psi ^4\right) }\right) . \end{aligned} \end{aligned}$$

In particular, it follows that the minimal field of definition of the Néron–Severi group of $X_{{\mathsf {F}}_4,\psi }$ is ${\mathbb {Q}}(\zeta _8,\sqrt{1-\psi ^2},\sqrt{1+\psi ^2})$. The expressions (1.4.7), combined with Main Theorem 1.4.1(a), resolve a conjecture of Duan [19]. (For geometric constructions of the Néron–Severi group of $X_{{\mathsf {F}}_4,\psi }$, see Bini–Garbagnati [6] and Kloosterman [36]; the latter also provides an approach to explicitly construct generators of the Néron–Severi group for four of the five families studied here, with the stubborn case ${\mathsf {F}}_1 {\mathsf {L}}_3$ still unresolved.) Our theorem yields an explicit factorization of $Q_{\diamond ,\psi ,q}(T)$ for the Dwork pencil over ${\mathbb {F}}_q$ for any odd *q* (see Corollary 4.7.4). As a final application, Corollary 4.8.1 shows how the algebraic hypergeometric functions imply the existence of a factorization of $Q_{\diamond ,\psi ,p}(T)$ over ${\mathbb {Q}}[T]$ depending only on *q* for all families.

#### Remark 1.4.8

Our main theorem can be rephrased as saying that the motive associated to primitive middle-dimensional cohomology for each pencil of K3 surfaces decomposes into the direct sum of hypergeometric motives as constructed by Katz [31]. These motives then govern both the arithmetic and geometric features of these highly symmetric pencils. Absent a reference, we do not invoke the theory of hypergeometric motives in our proof.

### Contribution and relation to previous work

Our main result gives a complete decomposition of the cohomology for the five K3 pencils into hypergeometric factors. We provide formulas for each pencil and for all prime powers *q*, giving an understanding of the pencil over ${\mathbb {Q}}$. Addressing these subtleties, and consequently giving a result for the global *L*-function, is unique to our treatment. Our point of view is computational and explicit; we expect that our methods will generalize and perhaps provide an algorithmic approach to the hypergeometric decomposition for other pencils.

As mentioned above, the study of the hypergeometricity of periods and point counts enjoys a long-standing tradition. Using his *p*-adic cohomology theory, Dwork [20, §6j, p. 73] showed for the family ${\mathsf {F}}_4$ that middle-dimensional cohomology decomposes into pieces according to three types of differential equations. Kadir in her Ph.D. thesis [30, §6.1] recorded a factorization of the zeta function for ${\mathsf {F}}_4$, a computation due to de la Ossa. Building on the work of Koblitz [37], Salerno [45, §4.2.1–4.2.2] used Gauss sums in her study of the Dwork pencil in arbitrary dimension; under certain restrictions on *q*, she gave a formula for the number of points modulo *p* in terms of truncated hypergeometric functions as defined by Katz [31] as well as an explicit formula [46, §5.4] for the point count for the family ${\mathsf {F}}_4$. Goodson [26, Theorems 1.1–1.3] looked again at ${\mathsf {F}}_4$ and proved a similar formula for the point counts over ${\mathbb {F}}_q$ for all primes $q=p$ and prime powers $q \equiv 1 ({\mathrm{mod}} {4})$. In [23], Fuselier et al. define an alternate finite field hypergeometric function (which differs from those by Katz, McCarthy, and Beukers–Cohen–Mellit) that makes it possible to prove identities that are analogous to well-known ones for classical hypergeometric functions. They then use these formulas to compute the number of points of certain hypergeometric varieties.

Several authors have also studied the role of hypergeometric functions over finite fields for the Dwork pencil in arbitrary dimension, which for K3 surfaces is the family ${\mathsf {F}}_4$ given in Main Theorem 1.4.1. McCarthy [41] extended the definition of *p*-adic hypergeometric functions to provide a formula for the number of ${\mathbb {F}}_p$ points on the Dwork pencil in arbitrary dimension for all odd primes *p*, extending his results [38] for the quintic threefold pencil. Goodson [25, Theorem 1.2] then used McCarthy’s formalism to rewrite the formula for the point count for the Dwork family in arbitrary dimension in terms of hypergeometric functions when $(n+1) \mid (q-1)$ and *n* is even. See also Katz [32], who took another look at the Dwork family.

Miyatani [42, Theorem 3.2.1] has given a general formula that applies to each of the five families, but with hypotheses on the congruence class of *q*. It is not clear that one can derive our decomposition from the theorem of Miyatani.

A different line of research has been used to describe the factorization structure of the zeta function for pencils of K3 surfaces or Calabi–Yau varieties that can recover part of Main Theorem 1.4.1. Kloosterman [34, 35] has shown that one can use a group action to describe the distinct factors of the zeta function for any one-parameter monomial deformation of a diagonal hypersurface in weighted projective space. He then applied this approach [36] to study the K3 pencils above and generalize our work on the common factor. His approach is different from both that work and the present one: He uses the Shioda map [47] to provide a dominant rational map from a monomial deformation of a diagonal (Fermat) hypersurface to the K3 pencils. The Shioda map has been used in the past [7] to recover the result of Doran–Greene–Judes matching Picard–Fuchs equations for the quintic threefold examples, and it was generalized to hypersurfaces of fake weighted projective spaces and BHK mirrors [5, 33]. Kloosterman also provides some information about the other factors in some cases.

### Proof strategy and plan of paper

The proof of Main Theorem 1.4.1 is an involved calculation. Roughly speaking, we use the action of the group of symmetries to calculate hypergeometric periods and then use this decomposition to guide an explicit decomposition of the point count into finite-field hypergeometric sums.

Our proof follows three steps. First, in Sect. 2, we find all Picard–Fuchs equations via the diagrammatic method developed by Candelas–de la Ossa–Rodríguez-Villegas [9, 10] and Doran–Greene–Judes [17] for the Dwork pencil of quintic threefolds. For each of our five families, we give the Picard–Fuchs equations in a convenient hypergeometric form.

Second, in Sect. 3, we carry out the core calculations by counting points over ${\mathbb {F}}_q$ for the corresponding pencils using Gauss sums. This technique begins with the original method of Weil [50], extended by Delsarte and Furtado Gomida, and fully explained by Koblitz [37]. We then take these formulas and, using the hypergeometric equations found in Sect. 2 and careful manipulation, link these counts to finite-field hypergeometric functions. The equations computed in Sect. 2 do not enter *directly* into the proof of the theorem, but they give an answer that can then be verified by some comparatively straightforward manipulations. These calculations confirm the match predicted by Manin’s “unity” (see [11]).

Finally, in Sect. 4, we use the point counts from Sect. 3 to explicitly describe the *L*-series for each pencil, and prove Main Theorem 1.4.1. We conclude by relating the *L*-series to factors of the zeta function for each pencil.

## Picard–Fuchs equations

In this section, we compute the Picard–Fuchs equations associated to all primitive cohomology for our five symmetric pencils of K3 surfaces defined in (1.2.1). Since we are working with pencils in projective space, we are able to represent 21 of the $h^2(X_\psi ) = 22$ dimensions of the second-degree cohomology as elements in the Jacobian ring, that is, the primitive cohomology of degree two for the quartic pencils in ${\mathbb {P}}^3$. We employ a more efficient version of the Griffiths–Dwork technique which exploits discrete symmetries. This method was previously used by Candelas–de la Ossa–Rodríguez-Villegas [9, 10] and Doran-Greene–Judes [17]. Gährs [24] used a similar combinatorial technique to study Picard–Fuchs equations for holomorphic forms on invertible pencils. After explaining the Griffiths–Dwork technique for symmetric pencils in projective space, we carry out the computation for two examples in thorough detail, and then state the results of the computation for three others.

### Setup

We briefly review the computational technique of Griffiths–Dwork [9, 10, 17], and we begin with the setup in some generality.

Let $X \subset {\mathbb {P}}^n$ be a smooth projective hypersurface over ${\mathbb {C}}$ defined by the vanishing of $F(x_0, \ldots , x_n) \in {\mathbb {C}}[x_0,\dots ,x_n]$ homogeneous of degree *d*. Let $A^i(X)$ be the space of rational *i*-forms on ${\mathbb {P}}^n$ with polar locus contained in *X*, or equivalently regular *i*-forms on ${\mathbb {P}}^n \setminus X$. By Griffiths [28, Corollary 2.1], any $\varphi \in A^n(X)$ can be written as

2.1.1 $$\begin{aligned} \varphi = \frac{Q(x_0, \ldots , x_n)}{F(x_0,\ldots , x_n)^k}\Omega _0, \end{aligned}$$

where $k \ge 0$ and $Q \in {\mathbb {C}}[x_0,\dots ,x_n]$ is homogeneous of degree $\deg Q = k\deg F - (n+1)$ and

2.1.2 $$\begin{aligned} \Omega _0 :=\sum _{i=0}^n (-1)^i x_i \,\mathrm {d}{x_0}\wedge \ldots \wedge \mathrm {d}{x_{i-1}} \wedge \mathrm {d}{x_{i+1}} \wedge \ldots \wedge \mathrm {d}{x_n}. \end{aligned}$$

We define the de Rham cohomology groups

2.1.3 $$\begin{aligned} {\mathcal {H}}^i(X) :=\frac{A^i(X)}{\mathrm {d}{A^{i-1}}(X)}. \end{aligned}$$

There is a *residue map*

$$\begin{aligned} {{\,\mathrm{Res}\,}}:{\mathcal {H}}^{n}(X) \rightarrow H^{n-1}(X,{\mathbb {C}}) \end{aligned}$$

made famous by seminal work of Griffiths [28], mapping into the middle-dimensional Betti cohomology of the hypersurface *X*. Given $\varphi \in A^n(X)$, we choose an $(n-1)$-cycle $\gamma$ in *X* and $T(\gamma )$ a circle bundle over $\gamma$ with an embedding into the complement ${\mathbb {P}}^n \setminus X$ that encloses $\gamma$, and define ${{\,\mathrm{Res}\,}}(\varphi )$ to be the $(n-1)$-cocycle such that

2.1.4 $$\begin{aligned} \frac{1}{2\pi \sqrt{-1}} \int _{T(\gamma )} \varphi = \int _{\gamma } {{\,\mathrm{Res}\,}}(\varphi ) \end{aligned}$$

is well-defined for $\varphi \in {\mathcal {H}}^n(X)$. Two circle bundles $T(\gamma )$ with small enough radius are homologous in $H_n({\mathbb {P}}^n \setminus X, {\mathbb {Z}})$, so the class ${{\,\mathrm{Res}\,}}(\varphi ) \in H^{n-1}(X,{\mathbb {C}})$ is well-defined.

There is a filtration on ${\mathcal {H}}^n(X)$ by an upper bound on the order of the pole along *X*:

$$\begin{aligned} {\mathcal {H}}^n_1(X) \subseteq {\mathcal {H}}^n_2(X) \subseteq \ldots \subseteq {\mathcal {H}}^n_{n}(X) = {\mathcal {H}}^n(X). \end{aligned}$$

This filtration on ${\mathcal {H}}^n(X)$ is compatible with the Hodge filtration on $H^{n-1}(X,{\mathbb {C}})$: If we define

$$\begin{aligned} {\mathcal {F}}^k(X) :=H^{n-1,0}(X,{\mathbb {C}}) \oplus \ldots \oplus H^{k, n-k-1}(X, {\mathbb {C}}), \end{aligned}$$

then the residue map restricts to ${{\,\mathrm{Res}\,}}:{\mathcal {H}}^n_k(X) \rightarrow {\mathcal {F}}^{n-k}(X)$.

In certain circumstances, we may be able to reduce the order of the pole [28, Formula 4.5]: We have

2.1.5 $$\begin{aligned} \frac{\Omega _0}{F(x_i)^{k+1}} \sum _{j=0}^n Q_j(x_i) \frac{ \partial F(x_i)}{\partial x_j} = \frac{1}{k} \frac{\Omega _0}{F(x_i)^k} \sum _{j=0}^n \frac{\partial Q_j(x_i)}{\partial x_j} + \omega , \end{aligned}$$

where $\omega$ is an exact rational form. In fact, Eq. (2.1.5) implies that the order of a form $\varphi$ can be lowered (up to an exact form) if and only if the polynomial *Q* is in the Jacobian ideal *J*(*F*), that is, the (homogeneous) ideal generated by all partial derivatives of *F*. So for $k \ge 1$, we have a natural identification

2.1.6 $$\begin{aligned} \frac{{\mathcal {H}}^n_k(X)}{{\mathcal {H}}^n_{k-1}(X)} \xrightarrow {\sim } \left( \frac{{\mathbb {C}}[x_0, \ldots , x_n]}{J(F)}\right) _{k\deg F -(n+1)}, \end{aligned}$$

which by the residue map induces an identification

2.1.7 $$\begin{aligned} \left( \frac{{\mathbb {C}}[x_0, \ldots , x_n]}{J(F)}\right) _{k\deg F -(n+1)} \rightarrow H^{n-k, k-1}(X), \end{aligned}$$

whose image is the primitive cohomology group $H^{n-k,k-1}_{\text {prim}}(X)$, which we know is the cohomology orthogonal to the hyperplane class since *X* is a hypersurface in ${\mathbb {P}}^n$.

#### Example 2.1.8

For *X* a quartic hypersurface in ${\mathbb {P}}^3$, the identification (2.1.7) reads

2.1.9 $$\begin{aligned} \frac{{\mathbb {C}}[x_0, x_1,x_2,x_3]_{4k}}{J(F)} \simeq H^{2-k,k}_{\text {prim}}(X). \end{aligned}$$

In this case, the Hodge numbers are given by $h^{2,0} = 1$, $h^{1,1} = 35 - 4\cdot 4 = 19$, and $h^{0,2} = 165 - 4\cdot 56 + 6\cdot 10 = 1$.

### Griffiths–Dwork technique

Now, suppose that $X_{\psi }$ is a pencil of hypersurfaces in the parameter $\psi$, defined by $F_\psi =0$. Let $\{\gamma _j\}_j$ be a basis for $H_{n-1}(X_{\psi },{\mathbb {C}})$ with cardinality $h_{n-1} :=\dim _{{\mathbb {C}}} H_{n-1}(X_{\psi },{\mathbb {C}})$.

#### Remark 2.2.1

There is a subtle detail about taking a parallel transport using an Ehresmann connection to obtain a (locally) unique horizontal family of homology classes [17, §2.3]. This detail does not affect our computations.

We then choose a basis of (possibly $\psi$-dependent) $(n-1)$-forms $\Omega _{X_\psi , i} \in H^{n-1}(X,{\mathbb {C}})$ so that each of the forms $\Omega _{X_\psi , i} \in H^{n-1}(X,{\mathbb {C}})$ has fixed bidegree (*p*, *q*) which provides a basis for the Hodge decomposition $H^{n-1}(X,{\mathbb {C}}) = \bigoplus _{p+q = n-1} H^{p,q}(X)$ for each fixed $\psi$. We now examine the period integrals

$$\begin{aligned} \int _{\gamma _j} \Omega _{X_\psi , i} \end{aligned}$$

for $$1 \le i,j \le h_{n-1}$$.

We want to understand how these integrals vary with respect to the pencil parameter $\psi$. To do so, we simply differentiate with respect to $\psi$, or equivalently integrate on the complement of $X_\psi$ in ${\mathbb {P}}^n$ as outlined above. Using the residue relation (2.1.4), we rewrite

2.2.2 $$\begin{aligned} \int _{\gamma _{j}} \Omega _{X_\psi , i} = \int _{T(\gamma _{j})} \frac{Q_i}{F_\psi ^{k}} \Omega _0, \end{aligned}$$

for some $Q_i \in {\mathbb {C}}[x_0, \ldots , x_n]_{k\deg F_{\psi } -(n+1)}$ and $k \in {\mathbb {Z}}_{\ge 0}$ (and circle bundle $T(\gamma _{j})$ with sufficiently small radius as above). By viewing $F_\psi$ as a function $F:{\mathbb {C}}\rightarrow {\mathbb {C}}[x_0,\ldots , x_n]$ with parameter $\psi$, we can differentiate $F(\psi )$ with respect to $\psi$ and study how this period integral varies:

2.2.3 $$\begin{aligned} \frac{\mathrm {d}}{\mathrm {d}\psi } \int _{T(\gamma _j)} \frac{Q_i}{F(\psi )^k}\Omega _0 = -k \int _{T(\gamma _j)} \frac{Q_i}{F(\psi )^{k+1}} \frac{\mathrm {d}F}{\mathrm {d}\psi }\Omega _0. \end{aligned}$$

Note that the right-hand side of (2.2.3) gives us a new $(n-1)$-form.

We know that we will find a linear relation if we differentiate $\dim _{\mathbb {C}}H^{n-1}(X_\psi ,{\mathbb {C}})$ times, giving us a single-variable ordinary differential equation called the *Picard–Fuchs equation* for the period $\int _{\gamma _{j}} \Omega _{X_\psi , i}$. In practice, fewer derivatives may be necessary.

For simplicity, we suppose that $F_\psi$ is linear in the variable $\psi$. Then the *Griffiths–Dwork technique* for finding the Picard–Fuchs equation is the following procedure (see [15] or [17] for a more detailed exposition):

1. Differentiate the period *b* times, $1 \le b \le h_{n-1}$. We obtain the equation
  $$\begin{aligned} \left( \frac{\mathrm {d}}{\mathrm {d}\psi }\right) ^b \int _{T(\gamma _j)} \frac{Q_i}{F(\psi )^k}\Omega _0 = \frac{(k+b-1)!}{(k-1)!} \int _{T(\gamma _j)} \frac{Q_i}{F(\psi )^{k+b}}\left( -\displaystyle {\frac{\mathrm {d}F}{\mathrm {d}\psi }}\right) ^b\Omega _0. \end{aligned}$$
2. Write
  2.2.4 $$\begin{aligned} Q_i \left( -\displaystyle {\frac{\mathrm {d}F}{\mathrm {d}\psi }}\right) ^b = \sum _{j=1}^{h_{n-1}} \alpha _j Q_j + J, \end{aligned}$$
  where $\alpha _k \in {\mathbb {C}}(\psi )$ and *J* is in the Jacobian ideal, so we may write $J = \sum _i A_i \displaystyle {\frac{\partial F_\psi }{\partial x_i}}$ with $A_i \in {\mathbb {C}}(\psi )[x_0, \ldots , x_n]$ for all *i*.
3. Use (2.1.5) to reduce the order of the pole of $\displaystyle {\frac{J}{F(\psi )^k}\Omega _0}$. We obtain a new numerator polynomial of lower degree.
4. Repeat steps 2 and 3 for the new numerator polynomials, until the *b*th derivative is expressed in terms of the chosen basis for cohomology.
5. Use linear algebra to find a ${\mathbb {C}}(\psi )$-linear relationship between the derivatives.

While algorithmic and assured to work, this method can be quite tedious to perform. Moreover, the structure of the resulting differential equation may not be readily apparent.

### A diagrammatic Griffiths–Dwork method

In this section, we give a computational technique that uses discrete symmetries of pencils of Calabi–Yau hypersurfaces introduced by Candelas–de la Ossa–Rodríguez-Villegas [9, 10]. To focus on the case at hand, we specialize to the case of quartic surfaces and explain this method so their diagrammatic and effective adaptation of the Griffiths–Dwork technique can be performed for the five pencils that we want to study.

Let $x^{{\mathbf {v}}} :=x_0^{v_0}x_1^{v_1}x_2^{v_2}x_3^{v_3}$, and let $k({\mathbf {v}}) :=\frac{1}{4} \sum _i v_i$; for a monomial arising from (2.1.9), we have $k({\mathbf {v}}) \in {\mathbb {Z}}_{\ge 0}$. Fix a cycle $\gamma$, and consider the periods

2.3.1 $$\begin{aligned} (v_0,v_1,v_2,v_3) :=\int _{T(\gamma )} \frac{x^{{\mathbf {v}}}}{F_\psi ^{k({\mathbf {v}})+1}}\Omega _0 . \end{aligned}$$

Consider the relation

2.3.2 $$\begin{aligned} \partial _i \left( \frac{x_i x^{{\mathbf {v}}}}{F_\psi ^{k({\mathbf {v}})+1}}\right) = \frac{x^{{\mathbf {v}}}}{F_\psi ^{k({\mathbf {v}})+1}}(1+v_i) - (k({\mathbf {v}})+1) \frac{ x^{{\mathbf {v}}}}{F_\psi ^{k({\mathbf {v}})+2}}x_i \partial _i F_\psi . \end{aligned}$$

We can use (2.3.2) in order to simplify the computation of the Picard–Fuchs equation: Integrating over $T(\gamma )$, the left-hand side vanishes, so we can solve for $(v_0,v_1,v_2,v_3)$:

2.3.3 $$\begin{aligned} (1+v_i) (v_0,v_1,v_2,v_3) :=\int _{T(\gamma )} \frac{x^{{\mathbf {v}}}}{F_\psi ^{k({\mathbf {v}})+1}} \Omega _0 = (k({\mathbf {v}})+1)\int _{T(\gamma )} \frac{x^{{\mathbf {v}}}x_i\partial _iF_\psi }{F_\psi ^{k({\mathbf {v}})+2}}\Omega _0. \end{aligned}$$

#### Example 2.3.4

Consider the Dwork pencil ${\mathsf {F}}_4$, the pencil defined by the vanishing of

$$\begin{aligned} F_{\psi } = x_0^4 + x_1^4 + x_2^4 + x_3^4 - 4\psi x_0x_1x_2x_3. \end{aligned}$$

Simplifying the right-hand side of (2.3.3) gives us the relation of periods:

$$\begin{aligned} (1+v_i) (v_0,v_1,v_2,v_3)&= 4(k({\mathbf {v}})+1)\left( (v_0,\ldots , v_i + 4, \ldots , v_3) \right. \\&\quad \left. -\, \psi (v_0+1, v_1+1, v_2+1,v_3+1)\right) \end{aligned}$$

or in a more useful form

2.3.5 $$\begin{aligned} (v_0,\ldots , v_i + 4, \ldots , v_3) = \frac{1+v_i}{4(k({\mathbf {v}})+1)} (v_0,v_1,v_2,v_3) + \psi (v_0+1, v_1+1, v_2+1,v_3+1) \end{aligned}$$

for $$i=0,1,2,3$$.

Recall we can also find a relation between various $(v_0,v_1,v_2,v_3)$ by differentiating with respect to $\psi$. Rewriting (2.2.3) in the current notation, we obtain

2.3.6 $$\begin{aligned} \frac{\mathrm {d}}{\mathrm {d}\psi } (v_0,v_1,v_2,v_3) = (4(k({\mathbf {v}}) +1)) (v_0+1,v_1+1,v_2+1,v_3+1), \end{aligned}$$

yielding a dependence of the monomials with respect to the successive derivatives with respect to $\psi$.

Using the relations (2.3.3) and (2.3.6), we will compute the Picard–Fuchs equations associated to periods that come from primitive cohomology. The key observation is that these two operations respect the symplectic symmetry group.

Restricting now to our situation, let $\diamond \in \{{\mathsf {F}}_4,{\mathsf {F}}_2{\mathsf {L}}_2,{\mathsf {F}}_1{\mathsf {L}}_3,{\mathsf {L}}_2{\mathsf {L}}_2,{\mathsf {L}}_4\}$ signify one of the five K3 families in (1.2.1) defined by $F_{\diamond ,\psi }$ and having symmetry group $H=H_{\diamond }$ as in (1.2.1). Then *H* acts on the 19-dimensional ${\mathbb {C}}$-vector space

2.3.7 $$\begin{aligned} V :=({\mathbb {C}}[x_0, x_1,x_2, x_3] / J(F_\psi ))_4 \end{aligned}$$

giving a representation $H \rightarrow {{\,\mathrm{GL}\,}}(V)$. As *H* is abelian, we may decompose $V = \bigoplus _\chi W_\chi$ where *H* acts on $W_\chi$ by a (one-dimensional) character $\chi :H \rightarrow {\mathbb {C}}^\times$. Conveniently, each subspace $W_{\chi }$ has a monomial basis. Moreover, the relations from the Jacobian ideal (2.3.3) and (2.3.6) respect the action of *H*, so we can apply the Griffiths–Dwork technique to the smaller subspaces $W_\chi$.

### Hypergeometric differential equations

In fact, we will find that all of our Picard–Fuchs differential equations are hypergeometric. In this section, we briefly recall the definitions [48].

#### Definition 2.4.1

Let $n,m\in {\mathbb {Z}}$, let $\alpha _1,\dots ,\alpha _n \in {\mathbb {Q}}$ and $\beta _1,\dots ,\beta _m\in {\mathbb {Q}}_{>0}$, and write $\varvec{\alpha }=\{\alpha _j\}_j$ and $\varvec{\beta }=\{\beta _j\}_j$ as multisets. The *(generalized) hypergeometric function* is the formal series

2.4.2 $$\begin{aligned} F(\varvec{\alpha };\varvec{\beta } \,|\, z) :=\sum _{k=0}^{\infty }\frac{(\alpha _1)_k\cdots (\alpha _n)_k}{(\beta _1)_k\cdots (\beta _m)_k}z^k \in {\mathbb {Q}}[[z]], \end{aligned}$$

where $(x)_k$ is the rising factorial (or *Pochhammer symbol*)

$$\begin{aligned} (x)_k :=x(x+1)\cdots (x+k-1)=\frac{\Gamma (x+k)}{\Gamma (x)} \end{aligned}$$

and $(x)_0 :=1$. We call $\varvec{\alpha }$ the *numerator parameters* and $\varvec{\beta }$ the *denominator parameters*.

We consider the differential operator

$$\begin{aligned} \theta :=z \displaystyle {\frac{\mathrm {d}}{\mathrm {d}z}} \end{aligned}$$

and define the *hypergeometric differential operator*

2.4.3 $$\begin{aligned} D(\varvec{\alpha };\varvec{\beta } \,|\,z) :=(\theta +\beta _1 -1)\cdots (\theta + \beta _m -1) - z (\theta +\alpha _1)\cdots (\theta + \alpha _n). \end{aligned}$$

When $\beta _1=1$, the hypergeometric function $F(\varvec{\alpha };\varvec{\beta } \,|\, z)$ is annihilated by $D(\varvec{\alpha };\varvec{\beta }\,|\,z)$.

### The Dwork pencil ${\mathsf {F}}_4$

We now proceed to calculate Picard–Fuchs equations for our five pencils. We begin in this section with the Dwork pencil ${\mathsf {F}}_4$, the one-parameter family of projective hypersurfaces $X_\psi \subset {\mathbb {P}}^4$ defined by the vanishing of the polynomial

$$\begin{aligned} F_{ \psi } :=x_0^4+x_1^4 + x_2^4 + x_3^4 - 4\psi x_0x_1x_2x_3. \end{aligned}$$

The differential equations associated to this pencil were studied by Dwork [20, §6j]; our approach is a bit more detailed and explicit, and this case is a good warmup as the simplest of the five cases we will consider.

There is a $H=({\mathbb {Z}}/4{\mathbb {Z}})^2$ symmetry of this family generated by the automorphisms

2.5.1 $$\begin{aligned} \begin{aligned} g_1(x_0:x_1:x_2:x_3)&= (-\sqrt{-1}x_0:\sqrt{-1}x_1:x_2:x_3), \\ g_2(x_0:x_1:x_2:x_3)&= (-\sqrt{-1}x_0:x_1:\sqrt{-1}x_2:x_3). \end{aligned} \end{aligned}$$

A character $\chi :H \rightarrow {\mathbb {C}}^\times$ is determined by $\chi (g_1),\chi (g_2) \in \langle \sqrt{-1} \rangle$, and we write $\chi _{(a_1,a_2)}$ for the character with $\chi _{(a_1,a_2)}(g_i) = \sqrt{-1}^{a_i}$ with $a_i \in {\mathbb {Z}}/4{\mathbb {Z}}$ for $i=1,2$, totaling 16 characters. We then decompose *V* defined in (2.3.7) into irreducible subspaces with a monomial basis. We cluster these subspaces into three types up to the permutation action by $S_4$ on coordinates:

(i) $(a_1,a_2)=(0,0)$ (the *H*-invariant subspace), spanned by $x_0x_1x_2x_3$;
(ii) $(a_1,a_2)$ both even but not both zero, e.g., the subspace with $(a_1,a_2)=(0,2)$ spanned by $x_0^2x_1^2, x_2^2 x_3^2$; and
(iii) $(a_1,a_2)$ not both even, e.g., the subspace with $(a_1,a_2)=(2,1)$, spanned by $x_0^3x_1$.

Up to permutation of coordinates, there are 1, 3, 12 subspaces of types (i),(ii),(iii), respectively. By symmetry, we just need to compute the Picard–Fuchs equations associated to one subspace of each of these types. In other words, we only need to find equations satisfied by the monomials $$x_0x_1x_2x_3, x_0^2x_1^2, x_2^2 x_3^2, $$ and $$x_0^3x_1$$, corresponding to (1, 1, 1, 1), (2, 2, 0, 0), (0, 0, 2, 2), and (3, 1, 0, 0), respectively.

The main result for this subsection is as follows.

#### Proposition 2.5.2

The primitive middle-dimensional cohomology group $H^2_{\text {prim}}(X_{{\mathsf {F}}_4, \psi },{\mathbb {C}})$ has 21 periods whose Picard–Fuchs equations are hypergeometric differential equations as follows:

$$\begin{aligned} \begin{aligned} 3 \text { periods are annihilated by}\,D\left( \tfrac{1}{4}, \tfrac{1}{2}, \tfrac{3}{4} ; 1, 1, 1 \,|\,\psi ^{-4}\right) , \\ 6 \text { periods are annihilated by}\,D\left( \tfrac{1}{4}, \tfrac{3}{4}; 1, \tfrac{1}{2} \,|\,\psi ^{-4}\right) , \text {and} \\ 12 \text { periods are annihilated by}\,D\left( \tfrac{1}{2};1 \,|\,\psi ^{-4}\right) . \end{aligned} \end{aligned}$$

By the interlacing criterion [4, Theorem 4.8], the latter two hypergeometric equations have algebraic solutions.

We state and prove each case of Proposition 2.5.2 with an individual lemma.

#### Lemma 2.5.3

The Picard–Fuchs equation associated to the period $\psi (0,0,0,0)$ is the hypergeometric differential equation $D(\tfrac{1}{4}, \tfrac{1}{2}, \tfrac{3}{4} ; 1, 1, 1 \,|\,\psi ^{-4})$.

#### Proof

We recall Eqs. (2.3.5) and (2.3.6):

2.5.4 $$\begin{aligned} (v_0,\ldots , v_i + 4, \ldots , v_3) = \frac{1+v_i}{4(k({\mathbf {v}})+1)} (v_0,v_1,v_2,v_3) + \psi (v_0+1, v_1+1, v_2+1,v_3+1); \end{aligned}$$

2.5.5 $$\begin{aligned} \frac{\mathrm {d}}{\mathrm {d}\psi } (v_0,v_1,v_2,v_3) = (4(k({\mathbf {v}}) +1)) (v_0+1,v_1+1,v_2+1,v_3+1). \end{aligned}$$

These equations imply a dependence among the terms

$$\begin{aligned} (v_0,v_1,v_2,v_3), (v_0+1,v_1+1,v_2+1,v_3+1), \text { and }(v_0,\ldots , v_i + 4, \ldots , v_3) \end{aligned}$$

denoted in the following diagram:In order to use these dependences, we build up a larger diagram:2.5.6It may be useful to point out that the same period must appear in two places by simple linear algebra: The vectors (4, 0, 0, 0), (0, 4, 0, 0), (0, 0, 4, 0), (0, 0, 0, 4) and (1, 1, 1, 1) are linearly dependent.

Using (2.3.5) and (2.3.6) and letting $\eta :=\psi \displaystyle {\frac{\mathrm {d}}{\mathrm {d}\psi }}$, we see that

2.5.7 $$\begin{aligned} \begin{aligned} (0,0,0,0)&= \frac{1}{4} (\eta + 1) (4,0,0,0), \\ (4,0,0,0)&= \frac{1}{8} (\eta + 1) (4,4,0,0), \\ (4,4,0,0)&= \frac{1}{12} (\eta + 1) (4,4,4,0), \\ \psi (4,4,4,0)&= (3,3,3,3). \end{aligned} \end{aligned}$$

Now, we can use the fact that $(\eta - a)\psi ^a = \psi ^a \eta$ for $a \in {\mathbb {Z}}$ to great effect:

2.5.8 $$\begin{aligned} \begin{aligned} \eta (0,0,0,0)&= 4 \psi (1,1,1,1), \\ (\eta - 1)\eta (0,0,0,0)&= 4 \psi \eta (1,1,1,1) = 8\cdot 4 \psi ^2 (2,2,2,2), \\ (\eta -2)(\eta -1)\eta (0,0,0,0)&=12 \cdot 8\cdot 4 \psi ^2 \eta (2,2,2,2) = 12\cdot 8\cdot 4 \psi ^3 (3,3,3,3) \\&= 12\cdot 8\cdot 4 \psi ^4 (4,4,4,0) \\&= 8 \cdot 4 \psi ^4 (\eta +1)(4,4,0,0) \\&= 4\psi ^4(\eta +1)^2 (4,0,0,0) \\&= \psi ^4(\eta +1)^3 (0,0,0,0). \end{aligned} \end{aligned}$$

We conclude that

2.5.9 $$\begin{aligned} \left[ (\eta -2)(\eta -1)\eta - \psi ^4 (\eta +1)^3\right] (0,0,0,0) = 0. \end{aligned}$$

We then multiply by $\psi$ to obtain

$$\begin{aligned} \left[ \psi (\eta -2)(\eta -1)\eta - \psi ^4 \psi (\eta +1)^3\right] (0,0,0,0)&= 0,\\ \left[ (\eta -3)(\eta -2)(\eta -1) - \psi ^4 (\eta )^3\right] \psi (0,0,0,0)&= 0. \end{aligned}$$

Finally, substitute $t :=\psi ^{-4}$ and let $\theta :=t \displaystyle {\frac{\mathrm {d}}{\mathrm {d}t}} = -\eta /4$ to see that

2.5.10 $$\begin{aligned} \begin{aligned} \left[ \left( -4\theta -3\right) \left( -4\theta -2\right) \left( -4\theta -1\right) - t^{-1} \left( -4\theta \right) ^3\right] \psi \left( 0,0,0,0\right)&= 0,\\ \left[ -t\left( \theta +\tfrac{3}{4}\right) \left( \theta +\tfrac{1}{2}\right) \left( \theta +\tfrac{1}{4}\right) + \theta ^3\right] \psi \left( 0,0,0,0\right)&= 0,\\ \left[ \theta ^3 -t\left( \theta +\tfrac{1}{4}\right) \left( \theta +\tfrac{1}{2}\right) \left( \theta +\tfrac{3}{4}\right) \right] \psi \left( 0,0,0,0\right)&= 0, \end{aligned} \end{aligned}$$

which is the differential equation $D(\tfrac{1}{4}, \tfrac{1}{2}, \tfrac{3}{4} ; 1, 1, 1 \,|\,t)$. $\square$

#### Lemma 2.5.11

The Picard–Fuchs equation associated to both $\psi (2,2,0,0)$ and $\psi (0,0,2,2)$ is $D(\tfrac{1}{4}, \tfrac{3}{4}; 1, \tfrac{1}{2} \,|\, \psi ^{-4})$.

#### Proof

By iterating the use of (2.3.5), we can construct a diagram including both (2, 2, 0, 0) and (0, 0, 2, 2):2.5.12We then obtain the following relations:

2.5.13 $$\begin{aligned} \begin{aligned} \eta (2,2,0,0)&= \psi ^2(\eta + 1) (0,0,2,2), \\ \eta (0,0,2,2)&= \psi ^2(\eta + 1) (2,2,0,0). \end{aligned} \end{aligned}$$

We then can use these relations to make a Picard–Fuchs equation associated to the period (2, 2, 0, 0):

2.5.14 $$\begin{aligned} \begin{aligned} (\eta - 2) \eta (2,2,0,0)&= \psi ^2 (\eta + 1)\eta (0,0,2,2) \\&= \psi ^2(\eta +1) \left( \psi ^2(\eta +1) (2,2,0,0)\right) \\&= 2\psi ^4(\eta +1) (2,2,0,0) + \psi ^4(\eta +1) \eta (2,2,0,0) \\&\qquad + \psi ^4(\eta +1) (2,2,0,0) \\&= \psi ^4(\eta ^2+4\eta +3) (2,2,0,0) \\&= \psi ^4(\eta +1)(\eta +3) (2,2,0,0). \end{aligned} \end{aligned}$$

By symmetry, we get the same equation for the period (0, 0, 2, 2), so we have

2.5.15 $$\begin{aligned} \begin{aligned} \left[ (\eta -2)\eta - \psi ^4(\eta +1)(\eta +3)\right] (2,2,0,0)&= 0, \\ \left[ (\eta -2)\eta - \psi ^4(\eta +1)(\eta +3) \right] (0,0,2,2)&= 0. \end{aligned} \end{aligned}$$

Now multiply by $\psi$ and then change variables to $t :=\psi ^{-4}$ with $\theta :=t \tfrac{\mathrm {d}}{\mathrm {d}t} = -{4} \eta$ to obtain

$$\begin{aligned} \left[ \psi \left( \eta -2\right) \eta - \psi \psi ^4\left( \eta +1\right) \left( \eta +3\right) \right] \left( 2,2,0,0\right)&= 0,\\ \left[ \left( \eta -3\right) \left( \eta -1\right) - \psi ^4\eta \left( \eta +2\right) \right] \psi \left( 2,2,0,0\right)&= 0,\\ \left[ \left( -4\theta -3\right) \left( -4\theta -1\right) - t^{-1}\left( -4\theta \right) \left( -4\theta +2\right) \right] \psi \left( 2,2,0,0\right)&= 0, \\ \left[ t\left( \theta + \tfrac{3}{4}\right) \left( \theta + \tfrac{1}{4}\right) - \theta \left( \theta -\tfrac{1}{2}\right) \right] \psi \left( 2,2,0,0\right)&= 0, \\ \left[ \theta \left( \theta -\tfrac{1}{2}\right) -t\left( \theta + \tfrac{1}{4}\right) \left( \theta +\tfrac{3}{4}\right) \right] \psi \left( 2,2,0,0\right)&= 0. \end{aligned}$$

This Picard–Fuchs equation is $D(\tfrac{1}{4}, \tfrac{3}{4}; 1, \tfrac{1}{2} \,|\, \psi ^{-4})$. $\square$

#### Lemma 2.5.16

The Picard–Fuchs equation associated to $\psi (3,1,0,0)$ is $D(\frac{1}{2};1 \,|\,\psi ^{-4})$.

#### Proof

Our strategy again is to use (2.3.5) and (2.3.6) in the order represented by the diagram below to study the period (3, 1, 0, 0):2.5.17Using (2.3.5) iteratively in the upper part of the diagram, we see that

2.5.18 $$\begin{aligned} (1,3,2,2) = \psi ^2(3,1,0,4). \end{aligned}$$

Then using (2.3.6), we then have that

2.5.19 $$\begin{aligned} \eta (0,2,1,1) = 8\psi (1,3,2,2) = 8\psi ^3 (3,1,0,4). \end{aligned}$$

Now, using (2.3.5) again, we have that $(3,1,0,0) = \psi (0,2,1,1)$ and we can then compute:

2.5.20 $$\begin{aligned} \begin{aligned} (\eta -1) (3,1,0,0)&= \psi \eta (0,2,1,1) \\&=8\psi ^4(3,1,0,4) \\&=8 \psi ^4\left[ \frac{1}{8} (3,1,0,0) + \psi (4,2,1,1)\right] \\&= 8 \psi ^4 \left[ \frac{1}{8} (3,1,0,0) + \frac{1}{8} \eta (3,1,0,0)\right] \\&= \psi ^4 (\eta + 1) (3,1,0,0). \end{aligned} \end{aligned}$$

We then get the Picard–Fuchs equation associated to the period (3, 1, 0, 0):

2.5.21 $$\begin{aligned} \left[ (\eta - 1) - \psi ^4 (\eta +1) \right] (3,1,0,0) = 0. \end{aligned}$$

We now will multiply by $\psi$ and then change variables to $t = \psi ^{-4}$ as in the previous lemma to obtain:

$$\begin{aligned} \left[ \psi \left( \eta - 1\right) - \psi \psi ^4 \left( \eta +1\right) \right] \left( 3,1,0,0\right)&= 0,\\ \left[ \left( \eta - 2\right) - \psi ^4 \eta \right] \psi \left( 3,1,0,0\right)&= 0,\\ \left[ \left( -4\theta - 2\right) - t^{-1} \left( -4\theta \right) \right] \psi \left( 3,1,0,0\right)&= 0, \\ \left[ \theta -t\left( \theta + \tfrac{1}{2}\right) \right] \psi \left( 3,1,0,0\right)&= 0, \end{aligned}$$

giving rise to the hypergeometric differential equation $D(\frac{1}{2};1 \,|\,\psi ^{-4})$. $\square$

We now conclude this section with the proof of the main result.

#### Proof of Proposition 2.5.2

We combine Lemmas 2.5.3, 2.5.11, and 2.5.16 with the consideration of the number of subspaces of each type described above.

### The Klein–Mukai pencil ${\mathsf {F}}_1{\mathsf {L}}_3$

We now consider the Klein–Mukai pencil ${\mathsf {F}}_1{\mathsf {L}}_3$, the one-parameter family of hypersurfaces $X_\psi \subset {\mathbb {P}}^4$ defined by the vanishing of

$$\begin{aligned} F_\psi :=x_0^3x_1+ x_1^3x_2 + x_2^3x_0 + x_3^4 - 4\psi x_0x_1x_2x_3. \end{aligned}$$

The polynomial $F_\psi$ is related to the defining polynomial (1.2.1) by a change in the order of variables.

There is a $H={\mathbb {Z}}/ 7{\mathbb {Z}}$ scaling symmetry of this family generated by the automorphism $(x_i)$ by the element

$$\begin{aligned} g(x_0: x_1: x_2: x_3) = (\xi x_0 : \xi ^4 x_1 : \xi ^2x_2 : x_3), \end{aligned}$$

where $\xi$ is a seventh root of unity. There are seven characters $\chi _k:H \rightarrow {\mathbb {C}}^\times$ defined by $\chi _k(g) = \xi ^k$ for $k \in {\mathbb {Z}}/7{\mathbb {Z}}$. Note that the monomial bases for the subspaces $W_{\chi _1}, W_{\chi _2},$ and $W_{\chi _4}$ are cyclic permutations of one another under the variables $x_0, x_1,$ and $x_2$. Analogously, so are subspaces $W_{\chi _3}, W_{\chi _5},$ and $W_{\chi _6}$. So we have three types of clusters:

(i) $W_{\chi _0}$ has the monomial basis $\{x_0x_1x_2x_3\}$;
(ii) $W_{\chi _1}$ has the monomial basis $\{x_1^2x_3^2, x_0^2x_1x_2, x_2^2x_1x_3\}$; and
(iii) $W_{\chi _3}$ has the monomial basis $\{x_2^3x_1, x_1^2x_2x_3, x_3^2x_0x_2\}$.

There is one cluster of type (i) and three clusters each of types (ii) and (iii), so $h^{1,1}$ is decomposed as $19 = 1 + 3\cdot 3 + 3 \cdot 3$.

#### Proposition 2.6.1

The group $H^2_{\text {prim}}(X_{{\mathsf {F}}_1{\mathsf {L}}_3, \psi })$ has 21 periods whose Picard–Fuchs equations are hypergeometric differential equations, with 3 periods annihilated by

$$\begin{aligned} D\left( \tfrac{1}{4}, \tfrac{1}{2}, \tfrac{3}{4} ; 1, 1, 1 \,|\,\psi ^{-4}\right) \end{aligned}$$

and 3 periods each annihilated by the following 6 operators:

$$\begin{aligned}&D\left( \tfrac{1}{14}, \tfrac{9}{14}, \tfrac{11}{14} ; \tfrac{1}{4}, \tfrac{3}{4}, 1 \,|\,\psi ^{4}\right) , D\left( \tfrac{-3}{14}, \tfrac{1}{14}, \tfrac{9}{14} ; 0, \tfrac{1}{4}, \tfrac{3}{4} \,|\,\psi ^{4}\right) , D\left( \tfrac{-5}{14}, \tfrac{-3}{14}, \tfrac{1}{14} ; \tfrac{-1}{4}, 0, \tfrac{1}{4} \,|\,\psi ^{4}\right) , \\&D\left( \tfrac{3}{14}, \tfrac{5}{14}, \tfrac{13}{14} ; \tfrac{1}{4}, \tfrac{3}{4}, 1 \,|\,\psi ^4\right) , D\left( \tfrac{-1}{14}, \tfrac{3}{14}, \tfrac{5}{14} ; 0, \tfrac{1}{4}, \tfrac{3}{4} \,|\,\psi ^4\right) , D\left( \tfrac{-11}{14}, \tfrac{-1}{14}, \tfrac{5}{14} ; \tfrac{-1}{4}, 0, \tfrac{1}{4} \,|\,\psi ^4\right) . \end{aligned}$$

Again, the latter 6 operators have an algebraic solution. To prove Proposition 2.6.1, we again use the diagrammatic method outlined above, but in this case we have different periods that are related. Notice that we have the following differentials $\partial _i$ multiplied by $x_i$:

2.6.2 $$\begin{aligned} \begin{aligned} x_0\partial _0 F_\psi&= 3 x_0^3 x_1 + x_2^3x_0 - 4 \psi x_0x_1x_2x_3, \\ x_1\partial _1 F_\psi&= 3 x_1^3 x_2 + x_0^3x_1 - 4 \psi x_0x_1x_2x_3, \\ x_2\partial _2 F_\psi&= 3 x_2^3 x_0 + x_1^3x_2 - 4 \psi x_0x_1x_2x_3, \\ x_3 \partial _3 F_\psi&= 4x_4^4 - 4\psi x_0x_1x_2x_3. \end{aligned} \end{aligned}$$

We can make linear combinations of these equations so that the right-hand side is just a linear combination of two monomials, for example,

2.6.3 $$\begin{aligned} (9x_0\partial _0 + x_1\partial _1 - 3x_2\partial _2)F_\psi = 28(x_0^3x_1 - \psi x_0x_1x_2x_3). \end{aligned}$$

Now using (2.3.3), we obtain the following period relations analogous to (2.3.5), written in multi-index notation:

2.6.4 $$\begin{aligned} \begin{aligned} {\mathbf {v}}+(3,1,0,0)&= \frac{f_0({\mathbf {v}})}{28(k({\mathbf {v}})+1)}{\mathbf {v}} + \psi ({\mathbf {v}}+(1,1,1,1)), \\ {\mathbf {v}}+(0,3,1,0)&= \frac{f_1({\mathbf {v}})}{28(k({\mathbf {v}})+1)}{\mathbf {v}} + \psi ({\mathbf {v}}+(1,1,1,1)), \\ {\mathbf {v}}+(0,0,3,1)&= \frac{f_2({\mathbf {v}})}{28(k({\mathbf {v}})+1)}{\mathbf {v}} + \psi ({\mathbf {v}}+(1,1,1,1)), \text { and} \\ {\mathbf {v}}+(0,0,0,4)&= \frac{1+v_3}{4(k({\mathbf {v}})+1)}{\mathbf {v}} + \psi ({\mathbf {v}}+(1,1,1,1)), \end{aligned} \end{aligned}$$

where

2.6.5 $$\begin{aligned} \begin{aligned} f_0({\mathbf {v}})&:=9(v_0+1) +(v_1+1) - 3(v_2+1), \\ f_1({\mathbf {v}})&:=-3(v_0+1) +9(v_1+1) + (v_2+1), \text { and} \\ f_2({\mathbf {v}})&:=(v_0+1) -3 (v_1+1) + 9(v_2+1). \end{aligned} \end{aligned}$$

#### Lemma 2.6.6

Let $t = \psi ^{-4}$. The Picard–Fuchs equation associated to the period $\psi (0,0,0,0)$ is the hypergeometric differential equation $D(\tfrac{1}{4}, \tfrac{1}{2}, \tfrac{3}{4} ; 1, 1, 1 \,|\,t)$.

#### Proof

We build the following diagram using (2.6.4) and (2.3.6):When one runs through this computation, one can see that we get the same Picard–Fuchs equation for the invariant period as we did with the Fermat:

2.6.7 $$\begin{aligned} \left[ (\eta -2)(\eta -1)\eta - \psi ^4 (\eta +1)^3\right] (0,0,0,0) = 0. \end{aligned}$$

By multiplying by $\psi$ and changing variables to $t = \psi ^{-4}$ and $\theta = t \displaystyle {\frac{\mathrm {d}}{\mathrm {d}t}}$, we can see by following through the computation seen in  (2.5.10) that

$$\begin{aligned} \left[ \theta ^3 -t\left( \theta +\tfrac{3}{4}\right) \left( \theta +\tfrac{1}{2}\right) \left( \theta +\tfrac{1}{4}\right) \right] \psi \left( 0,0,0,0\right) = 0, \end{aligned}$$

which is the differential equation $D(\tfrac{1}{4}, \tfrac{1}{2}, \tfrac{3}{4} ; 1, 1, 1 \,|\,t)$. $\square$

#### Lemma 2.6.8

For the Klein–Mukai family $X_\psi$,

$$\begin{aligned} \begin{aligned} \text {the period }\left( 0,1,2,1\right) \text {is annihilated by } D\left( \tfrac{1}{14}, \tfrac{9}{14}, \tfrac{11}{14} ; \tfrac{1}{4}, \tfrac{3}{4}, 1 \,|\,\psi ^{4}\right) , \\ \text {the period } \psi \left( 0,2,0,2\right) \text {is annihilated by } D\left( \tfrac{-3}{14}, \tfrac{1}{14}, \tfrac{9}{14} ; 0, \tfrac{1}{4}, \tfrac{3}{4} \,|\,\psi ^{4}\right) , \text {and} \\ \text {the period } \psi ^3 \left( 2,1,1,0\right) \text {is annihilated by } D\left( \tfrac{-5}{14}, \tfrac{-3}{14}, \tfrac{1}{14} ; \tfrac{-1}{4}, 0, \tfrac{1}{4} \,|\,\psi ^{4}\right) . \end{aligned} \end{aligned}$$

#### Proof

For the character $\chi _1(g)=\xi$ associated to $1 \in {\mathbb {Z}}/7{\mathbb {Z}}$, we have the following diagram:We then have the following relations:

2.6.9 $$\begin{aligned} \begin{aligned} \eta (0,2,0,2)&= \frac{11}{7} \psi ^2 + \psi ^2 \eta (2,1,1,0), \\ \eta (2,1,1,0)&= \frac{2}{7} \psi (0,1,2,1) + \psi \eta (0,1,2,1), \\ \eta (0,1,2,1)&= \frac{1}{7} \psi (0,2,0,2) + \psi \eta (0,2,0,2). \end{aligned} \end{aligned}$$

Now, we can use these relations to compute the Picard–Fuchs equations associated to (2, 1, 1, 0), (0, 2, 0, 2),  and (0, 1, 2, 1). We first do this for the period (0, 1, 2, 1):

2.6.10 $$\begin{aligned} \eta (0,1,2,1)&= \frac{1}{7} \psi (0,2,0,2) \psi \eta (0,2,0,2), \nonumber \\ (\eta -1)\eta (0,1,2,1)&= \frac{165}{49} \psi ^3 (2,1,1,0) + \frac{26}{7} \psi ^3 \eta (2,1,1,0) + \psi ^3 \eta ^2 (2,1,1,0), \nonumber \\ (\eta -3)(\eta -1) \eta (0,1,2,1)&= \psi ^4\left( \eta + \tfrac{2}{7}\right) \left( \eta + \tfrac{18}{7}\right) \left( \eta + \tfrac{22}{7}\right) . \end{aligned}$$

This gives us the Picard–Fuchs equation for the period (0, 1, 2, 1):

2.6.11 $$\begin{aligned} \left[ (\eta -3)(\eta -1) \eta - \psi ^4\left( \eta + \tfrac{2}{7}\right) \left( \eta + \tfrac{18}{7}\right) \left( \eta + \tfrac{22}{7}\right) \right] (0,1,2,1) = 0. \end{aligned}$$

Letting $u=\psi ^4$ and $\sigma = u \displaystyle {\frac{\mathrm {d}}{\mathrm {d}u}}$, we get the following hypergeometric form:

$$\begin{aligned} \left[ (4\sigma -3)(4\sigma -1) 4\sigma - u\left( 4\sigma + \tfrac{2}{7}\right) \left( 4\sigma + \tfrac{18}{7}\right) \left( 4\sigma + \tfrac{22}{7}\right) \right] (0,1,2,1)&= 0,\\ \left[ \left( \sigma -\tfrac{3}{4}\right) \left( \sigma -\tfrac{1}{4}\right) \sigma - u\left( \sigma + \tfrac{1}{14}\right) \left( \sigma + \tfrac{9}{14}\right) \left( \sigma + \tfrac{11}{14}\right) \right] (0,1,2,1)&= 0, \end{aligned}$$

which is the hypergeometric differential equation $D(\tfrac{1}{14}, \tfrac{9}{14}, \tfrac{11}{14} ; 1, \tfrac{1}{4}, \tfrac{3}{4} \,|\,u)$.

We then do the same for (0, 2, 0, 2):

2.6.12 $$\begin{aligned} \eta (0,2,0,2)&=\frac{11}{7} \psi ^2 + \psi ^2 \eta (2,1,1,0), \nonumber \\ (\eta - 2) \eta (0,2,0,2)&= \frac{36}{49} \psi ^3 (0,1,2,1) + \frac{20}{7} \psi ^3 \eta (0,1,2,1) + \psi ^3 \eta ^2 (0,1,2,1), \nonumber \\ (\eta -3)(\eta -2) \eta (0,2,0,2)&= \psi ^4\left( \eta + \tfrac{1}{7} \right) \left( \eta + \tfrac{9}{7}\right) \left( \eta + \tfrac{25}{7}\right) (0,2,0,2). \end{aligned}$$

This gives us the Picard–Fuchs equation for the period (0, 2, 0, 2):

2.6.13 $$\begin{aligned} \left[ (\eta -3)(\eta -2)\eta - \psi ^4\left( \eta + \tfrac{1}{7} \right) \left( \eta + \tfrac{9}{7}\right) \left( \eta + \tfrac{25}{7}\right) \right] (0,2,0,2) = 0. \end{aligned}$$

By multiplying by $\psi$ and changing variables to $u=\psi ^4$ and $\sigma = u \displaystyle {\frac{\mathrm {d}}{\mathrm {d}u}}$, we get

$$\begin{aligned} \psi \left[ (\eta -3)(\eta -2)\eta - \psi ^4\left( \eta + \tfrac{1}{7} \right) \left( \eta + \tfrac{9}{7}\right) \left( \eta + \tfrac{25}{7}\right) \right] (0,2,0,2)&= 0, \\ \left[ (\eta -4)(\eta -3)(\eta -1) - \psi ^4\left( \eta - \tfrac{6}{7} \right) \left( \eta + \tfrac{2}{7}\right) \left( \eta + \tfrac{18}{7}\right) \right] \psi (0,2,0,2)&= 0, \\ \left[ (4\sigma -4)(4\sigma -3)(4\sigma -1) - u\left( 4\sigma - \tfrac{6}{7} \right) \left( 4\sigma + \tfrac{2}{7}\right) \left( 4\sigma + \tfrac{18}{7}\right) \right] \psi (0,2,0,2)&= 0, \\ \left[ (\sigma -1)\left( \sigma -\tfrac{3}{4}\right) \left( \sigma -\tfrac{1}{4}\right) - u\left( \sigma - \tfrac{3}{14} \right) \left( \sigma + \tfrac{1}{14}\right) \left( \sigma + \tfrac{9}{14}\right) \right] \psi (0,2,0,2)&= 0, \end{aligned}$$

which is the hypergeometric differential equation $D(\tfrac{1}{14}, \tfrac{9}{14}, \tfrac{-3}{14} ; 0, \tfrac{1}{4}, \tfrac{3}{4} \,|\,u)$.

We finally look at (2, 1, 1, 0):

2.6.14 $$\begin{aligned} \begin{aligned} \eta (2,1,1,0)&= \frac{2}{7} \psi (0,1,2,1) + \psi \eta (0,1,2,1) \\ (\eta -1)\eta (2,1,1,0)&= \frac{2}{7} \psi \eta (0,1,2,1) + \psi \eta ^2 (0,1,2,1), \\&= \frac{9}{49} \psi ^2 (0,2,0,2) + \frac{10}{7} \psi ^2 \eta (0,2,0,2) + \psi ^2 \eta ^2 (0,2,0,2) \\ (\eta -2)(\eta -1)\eta (2,1,1,0)&=\frac{9}{49} \psi ^2\eta (0,2,0,2) + \frac{10}{7} \psi ^2 \eta ^2 (0,2,0,2) + \psi ^2 \eta ^3 (0,2,0,2), \\&= \psi ^4\left( \eta + \tfrac{11}{7}\right) \left( \eta + \tfrac{15}{7}\right) \left( \eta + \tfrac{23}{7}\right) (2,1,1,0). \end{aligned} \end{aligned}$$

This gives us the Picard-Fuchs equation for the period (2, 1, 1, 0):

2.6.15 $$\begin{aligned} \left[ (\eta -2)(\eta -1)\eta - \psi ^4 \left( \eta + \tfrac{11}{7}\right) \left( \eta + \tfrac{15}{7}\right) \left( \eta + \tfrac{23}{7}\right) \right] (2,1,1,0) = 0. \end{aligned}$$

By multiplying by $\psi ^3$ and again changing variables, we get:

$$\begin{aligned} \psi ^3\left[ \left( \eta -2\right) \left( \eta -1\right) \eta - \psi ^4 \left( \eta + \tfrac{11}{7}\right) \left( \eta + \tfrac{15}{7}\right) \left( \eta + \tfrac{23}{7}\right) \right] \left( 2,1,1,0\right)&= 0, \\ \left[ \left( \eta -5\right) \left( \eta -4\right) \left( \eta -3\right) - \psi ^4 \left( \eta - \tfrac{10}{7}\right) \left( \eta - \tfrac{6}{7}\right) \left( \eta + \tfrac{2}{7}\right) \right] \psi ^3\left( 2,1,1,0\right)&= 0, \\ \left[ \left( 4\sigma -5\right) \left( 4\sigma -4\right) \left( 4\sigma -3\right) - u \left( 4\sigma - \tfrac{10}{7}\right) \left( 4\sigma - \tfrac{6}{7}\right) \left( 4\sigma + \tfrac{2}{7}\right) \right] \psi ^3\left( 2,1,1,0\right)&= 0, \\ \left[ \left( \sigma -\tfrac{5}{4}\right) \left( \sigma -1\right) \left( \sigma -\tfrac{3}{4}\right) - u \left( \sigma - \tfrac{5}{14}\right) \left( \sigma - \tfrac{3}{14}\right) \left( \sigma + \tfrac{1}{14}\right) \right] \psi ^3\left( 2,1,1,0\right)&= 0. \end{aligned}$$

At last, we have the hypergeometric differential equation $D(\tfrac{1}{14}, \tfrac{-5}{14}, \tfrac{-3}{14} ; 0, \tfrac{-1}{4}, \tfrac{1}{4} \,|\,u)$. $\square$

#### Lemma 2.6.16

For the Klein–Mukai family $X_\psi$,

$$\begin{aligned} \begin{aligned} \text {the period} \left( 0,2,1,1\right) \text {is annihilated by } D\left( \tfrac{3}{14}, \tfrac{5}{14}, \tfrac{13}{14} ; \tfrac{1}{4}, \tfrac{3}{4}, 1 \,|\,\psi ^4\right) , \\ \text {the period } \psi \left( 1,0,1,2\right) \text {is annihilated by } D\left( \tfrac{-1}{14}, \tfrac{3}{14}, \tfrac{5}{14} ; 0, \tfrac{1}{4}, \tfrac{3}{4} \,|\,\psi ^4\right) , \text {and} \\ \text {the period } \psi ^3 \left( 0,1,3,0\right) \text {is annihilated by } D\left( \tfrac{-11}{14}, \tfrac{-1}{14}, \tfrac{5}{14} ; \tfrac{-1}{4}, 0, \tfrac{1}{4} \,|\,\psi ^4\right) . \end{aligned} \end{aligned}$$

#### Proof

We use the following diagram:and then compute the following period relations:

2.6.17 $$\begin{aligned} \begin{aligned} \eta (0,1,3,0)&= \frac{10}{7} \psi (0,2,1,1) + \psi \eta (0,2,1,1), \\ \eta (0,2,1,1)&= \frac{5}{7} \psi (1,0,1,2) + \psi \eta (1,0,1,2), \\ \eta (1,0,1,2)&= \frac{-1}{7} \psi ^2 (0,1,3,0) + \psi ^2 \eta (0,1,3,0). \end{aligned} \end{aligned}$$

By cyclically using these relations, we get the following Picard–Fuchs equations:

2.6.18 $$\begin{aligned} \begin{aligned} \left[ (\eta -3)(\eta -1)\eta - \psi ^4\left( \eta + \tfrac{6}{7}\right) \left( \eta + \tfrac{10}{7}\right) \left( \eta + \tfrac{26}{7}\right) \right] (0,2,1,1)&= 0,\\ \left[ (\eta -3)(\eta - 2) \eta - \psi ^4 \left( \eta + \tfrac{5}{7}\right) \left( \eta + \tfrac{13}{7}\right) \left( \eta + \tfrac{17}{7}\right) \right] (1,0,1,2)&=0, \\ \left[ (\eta -2)(\eta -1)\eta - \psi ^4\left( \eta - \tfrac{1}{7}\right) \left( \eta + \tfrac{19}{7}\right) \left( \eta + \tfrac{31}{7}\right) \right] (0,1,3,0)&=0. \end{aligned} \end{aligned}$$

We then multiply these equations above by $1, \psi ,$ and $\psi ^3$, respectively, and then change coordinates to $u = \psi ^4$ and $\sigma = u \displaystyle {\frac{\mathrm {d}}{\mathrm {d}u}}$ to obtain the following:

$$\begin{aligned} \left[ \left( \sigma -\tfrac{3}{4}\right) \left( \sigma -\tfrac{1}{4}\right) \sigma - u\left( \sigma + \tfrac{3}{14}\right) \left( \sigma + \tfrac{5}{14}\right) \left( \sigma + \tfrac{13}{14}\right) \right] \left( 0,2,1,1\right)&= 0,\\ \left[ \left( \sigma -1\right) \left( \sigma - \tfrac{3}{4}\right) \left( \sigma -\tfrac{1}{4}\right) - u \left( \sigma - \tfrac{1}{14}\right) \left( \sigma + \tfrac{3}{14}\right) \left( \sigma + \tfrac{5}{14}\right) \right] \psi \left( 1,0,1,2\right)&=0, \\ \left[ \left( \sigma -\tfrac{5}{4}\right) \left( \sigma -1\right) \left( \sigma -\tfrac{3}{4}\right) - u\left( \sigma - \tfrac{11}{14}\right) \left( \sigma - \tfrac{1}{14}\right) \left( \sigma + \tfrac{5}{14}\right) \right] \psi ^3 \left( 0,1,3,0\right)&=0, \end{aligned}$$

which are $D(\tfrac{3}{14}, \tfrac{5}{14}, \tfrac{13}{14} ; 1, \tfrac{1}{4}, \tfrac{3}{4} \,|\,u )$, $D(\tfrac{3}{14}, \tfrac{5}{14}, \tfrac{-1}{14} ; 0, \tfrac{1}{4}, \tfrac{3}{4} \,|\,u )$, and $D(\tfrac{-11}{14}, \tfrac{5}{14}, \tfrac{-1}{14} ; 0, \tfrac{1}{4}, \tfrac{-1}{4} \,|\,u)$, respectively. $\square$

We conclude this section by combining these results.

#### Proof of Proposition 2.6.1

Combine Lemmas  2.6.6, 2.6.8, and 2.6.16.

### Remaining pencils

For the remaining three pencils ${\mathsf {F}}_2{\mathsf {L}}_2$, ${\mathsf {L}}_2{\mathsf {L}}_2$, and ${\mathsf {L}}_4$, the Picard–Fuchs equations can be derived in a similar manner. The details can be found in Appendix A; we state here only the results.

#### Proposition 2.7.1

The group $H^2_{\text {prim}}(X_{{\mathsf {F}}_2{\mathsf {L}}_2, \psi }, {\mathbb {C}})$ has 15 periods whose Picard–Fuchs equations are hypergeometric differential equations as follows:

$$\begin{aligned} \begin{aligned} 3 \text { periods are annihilated by } D\left( \tfrac{1}{4}, \tfrac{1}{2}, \tfrac{3}{4} ; 1, 1, 1 \,|\,\psi ^{-4}\right) , \\ 2 \text { periods are annihilated by } D\left( \tfrac{1}{4}, \tfrac{3}{4} ; 1, \tfrac{1}{2} \,|\,\psi ^{-4}\right) , \\ 2 \text { periods are annihilated by } D\left( \tfrac{1}{2} ; 1 \,|\,\psi ^{4}\right) , \\ 4 \text { periods are annihilated by } D\left( \tfrac{1}{8}, \tfrac{5}{8} ; 1, \tfrac{1}{4} \,|\,\psi ^{4}\right) , \text {and } \\ 4 \text { periods are annihilated by } D\left( \tfrac{1}{8}, \tfrac{-3}{8} ; 0, \tfrac{1}{4} \,|\,\psi ^{4}\right) . \end{aligned} \end{aligned}$$

#### Proof

See Proposition A.1.2. $$\square $$

#### Proposition 2.7.2

The group $H^2_{\text {prim}}(X_{{\mathsf {L}}_2{\mathsf {L}}_2, \psi }, {\mathbb {C}})$ has 13 periods whose Picard–Fuchs equations are hypergeometric differential equations as follows:

$$\begin{aligned} \begin{aligned} 3 \text { periods are annihilated by } D\left( \tfrac{1}{4}, \tfrac{1}{2}, \tfrac{3}{4} ; 1, 1, 1 \,|\,\psi ^{-4}\right) , \\ 8 \text { periods are annihilated by } D\left( \tfrac{1}{8}, \tfrac{3}{8}, \tfrac{5}{8}, \tfrac{7}{8} ; 0, \tfrac{1}{4}, \tfrac{1}{2}, \tfrac{3}{4} \,|\,\psi ^{4}\right) , \text {and } \\ 2 \text { periods are annihilated by } D\left( \tfrac{1}{4}, \tfrac{3}{4} ; 1, \tfrac{1}{2} \,|\,\psi ^{4}\right) . \end{aligned} \end{aligned}$$

#### Proof

See Proposition A.2.2. $$\square $$

#### Proposition 2.7.3

The group $H^2_{\text {prim}}(X_{{\mathsf {L}}_4, \psi },{\mathbb {C}})$ has 19 periods whose Picard–Fuchs equations are hypergeometric differential equations as follows:

$$\begin{aligned} \begin{aligned} 3 \text { periods are annihilated by } D\left( \tfrac{1}{4}, \tfrac{1}{2}, \tfrac{3}{4} ; 1, 1, 1 \,|\,\psi ^{-4}\right) , \\ 4 \text { periods are annihilated by } D\left( \tfrac{1}{5}, \tfrac{2}{5}, \tfrac{3}{5}, \tfrac{4}{5}; 1, \tfrac{1}{4}, \tfrac{1}{2}, \tfrac{3}{4} \,|\, \psi ^4\right) , \\ 4 \text { periods are annihilated by } D\left( \tfrac{-1}{5}, \tfrac{1}{5}, \tfrac{2}{5}, \tfrac{3}{5}; 0, \tfrac{1}{4}, \tfrac{1}{2}, \tfrac{3}{4} \,|\,\psi ^4\right) , \\ 4 \text { periods are annihilated by } D\left( \tfrac{-2}{5}, \tfrac{-1}{5}, \tfrac{1}{5}, \tfrac{2}{5}; \tfrac{-1}{4}, 0, \tfrac{1}{4}, \tfrac{1}{2} \,|\,\psi ^4\right) , \text {and} \\ 4 \text { periods are annihilated by } D\left( \tfrac{-3}{5}, \tfrac{-2}{5}, \tfrac{-1}{5}, \tfrac{1}{5}; 0, \tfrac{1}{4}, \tfrac{-1}{2}, \tfrac{-1}{4} \,|\,\psi ^4\right) . \end{aligned} \end{aligned}$$

#### Proof

See Proposition A.3.2. $$\square $$

## Explicit formulas for the number of points

In this section, we derive explicit formulas for the number of points and identify the hypergeometric periods according to the action of the group of symmetries, matching the Picard–Fuchs equations computed in Sect. 2.

### Hypergeometric functions over finite fields

We begin by defining the finite-field analogue of the generalized hypergeometric function (defined in Sect. 2.4); we follow Beukers–Cohen–Mellit [3].

Let $q=p^r$ be a prime power. We use the convenient abbreviation

$$\begin{aligned} q^{\times }:=q-1. \end{aligned}$$

Let $\omega :{\mathbb {F}}_q^{\times } \rightarrow {\mathbb {C}}^\times$ be a generator of the character group on ${\mathbb {F}}_q^{\times }$. Let $\Theta :{\mathbb {F}}_q \rightarrow {\mathbb {C}}^\times$ be a nontrivial (additive) character, defined as follows: let $\zeta _p \in {\mathbb {C}}$ be a primitive *p*th root of unity, and define $\Theta (x)=\zeta _p^{{{\,\mathrm{Tr}\,}}_{{\mathbb {F}}_q|{\mathbb {F}}_p}(x)}$. For $m \in {\mathbb {Z}}$, we define the *Gauss sum*

3.1.1 $$\begin{aligned} g(m):=\sum _{x \in {\mathbb {F}}_q^{\times }} \omega (x)^m \Theta (x). \end{aligned}$$

We suppress the dependence on *q* in the notation, and note that *g*(*m*) depends only on $m \in {\mathbb {Z}}/q^{\times }{\mathbb {Z}}$ (and the choice of $\omega$ and $\zeta _p$).

#### Remark 3.1.2

Every generator of the character group on ${\mathbb {F}}_q^{\times }$ is of the form $\omega _k(x) :=\omega (x)^k$ for $k \in ({\mathbb {Z}}/q^{\times }{\mathbb {Z}})^\times$, and

$$\begin{aligned} \sum _{x \in {\mathbb {F}}_q^\times } \omega _k(x)^m \Theta (x) = g(km). \end{aligned}$$

Similarly, every additive character of ${\mathbb {F}}_q$ is of the form $\Theta _k(x) :=\zeta _p^{k{{\,\mathrm{Tr}\,}}(x)}$ for $k \in ({\mathbb {Z}}/p{\mathbb {Z}})^\times$, and

$$\begin{aligned} \sum _{x \in {\mathbb {F}}_q^\times } \omega (x)^m \Theta _k(x) = \omega (k)^{-m}g(m) \end{aligned}$$

(see, e.g., Berndt [1, Theorem 1.1.3]). Accordingly, we will see below that our definition of finite-field hypergeometric functions will not depend on these choices.

We will need four basic identities for Gauss sums.

#### Lemma 3.1.3

The following relations hold:

- $$g(0) = -1$$.
- $g(m) g(-m) = (-1)^m q$ for every $m \not \equiv 0 \pmod {q^{\times }}$, and in particular
  $$\begin{aligned} g\left( \tfrac{q^{\times }}{2}\right) ^2 = (-1)^{q^{\times }\,/2} q. \end{aligned}$$
- For every $N \mid q^{\times }$ with $N>0$, we have
  3.1.4 $$\begin{aligned} g(Nm) = -\omega (N)^{Nm} \prod _{j=0}^{N-1} \frac{g(m + jq^{\times }\,/N)}{g(jq^{\times }\,/N)}. \end{aligned}$$
- $$g(pm)=g(m)$$ for all $$m \in {\mathbb {Z}}$$.

#### Proof

For parts (a)–(c), see Cohen [12, Lemma 2.5.8, Proposition 2.5.9, Theorem 3.7.3]. For (d), we replace *x* by $x^p$ in the definition and use the fact that $\Theta (x^p)=\Theta (x)$ as it factors through the trace. $\square$

#### Remark 3.1.5

Lemma 3.1.3(c) is due originally to Hasse and Davenport, and is called the *Hasse–Davenport product relation*.

We now build our hypergeometric sums. Let $\varvec{\alpha }=\{\alpha _1,\dots ,\alpha _d\}$ and $\varvec{\beta }=\{\beta _1,\dots ,\beta _d\}$ be multisets of *d* rational numbers. Suppose that $\varvec{\alpha }$ and $\varvec{\beta }$ are *disjoint modulo *${\mathbb {Z}}$, i.e., $\alpha _i-\beta _j \not \in {\mathbb {Z}}$ for all $i,j=1,\dots ,d$.

Based on work of Greene [27], Katz [31, p. 258], but normalized following McCarthy [40, Definition 3.2] and Beukers–Cohen–Mellit [3, Definition 1.1], we make the following definition.

#### Definition 3.1.6

Suppose that

3.1.7 $$\begin{aligned} q^{\times }\alpha _i, q^{\times }\beta _i \in {\mathbb {Z}}\end{aligned}$$

for all $i=1,\dots ,d$. For $t \in {\mathbb {F}}_q^\times$, we define the *finite-field hypergeometric sum* by

3.1.8 $$\begin{aligned} H_q(\varvec{\alpha }, \varvec{\beta } \,|\, t) :=-\frac{1}{q^{\times }} \sum _{m=0}^{q-2} \omega ((-1)^dt)^m G(m+\varvec{\alpha }q^{\times },-m-\varvec{\beta }q^{\times }), \end{aligned}$$

where

3.1.9 $$\begin{aligned} G(m+\varvec{\alpha }q^{\times },-m-\varvec{\beta }q^{\times }) :=\prod _{i=1}^d \frac{g(m+ \alpha _iq^{\times })g(-m - \beta _iq^{\times })}{g(\alpha _i q^{\times })g(-\beta _i q^{\times })} \end{aligned}$$

for $$m \in {\mathbb {Z}}$$.

In this definition (and the related ones to follow), the sum $H_q(\varvec{\alpha },\varvec{\beta }\,|\,t)$ only depends on the classes in ${\mathbb {Q}}/{\mathbb {Z}}$ of the elements of $\varvec{\alpha }$ and $\varvec{\beta }$. Moreover, the sum is independent of the choice of characters $\omega$ and $\Theta$ by a straightforward application of Remark 3.1.2. Hypothesis (3.1.7) is unfortunately rather restrictive—but it is necessary for the definition to make sense as written. Fortunately, Beukers–Cohen–Mellit [3] provided an alternate definition that allows all, but finitely many *q* under a different hypothesis, as follows.

#### Definition 3.1.10

The *field of definition*$K_{\varvec{\alpha },\varvec{\beta }} \subset {\mathbb {C}}$ associated to $\varvec{\alpha },\varvec{\beta }$ is the field generated by the coefficients of the polynomials

3.1.11 $$\begin{aligned} \prod _{j=1}^d \left( x-e^{2\pi \sqrt{-1} \alpha _j}\right) \quad \text {and} \quad \prod _{j=1}^d \left( x-e^{2\pi \sqrt{-1} \beta _j}\right) . \end{aligned}$$

Visibly, the number field $K_{\varvec{\alpha },\varvec{\beta }}$ is an abelian extension of ${\mathbb {Q}}$.

Suppose that $\varvec{\alpha },\varvec{\beta }$ is defined over ${\mathbb {Q}}$, i.e., $K_{\varvec{\alpha },\varvec{\beta }}={\mathbb {Q}}$. Then by a straightforward verification, there exist $p_1, \ldots , p_r,q_1, \ldots , q_s \in {\mathbb {Z}}_{\ge 1}$ such that

3.1.12 $$\begin{aligned} \prod _{j=1}^d \frac{ (x-e^{2\pi \sqrt{-1} \alpha _j})}{(x-e^{2\pi \sqrt{-1} \beta _j})} = \frac{\prod _{j=1}^r x^{p_j} - 1}{\prod _{j=1}^s x^{q_j} - 1}. \end{aligned}$$

Recall we require the $\varvec{\alpha },\varvec{\beta }$ to be disjoint, which implies that the sets $\{p_1,\dots ,p_r\}$ and $\{q_1,\dots ,q_s\}$ are also disjoint.

Let $D(x) :=\gcd (\prod _{j=1}^r (x^{p_j} - 1), \prod _{j=1}^s (x^{q_j} - 1))$ and $M :=\bigl (\prod _{j=1}^r p_j^{p_j}\bigr ) \bigl (\prod _{j=1}^s q_j^{-q_j}\bigr )$. Let $\epsilon :=(-1)^{\sum _{j=1}^s q_j}$, and let $s(m) \in {\mathbb {Z}}_{\ge 0}$ be the multiplicity of the root $e^{2\pi \sqrt{-1} m / q^{\times }}$ in *D*(*x*). Finally, abbreviate

3.1.13 $$\begin{aligned} g(\varvec{p}m,-\varvec{q}m) :=g(p_1m) \cdots g(p_rm) g(-q_1m) \cdots g(-q_sm). \end{aligned}$$

For brevity, we say that *q* is *good* for $\varvec{\alpha },\varvec{\beta }$ if *q* is coprime to the least common denominator of $\varvec{\alpha } \cup \varvec{\beta }$.

#### Definition 3.1.14

Suppose that $\varvec{\alpha },\varvec{\beta }$ are defined over ${\mathbb {Q}}$ and *q* is good for $\varvec{\alpha },\varvec{\beta }$. For $t \in {\mathbb {F}}_q^\times$, define

3.1.15 $$\begin{aligned} H_q(\varvec{\alpha }, \varvec{\beta } \,|\, t) = \frac{(-1)^{r+s}}{1-q} \sum _{m=0}^{q-2} q^{-s(0) + s(m)} g(\varvec{p}m,-\varvec{q}m)\omega (\epsilon M^{-1}t)^m. \end{aligned}$$

Again, the hypergeometric sum $H_q(\varvec{\alpha },\varvec{\beta }\,|\,t)$ is independent of the choice of characters $\omega$ and $\Theta$. The independence on $\omega$ is just as with the previous definition, and in this case the independence from $\Theta$ comes from the fact that every root of unity has its conjugate, and so again any additional factors from changing additive characters cancel out. The apparently conflicting notation is justified by the following result, showing that Definition 3.1.14 is more general.

#### Proposition 3.1.16

(Beukers–Cohen–Mellit [3, Theorem 1.3]) Suppose that $\varvec{\alpha },\varvec{\beta }$ are defined over ${\mathbb {Q}}$ and that (3.1.7) holds. Then Definitions 3.1.6 and 3.1.14 agree.

### A hybrid sum

We will need a slightly more general hypothesis than allowed in the previous section. We do not pursue the most general case as it is rather combinatorially involved, poses some issues of algebraicity, and anyway is not needed here; see Beukers [2] for some work in this direction. Instead, we isolate a natural case, where the indices are not defined over ${\mathbb {Q}}$ but neither does (3.1.7) hold, which is sufficient for our purposes.

#### Definition 3.2.1

We say that *q* is *splittable* for $\varvec{\alpha },\varvec{\beta }$ if there exist partitions

3.2.2 $$\begin{aligned} \varvec{\alpha } = \varvec{\alpha }_0 \sqcup \varvec{\alpha }' \text { and } \varvec{\beta } = \varvec{\beta }_0 \sqcup \varvec{\beta }', \end{aligned}$$

where $\varvec{\alpha }_0,\varvec{\beta }_0$ are defined over ${\mathbb {Q}}$ and

$$\begin{aligned} q^{\times }\alpha _i',q^{\times }\beta _j' \in {\mathbb {Z}}\end{aligned}$$

for all $\alpha _i' \in \varvec{\alpha }'$ and all $\beta _j' \in \varvec{\beta }'$.

#### Example 3.2.3

If (3.1.7) holds, then *q* is splittable for $\varvec{\alpha },\varvec{\beta }$ taking $\varvec{\alpha }=\varvec{\alpha }'$ and $\varvec{\beta }=\varvec{\beta }'$ and $\varvec{\alpha }_0=\varvec{\beta }_0=\emptyset$. Likewise, if $\varvec{\alpha },\varvec{\beta }$ is defined over ${\mathbb {Q}}$, then *q* is splittable for $\varvec{\alpha },\varvec{\beta }$ for all *q*.

#### Example 3.2.4

A splittable case that arises for us (up to a Galois action) in Proposition 3.5.1 is as follows. Let $\varvec{\alpha } = \{\frac{1}{14}, \frac{9}{14}, \frac{11}{14}\}$ and $\varvec{\beta } = \{0, \frac{1}{4}, \frac{3}{4}\}$. We cannot use Definition 3.1.14 since $(x-e^{2\pi \sqrt{-1}/14})(x-e^{18\pi i/14})(x-e^{22\pi \sqrt{-1}/14}) \not \in {\mathbb {Q}}[x]$. When $q \equiv 1 \pmod {28}$, we may use Definition 3.1.6; otherwise we may not. However, when $q \equiv 1 \pmod {7}$ is odd, then *q* is splittable for $\varvec{\alpha },\varvec{\beta }$: We may take $\varvec{\alpha }_0=\emptyset$, $\varvec{\alpha }'=\varvec{\alpha }$ and $\varvec{\beta }_0=\varvec{\beta }$, $\varvec{\beta }'=\emptyset$.

It is now a bit notationally painful but otherwise straightforward to generalize the definition for splittable *q*, providing a uniform description in all cases we consider. Suppose that *q* is splittable for $\varvec{\alpha },\varvec{\beta }$. Let $\varvec{\alpha }_0$ be the union of all submultisets of $\varvec{\alpha }$ that are defined over ${\mathbb {Q}}$; then $\varvec{\alpha }_0$ is defined over ${\mathbb {Q}}$. Repeat this for $\varvec{\beta }_0$. Let $p_{1}, \ldots , p_{r}, q_{1}, \ldots , q_{s}$ be such that

$$\begin{aligned} \frac{\prod _{\alpha _{0j} \in \varvec{\alpha }_0} (x- e^{2\pi \sqrt{-1} \alpha _{0j}})}{\prod _{\beta _{0j} \in \varvec{\beta }_0} (x-e^{2\pi \sqrt{-1} \beta _{0j}})} = \frac{\prod _{j=1}^{r} (x^{p_{j}} -1)}{\prod _{j=1}^{s} (x^{q_{j}}-1)}. \end{aligned}$$

As before, let

$$\begin{aligned} D(x) :=\gcd (\textstyle {\prod }_{j=1}^{r} x^{p_{j}} - 1, \textstyle {\prod }_{j=1}^{s} x^{q_{j}} - 1) \quad \text {and} \quad M :=\frac{\textstyle {\prod }_{j=1}^{r} p_{j}^{p_{j}}}{\textstyle {\prod }_{j=1}^{s} q_{j}^{q_{j}}} \end{aligned}$$

and let *s*(*m*) be the multiplicity of the root $e^{2\pi \sqrt{-1} m / q^{\times }}$ in *D*(*x*). Finally, let $\delta :=\deg D(x)$. We again abbreviate

3.2.5 $$\begin{aligned} g(\varvec{p}m,-\varvec{q}m) :=\prod _{i=1}^{r} g(p_{i}m) \prod _{i=1}^{s} g(-q_{i}m) \end{aligned}$$

for $$m \in {\mathbb {Z}}$$ and

3.2.6 $$\begin{aligned} G(m+\varvec{\alpha }'q^{\times },-m-\varvec{\beta }'q^{\times }) :=\prod _{\alpha _i' \in \varvec{\alpha }'}\frac{g(m+ \alpha _i'q^{\times })}{g(\alpha _i q^{\times })} \prod _{\beta _i' \in \varvec{\beta }'}\frac{g(-m-\beta _i'q^{\times })}{g(-\beta _i q^{\times })}. \end{aligned}$$

#### Definition 3.2.7

Suppose that *q* is good and splittable for $\varvec{\alpha },\varvec{\beta }$. For $t \in {\mathbb {F}}_q^\times$, with the notation above we define the *finite-field hypergeometric sum*

$$\begin{aligned} H_q(\varvec{\alpha }, \varvec{\beta } \, | \, t):= & {} \frac{(-1)^{r+s}}{1-q} \sum _{m=0}^{q-2} q^{- s(0) + s(m)} G(m+\varvec{\alpha }'q^{\times },-m-\varvec{\beta }'q^{\times })\\&\cdot g(\varvec{p}m,-\varvec{q}m)\omega ((-1)^{d+\delta } Mt)^m. \end{aligned}$$

The following proposition then shows that our definition encompasses the previous ones.

#### Proposition 3.2.8

Suppose that *q* is good and splittable for $\varvec{\alpha },\varvec{\beta }$. Then the following statements hold.

- The hypergeometric sum $H_q(\varvec{\alpha },\varvec{\beta }\,|\,t)$ in Definition 3.2.7 is independent of the choice of characters $\omega$ and $\Theta$.
- If $\alpha _i q^{\times }, \beta _i q^{\times }\in {\mathbb {Z}}$ for all $i=1,\dots ,d$, then Definitions 3.1.6 and 3.2.7 agree.
- If $\varvec{\alpha },\varvec{\beta }$ are defined over ${\mathbb {Q}}$, then Definitions 3.1.14 and 3.2.7 agree.

#### Proof

Part (c) follows directly from $\varvec{\alpha }_0 = \varvec{\alpha }$ and $\varvec{\beta }_0 = \varvec{\beta }$ (and $\varvec{\alpha }' = \varvec{\beta }' = \emptyset$), so the definitions in fact coincide. Part (a) follows directly from the independence from $\Theta$ and $\omega$ of each part of the hybrid sum.

Part (b) follows by the same argument (due to Beukers–Cohen–Mellit) as in Proposition 3.1.16; for completeness, we give a proof in Lemma B.1.1. $\square$

Suppose that *q* is good and splittable for $\varvec{\alpha },\varvec{\beta }$ and let $t \in {\mathbb {F}}_q^\times$. Then by construction $H_q(\varvec{\alpha },\varvec{\beta }\,|\,t) \in {\mathbb {Q}}(\zeta _{q^{\times }},\zeta _p)$. Since $\gcd (p,q^{\times })=1$, we have

$$\begin{aligned} {{\,\mathrm{Gal}\,}}({\mathbb {Q}}(\zeta _{q^{\times }},\zeta _p)\,|\,{\mathbb {Q}}) \simeq {{\,\mathrm{Gal}\,}}({\mathbb {Q}}(\zeta _{q^{\times }})\,|\,{\mathbb {Q}}) \times {{\,\mathrm{Gal}\,}}({\mathbb {Q}}(\zeta _{p})\,|\,{\mathbb {Q}}). \end{aligned}$$

We now descend the hypergeometric sum to its field of definition, in two steps.

#### Lemma 3.2.9

We have $$H_q(\varvec{\alpha },\varvec{\beta }\,|\,t) \in {\mathbb {Q}}(\zeta _{q^{\times }})$$.

#### Proof

The action of ${{\,\mathrm{Gal}\,}}({\mathbb {Q}}(\zeta _p)\,|\,{\mathbb {Q}}) \simeq ({\mathbb {Z}}/p{\mathbb {Z}})^\times$ by $\zeta _p \mapsto \zeta _p^k$ changes only the additive character $\Theta$. By Proposition 3.2.8, the sum is independent of this choice, so it descends by Galois theory. $\square$

The group ${{\,\mathrm{Gal}\,}}({\mathbb {Q}}(\zeta _{q^{\times }})\,|\,{\mathbb {Q}}) \simeq ({\mathbb {Z}}/q^{\times }{\mathbb {Z}})^\times$ by $\sigma _k(\zeta _{q^{\times }})=\zeta _({q^{\times }})^k$ for $k \in ({\mathbb {Z}}/q^{\times }{\mathbb {Z}})^\times$ acts on the finite-field hypergeometric sums as follows.

#### Lemma 3.2.10

The following statements hold.

- Let $k \in {\mathbb {Z}}$ be coprime to $q^{\times }$. Then $\sigma _k(H_q(\varvec{\alpha },\varvec{\beta }\,|\, t)) = H_q(k\varvec{\alpha },k\varvec{\beta }\,|\,t)$.
- We have $$H_q(\varvec{\alpha },\varvec{\beta }\,|\, t) \in K_{\varvec{\alpha },\varvec{\beta }}$$.
- We have $$H_q(p\varvec{\alpha },p\varvec{\beta }\,|\, t) = H_q(\varvec{\alpha },\varvec{\beta }\,|\, t^p)$$.

#### Proof

To prove (a), note $\sigma _k(\omega (x))=\omega ^k(x)$ since $\omega$ takes values in $\mu _{q^{\times }}$; therefore, $\sigma _k(g(m))=g(km)$, and we have both

$$\begin{aligned} \sigma _k(g(\varvec{p} m, -\varvec{q} m)) = g(\varvec{p} km, -\varvec{q} km) \end{aligned}$$

and

$$\begin{aligned} \sigma _k(G(m+\varvec{\alpha }'q^{\times },-m-\varvec{\beta }'q^{\times })) = G(km+k\varvec{\alpha }'q^{\times },-km-k\varvec{\beta }'q^{\times })). \end{aligned}$$

We have $s(km)=s(m)$ since $D(x) \in {\mathbb {Q}}[x]$. Moreover, $k\varvec{\alpha }_0=\varvec{\alpha }_0$ and the same with $\varvec{\beta }$, so the values $p_{i},q_{i}$ remain the same when computed for $k\varvec{\alpha },k\varvec{\beta }$. Now, plug these into the definition of $H_q(\varvec{\alpha },\varvec{\beta }\,|\,t)$ and just reindex the sum by $km \leftarrow m$ to obtain the result.

Part (b) follows from part (a): The field of definition $K_{\varvec{\alpha },\varvec{\beta }}$ is precisely the fixed field under the subgroup of $k \in ({\mathbb {Z}}/q^{\times }{\mathbb {Z}})^\times$ such that $k\varvec{\alpha },k\varvec{\beta }$ are equivalent to $\varvec{\alpha },\varvec{\beta }$ as multisets in ${\mathbb {Q}}/{\mathbb {Z}}$.

Finally, part (c). Starting with the left-hand side, we reindex $m \leftarrow pm$ then substitute using Lemma 3.1.3(d)’s implication that $g(pm)=g(m)$ to get

$$G(pm+p\varvec{\alpha }'q^{\times },-pm-p\varvec{\beta }'q^{\times })=G(m+\varvec{\alpha }'q^{\times },-m-\varvec{\beta }'q^{\times })$$ and $$g(\varvec{p}(pm),\varvec{q}(pm))=g(\varvec{p}m,\varvec{q}m)$$,

noting that the quantities $\varvec{p}$ and $\varvec{q}$ do not change, as $p\varvec{\alpha }_0 = \varvec{\alpha }_0$ and $p\varvec{\beta }_0=\varvec{\beta }_0$ modulo ${\mathbb {Z}}$ (as they are defined over ${\mathbb {Q}}$). Noting that $((-1)^{d+\delta } M)^p = (-1)^{d+\delta }M \in {\mathbb {F}}_p \subseteq {\mathbb {F}}_q$, we then obtain the result. $\square$

Before concluding this primer on finite-field hypergeometric functions, we combine the Gauss sum identities and our hybrid definition to expand one essential example; this gives a flavor of what is to come. First, we prove a new identity.

#### Lemma 3.2.11

We have the following identity of Gauss sums:

$$\begin{aligned} g\left( \tfrac{q^{\times }}{14}\right) g\left( \tfrac{9q^{\times }}{14}\right) g\left( \tfrac{11q^{\times }}{14}\right) = g\left( \tfrac{q^{\times }}{2}\right) ^3 = \left( -1\right) ^{q^{\times }\,/2}q g\left( \tfrac{q^{\times }}{2}\right) . \end{aligned}$$

#### Proof

Since *q* is odd, we use the Hasse–Davenport product relation (Lemma 3.1.3(c)) for $N=2$ and $m = \tfrac{q^{\times }}{14}, \tfrac{9q^{\times }}{14},\tfrac{11q^{\times }}{14}$, solving for $g(\tfrac{q^{\times }}{14}), g(\tfrac{9q^{\times }}{14}),g(\tfrac{11q^{\times }}{14})$, respectively, to find:

$$\begin{aligned} g\left( \tfrac{q^{\times }}{14}\right)&= \frac{g\left( \tfrac{q^{\times }}{7}\right) , g\left( \tfrac{q^{\times }}{2}\right) }{g\left( \tfrac{11q^{\times }}{7}\right) } \omega \left( 2\right) ^{-q^{\times }\,/7} \\ g\left( \tfrac{9q^{\times }}{14}\right)&= \frac{g\left( \tfrac{9q^{\times }}{7}\right) , g\left( \tfrac{q^{\times }}{2}\right) }{g\left( \tfrac{q^{\times }}{7}\right) } \omega \left( 2\right) ^{-9q^{\times }\,/7} \\ g\left( \tfrac{11q^{\times }}{14}\right)&= \frac{g\left( \tfrac{11q^{\times }}{7}\right) . g\left( \tfrac{q^{\times }}{2}\right) }{g\left( \tfrac{9q^{\times }}{7}\right) } \omega \left( 2\right) ^{-11q^{\times }\,/7}. \end{aligned}$$

Multiply all of these together, divide by $g(\tfrac{q^{\times }}{2})$, and cancel to obtain

3.2.12 $$\begin{aligned} \begin{aligned} \frac{g\left( \tfrac{q^{\times }}{14}\right) g\left( \tfrac{9q^{\times }}{14}\right) g\left( \tfrac{11q^{\times }}{14}\right) }{g\left( \tfrac{q^{\times }}{2}\right) }&= g\left( \tfrac{q^{\times }}{2}\right) ^2 \omega \left( 2\right) ^{-3q^{\times }} = \left( -1\right) ^{q^{\times }\,/2} q \end{aligned} \end{aligned}$$

applying Lemma 3.1.3(b) in the last step. $\square$

Next, we consider our example.

#### Example 3.2.13

Going back to Example 3.2.4, in the case where $q \equiv 1 \pmod 7$ and *q* odd, we have $\varvec{\alpha } = \{\frac{1}{14}, \frac{9}{14}, \frac{11}{14}\}$ and $\varvec{\beta } = \{0, \frac{1}{4}, \frac{3}{4}\}$. Then $\varvec{\alpha }_0=\emptyset$ and $\varvec{\beta }_0=\varvec{\beta }$. Thus,

$$\begin{aligned} \frac{\prod _{\alpha _{0j} \in \varvec{\alpha }_0} (x- e^{2\pi \sqrt{-1} \alpha _{0j}})}{\prod _{\beta _{0j} \in \varvec{\beta }_0} (x-e^{2\pi \sqrt{-1} \beta _{0j}})} = \frac{1}{(x-1)(x^2+1)} = \frac{(x^2-1)}{(x-1)(x^4-1)}. \end{aligned}$$

Thus, $D(x) = x^2-1$ and $M = 4^3$; and $s(m) = 1$ if $m=0, \tfrac{q^{\times }}{2}$ and $s(m)=0$ otherwise. Therefore, Definition 3.2.7 and simplification using Lemma 3.1.3(a)–(b) give

$$\begin{aligned} \begin{aligned}&H_q\left( \tfrac{1}{14}, \tfrac{9}{14}, \tfrac{11}{14}; 0,\tfrac{1}{4},\tfrac{3}{4} \,|\, t\right) \\&\quad = \frac{-1}{1-q} \sum _{m=0}^{q-2} q^{s\left( m\right) - 1} \frac{g\left( m+ \frac{1}{14}q^{\times }\right) g\left( m+ \frac{9}{14}q^{\times }\right) g\left( m+ \frac{11}{14}q^{\times }\right) }{g\left( \frac{1}{14} q^{\times }\right) g\left( \frac{9}{14} q^{\times }\right) g\left( \frac{11}{14} q^{\times }\right) } \\&\qquad \cdot g\left( 2m\right) g\left( -m\right) g\left( -4m\right) \omega \left( -4^3t\right) ^m. \end{aligned} \end{aligned}$$

When $m=0$, the summand is just $(-1)(-1)^3/(1-q)=1/q^{\times }$. When $m=\tfrac{q^{\times }}{2}$, applying Lemma 3.2.11 we obtain

$$\begin{aligned}&\frac{-g\left( \frac{4q^{\times }}{7}\right) g\left( \frac{q^{\times }}{7}\right) g\left( \frac{2q^{\times }}{7}\right) }{q^{\times }g\left( \frac{1}{14} q^{\times }\right) g\left( \frac{9}{14} q^{\times }\right) g\left( \frac{11}{14} q^{\times }\right) } g\left( \tfrac{q^{\times }}{2}\right) \omega \left( -4^3t\right) ^{q^{\times }\,/2} \\&\quad = \frac{-1}{qq^{\times }} g\left( \tfrac{q^{\times }}{7}\right) g\left( \tfrac{2q^{\times }}{7}\right) g\left( \tfrac{4q^{\times }}{7}\right) \omega \left( t\right) ^{q^{\times }\,/2}. \end{aligned}$$

Therefore,

3.2.14 $$\begin{aligned} \begin{aligned}&H_q\left( \tfrac{1}{14}, \tfrac{9}{14}, \tfrac{11}{14}; 0,\tfrac{1}{4},\tfrac{3}{4} \,|\, t\right) \\&\quad = \frac{1}{q^{\times }} -\frac{1}{qq^{\times }} g\left( \tfrac{q^{\times }}{7}\right) g\left( \tfrac{2q^{\times }}{7}\right) g\left( \tfrac{4q^{\times }}{7}\right) \omega \left( t\right) ^{q^{\times }\,/2}\\&\qquad +\,\frac{1}{qq^{\times }} \sum _{\begin{array}{c} m=1 \\ m \ne q^{\times }\,/2 \end{array}}^{q-2} \frac{g\left( m+ \frac{1}{14}q^{\times }\right) g\left( m+ \frac{9}{14}q^{\times }\right) g\left( m+ \frac{11}{14}q^{\times }\right) }{g\left( \frac{1}{14} q^{\times }\right) g\left( \frac{9}{14} q^{\times }\right) g\left( \frac{11}{14} q^{\times }\right) } \\&\qquad \cdot \, g\left( 2m\right) g\left( -m\right) g\left( -4m\right) \omega \left( -4^3t\right) ^m. \end{aligned} \end{aligned}$$

### Counting points

Following the work of Delsarte [16] and Furtado Gomida [22], Koblitz [37] gave a formula for the number of points on monomial deformations of diagonal hypersurfaces (going back to Weil [50]). In this subsection, we outline their approach for creating closed formulas that compute the number of points for hypersurfaces in projective space in terms of Gauss sums.

Let $X \subseteq {\mathbb {P}}^n$ be the projective hypersurface over ${\mathbb {F}}_q$ defined by the vanishing of the nonzero polynomial

$$\begin{aligned} \sum _{i=1}^r a_i x_0^{\nu _{i0}}\cdots x_n^{\nu _{in}} \in {\mathbb {F}}_q[x_0,\dots ,x_n] \end{aligned}$$

so that $a_i \in {\mathbb {F}}_q$ and $\nu _{ij} \in {\mathbb {Z}}_{\ge 0}$ for $i=1,\dots ,r$ and $j=0,\dots ,n$. Suppose that $q^{\times }:=q-1$ does not divide any of the $\nu _{ij}$. Let *U* be the intersection of *X* with the torus ${\mathbb {G}}_m^{n+1}/{\mathbb {G}}_m \subseteq {\mathbb {P}}^n$, so that the points of *U* are the points of *X* with all nonzero coordinates.

Let $S$ be the set of $s = (s_1,\ldots , s_r) \in ({\mathbb {Z}}/q^{\times }{\mathbb {Z}})^r$ such that the following condition holds:

3.3.1 $$\begin{aligned} \textstyle {\sum _{i=1}^r} s_i\equiv 0~({\text {mod}}~{q^{\times }}) \quad \text {and}\quad \sum _{i=1}^r \nu _{ij}s_i\equiv 0~({\text {mod}}~{q^{\times }}) \quad \text { for all } j=1,\dots ,n. \end{aligned}$$

Let $\mu _q^{\times }$ be the group of $q^{\times }$-th roots of unity. Any element $s = (s_1,\ldots , s_r) \in S$ corresponds to a multiplicative character

$$\begin{aligned} \chi _{s} :\mu _({q^{\times }})^r&\rightarrow {\mathbb {C}}^\times \\ \chi _{s}(x_1, \ldots , x_r)&= \omega ( \Pi _{i=1}^r x_i^{s_i}). \end{aligned}$$

Given $s \in S$, we define

3.3.2 $$\begin{aligned} c_s :=\frac{({q^{\times }})^{n-r+1}}{q}\prod _{i=1}^r g(s_i) \end{aligned}$$

if $$s \ne 0$$ and

$$\begin{aligned} c_{0} :=({q^{\times }})^{n-r+1}\frac{({q^{\times }})^{r-1}-(-1)^{r-1}}{q}. \end{aligned}$$

With this notation, we have the following result of Koblitz, rewritten in terms of Gauss sums so that we can apply it in our context.

#### Theorem 3.3.3

(Koblitz) We have

3.3.4 $$\begin{aligned} \# U({\mathbb {F}}_q)=\sum _{s\in S}\omega (a)^{-s}c_{s}, \end{aligned}$$

where $$\omega (a)^{-s} :=\omega (a_1^{-s_1}\cdots a_r^{-s_r})$$.

#### Proof

We unpack and repack a bit of notation. Koblitz [37, Theorem 1] proves that

$$\begin{aligned} \# U({\mathbb {F}}_q)=\sum _{s}\omega (a)^{-s}c_{s}', \end{aligned}$$

where the sum is over all characters of $\mu _({q^{\times }})^r / \Delta$ where $\Delta$ is the diagonal—this set is in natural bijection with the set *S*—and where for $s \ne 0$

$$\begin{aligned} c_s' = -\frac{1}{q} ({q^{\times }})^{n-r+1} J(s_1, \ldots , s_r), \end{aligned}$$

where $J(s_1,\ldots , s_r)$ is the Jacobi sum and where $c_0'=c_0$ as in (3.3.2). It only remains to show that $c'_s=c_s$ for $s \ne 0$. If $s_i \ne 0$ for all *i*, then [37, (2.5)]

$$\begin{aligned} J(s_1,\ldots , s_r) = \frac{g(s_1) \cdots g(s_r)}{g(s_1+\ldots +s_r)} = - g(s_1) \cdots g(s_r), \end{aligned}$$

so $c_s' = c_s$ by definition. If $r>1$ and $s_i =0$ for some *i*, then [37, below (2.5)]

$$\begin{aligned} J(s_1,\ldots , s_r) = -J(s_1, \ldots , s_{i-1},s_{i+1}, \ldots , s_r), \end{aligned}$$

so iterating and using Lemma 3.1.3(a),

3.3.5 $$\begin{aligned} J(s_1,\ldots , s_r) = -\prod _{\begin{array}{c} i=1,\dots ,r \\ s_i = 0 \end{array}} (-1) \prod _{\begin{array}{c} i=1,\dots ,r \\ s_i \ne 0 \end{array}} g(s_i) = -\prod _{\begin{array}{c} i=1,\dots ,r \\ s_i = 0 \end{array}} g(0) \prod _{\begin{array}{c} i=1,\dots ,r \\ s_i \ne 0 \end{array}} g(s_i) = - \prod _{i=1}^r g(s_i). \end{aligned}$$

$$\square $$

In the remaining sections, we apply the preceding formulas to each of our five pencils.

### The Dwork pencil ${\mathsf {F}}_4$

In this subsection, we will give a closed formula in terms of finite field hypergeometric sums for the number of points in a given member of the Dwork family. Throughout this section, we suppose that *q* is odd.

#### Proposition 3.4.1

For $\psi \in {\mathbb {F}}_q^\times$, the following statements hold.

- If $$q\equiv 3\pmod 4$$, then
  $$\begin{aligned} \#X_{{\mathsf {F}}_4,\psi }\left( {\mathbb {F}}_q\right) =q^2+q+1+H_q\left( \tfrac{1}{4},\tfrac{1}{2},\tfrac{3}{4};0,0,0\,|\,\psi ^{-4}\right) -3qH_q\left( \tfrac{1}{4},\tfrac{3}{4};0, \tfrac{1}{2}\,|\,\psi ^{-4}\right) . \end{aligned}$$
- If $$q\equiv 1\pmod {4}$$, then
  $$\begin{aligned} \#X_{{\mathsf {F}}_4,\psi }\left( {\mathbb {F}}_q\right)&= q^2+q+1+H_q\left( \tfrac{1}{4},\tfrac{1}{2},\tfrac{3}{4};0,0,0\,|\,\psi ^{-4}\right) +3qH_q\left( \tfrac{1}{4},\tfrac{3}{4};0, \tfrac{1}{2}\,|\,\psi ^{-4}\right) \\&\qquad +12\left( -1\right) ^{\left( q-1\right) /4}qH_q\left( \tfrac{1}{2};0\,|\,\psi ^{-4}\right) . \end{aligned}$$

Proposition 3.4.1 has several equivalent formulations and has seen many proofs: See Sect. 1.5 in the introduction for further references. We present another proof for completeness and to illustrate the method we will apply to all five families in this well-studied case.

#### Remark 3.4.2

Quite beautifully, the point counts in Proposition 3.4.1 in terms of finite-field hypergeometric sums match (up to twisting factors) the indices with multiplicity in the Picard–Fuchs equations computed in Proposition 2.5.2. Although we are not able to use this matching directly, it guides the decomposition of the sums by means of lemmas that can be proven in a technical, but direct manner.

We prove Proposition 3.4.1 in four steps:

1. We compute the relevant characters and cluster them.
2. We use Theorem 3.3.3 to count points where no coordinate is zero and rewrite the sums into hypergeometric functions.
3. We count points where at least one coordinate is zero.
4. We combine steps 2 and 3 to finally prove Proposition 3.4.1.

The calculations are somewhat involved, but we know how to cluster the characters in step 1 and which hypergeometric functions we need to isolate for step 2: Indeed, the parameters of the finite-field hypergeometric sums are given by the calculation of the Picard–Fuchs equations for the Dwork pencil given by Proposition 2.5.2.

#### Step 1: Computing and clustering the characters

In order to use Theorem 3.3.3, we must compute the subset $S\subset ({\mathbb {Z}}/q^{\times }{\mathbb {Z}})^r$ given by the constraints in (3.3.1). This is equivalent to solving the system of congruences:

3.4.3 $$\begin{aligned} \begin{pmatrix} 4&{}\quad 0&{}\quad 0&{}\quad 0&{}\quad 1\\ 0&{}\quad 4&{}\quad 0&{}\quad 0&{}\quad 1\\ 0&{}\quad 0&{}\quad 4&{}\quad 0&{}\quad 1\\ 0&{}\quad 0&{}\quad 0&{}\quad 4&{}\quad 1\\ 1&{}\quad 1&{}\quad 1&{}\quad 1&{}\quad 1 \end{pmatrix} \begin{pmatrix} s_1 \\ s_2 \\ s_3 \\ s_4 \\ s_5 \end{pmatrix} \equiv 0 \pmod {q^{\times }}. \end{aligned}$$

If $q\equiv 3\pmod 4$, then by linear algebra over ${\mathbb {Z}}$ we obtain

$$\begin{aligned} S= \left\{ (1,1,1,1,-4)k_1 + \tfrac{q^{\times }}{2}(0,0,1,-1,0) k_2 + \tfrac{q^{\times }}{2} (0,1,0,-1,0)k_3:k_i \in {\mathbb {Z}}/q^{\times }{\mathbb {Z}}\right\} . \end{aligned}$$

These solutions can be clustered in an analogous way as done in Sect. 2.5:

(i) $$S_1 :=\{ k(1,1,1,1,-4) : k \in {\mathbb {Z}}/ q^{\times }{\mathbb {Z}}\}$$,
(ii) $$S_2 :=\{ k(1,1,1,1,-4) + \tfrac{q^{\times }}{2} (0,1,1,0,0) : k \in {\mathbb {Z}}/q^{\times }{\mathbb {Z}}\}$$,
(iii) $$S_3 :=\{ k(1,1,1,1,-4) + \tfrac{q^{\times }}{2} (0,1,0,1,0) : k \in {\mathbb {Z}}/q^{\times }{\mathbb {Z}}\}$$, and
(iv) $$S_4 :=\{ k(1,1,1,1,-4) + \tfrac{q^{\times }}{2} (0,0,1,1,0) : k \in {\mathbb {Z}}/q^{\times }{\mathbb {Z}}\}$$.

The last three (ii)–(iv) all behave in the same way, due to the evident symmetry.

If instead $q\equiv 1\pmod 4$, then

$$\begin{aligned} S= \left\{ (1,1,1,1,-4)k_1 + \tfrac{q^{\times }}{4}(0,1,0,-1,0)k_2 + \tfrac{q^{\times }}{4} (0,0,1,-1,0)k_3 : k_i \in {\mathbb {Z}}/q^{\times }{\mathbb {Z}}\right\} ; \end{aligned}$$

we cluster again, getting the four clusters above but now together with twelve new clusters:

(v) three sets of the form $S_5 :=\{k(1,1,1,1,-4) + \tfrac{q^{\times }}{4}(0,1,2,1,0) : k \in {\mathbb {Z}}/q^{\times }{\mathbb {Z}}\}$,
(vi) three sets of the form $S_6 :=\{k(1,1,1,1,-4) - \tfrac{q^{\times }}{4}(0,1,2,1,0) : k \in {\mathbb {Z}}/q^{\times }{\mathbb {Z}}\}$, and
(vii) six sets of the form $S_7 :=\{k(1,1,1,1,-4) + \tfrac{q^{\times }}{4}(0,0,1,3,0) : k \in {\mathbb {Z}}/q^{\times }{\mathbb {Z}}\}$,

where the number of sets is given by the number of distinct permutations of the middle three coordinates.

#### Step 2: Counting points on the open subset with nonzero coordinates

We now give a formula for $\#U_{{\mathsf {F}}_4, \psi }({\mathbb {F}}_q)$ for the number of points, applying Theorem 3.3.3.

We go through each cluster $S_i$, linking each to a hypergeometric function.

##### Lemma 3.4.4

For all odd *q*,

3.4.5 $$\begin{aligned} \sum _{s \in S_1} \omega (a)^{-s} c_s= q^2-3q+3 + H_q\left( \tfrac{1}{4}, \tfrac{1}{2}, \tfrac{3}{4}; 0,0,0 \,|\, \psi ^{-4}\right) . \end{aligned}$$

##### Proof

By Definition 3.1.14,

$$\begin{aligned} H_q\left( \tfrac{1}{4}, \tfrac{1}{2}, \tfrac{3}{4}; 0,0,0 \,|\, \psi ^{-4}\right)&= \frac{1}{q^{\times }} \sum _{m=0}^{q-2} q^{-s(0) + s(m)} g(4m) g(-m)^4 \omega (4\psi )^{-4m} \\&= \frac{-1}{q^{\times }} + \frac{-q^{-1} g\left( \tfrac{q^{\times }}{2}\right) ^4}{q^{\times }} \\&\quad + \frac{1}{q^{\times }}\sum _{\begin{array}{c} m=1 \\ m\ne q^{\times }\,/2 \end{array}}^{q-2} q^{-1} g(4m) g(-m)^4 \omega (4\psi )^{-4m} \\&=- \frac{1}{q^{\times }} - \frac{g\left( \tfrac{q^{\times }}{2}\right) ^4}{qq^{\times }} + \frac{1}{qq^{\times }}\sum _{\begin{array}{c} k=1 \\ k\ne q^{\times }\,/2 \end{array}}^{q-2} g(-4k) g(k)^4 \omega (4\psi )^{4k} \end{aligned}$$

the latter by substituting $k=-m$. Now, we expand to match terms:

3.4.6 $$\begin{aligned} \sum _{s \in S_1} \omega (a)^{-s} c_s&= c_{(0,0,0,0,0)} + c_{(q^{\times }\,/2)(1,1,1,1,0)} + \sum _{\begin{array}{c} k=1 \\ k\ne q^{\times }\,/2 \end{array}}^{q-2} \omega (-4\psi )^{4k} c_{(k,k,k,k,-4k)} \nonumber \\&= \frac{({q^{\times }})^4 - (-1)^4}{qq^{\times }} - \frac{1}{qq^{\times }} g\left( \tfrac{q^{\times }}{2}\right) ^4 + \frac{1}{qq^{\times }}\sum _{\begin{array}{c} k=1 \\ k\ne q^{\times }\,/2 \end{array}}^{q-2} \omega (4\psi )^{4k} g(k)^4 g(-4k) \nonumber \\&= \frac{q^3-4q^2+6q-4}{q^{\times }} - \frac{g\left( \tfrac{q^{\times }}{2}\right) ^4}{qq^{\times }} + \frac{1}{qq^{\times }} \sum _{k=1, k\ne \tfrac{q^{\times }}{2}}^{q-2} \omega (4\psi )^{4k} g(k)^4 g(-4k) \nonumber \\&= q^2-3q+3 + H_q\left( \tfrac{1}{4}, \tfrac{1}{2}, \tfrac{3}{4}; 0,0,0 \,|\, \psi ^{-4}\right) . \end{aligned}$$

$$\square $$

##### Lemma 3.4.7

For $$i = 2, 3, 4$$,

3.4.8 $$\begin{aligned} \sum _{s \in S_i} \omega (a)^{-s} c_s = (-1)^{q^{\times }\,/2}\bigl (2 + qH_q(\tfrac{1}{4}, \tfrac{3}{4}; 0, \tfrac{1}{2} \,|\, \psi ^{-4})\bigr ). \end{aligned}$$

##### Proof

By definition,

3.4.9 $$\begin{aligned} \begin{aligned} H_q\left( \tfrac{1}{4}, \tfrac{3}{4}; 0, \tfrac{1}{2} \,|\, \psi ^{-4}\right)&= \frac{1}{q^{\times }} \sum _{m=0}^{q-2} q^{-s(0) + s(m)}g(4m)g(-2m)^2 \omega (2\psi )^{-4m} \\&= \frac{-2}{q^{\times }} + \frac{1}{q^{\times }} \sum _{\begin{array}{c} m=1 \\ m \ne q^{\times }\,/2 \end{array}}^{q-2} q^{-1}g(4m)g(-2m)^2 \omega (2\psi )^{-4m} \end{aligned} \end{aligned}$$

using $s(m) = 1$ if $m = 0,\tfrac{q^{\times }}{2}$ and $s(m)=0$ otherwise.

By symmetry,

$$\begin{aligned} \sum _{s \in S_2} \omega (a)^{-s} c_s = \sum _{s \in S_3} \omega (a)^{-s} c_s = \sum _{s \in S_4} \omega (a)^{-s} c_s. \end{aligned}$$

So we only need to consider $i=2$. Then:

3.4.10 $$\begin{aligned} \begin{aligned} \sum _{s \in S_2} \omega \left( a\right) ^{-s} c_s&= c_{\left( q^{\times }\,/2\right) \left( 0,1,1,0,0\right) } + c_{\left( q^{\times }\,/2\right) \left( 1,0,0,1,0\right) } \\&\quad +\, \sum _{\begin{array}{c} k=1 \\ k\ne q^{\times }\,/2 \end{array}}^{q-2} \omega \left( -4\psi \right) ^{4k} c_{\left( k\left( 1,1,1,1,-4\right) + \left( q^{\times }\,/2\right) \left( 0,1,1,0,0\right) \right) } \\&= -2\frac{g\left( \tfrac{q^{\times }}{2}\right) ^2}{qq^{\times }} + \frac{1}{qq^{\times }} \sum _{\begin{array}{c} k=1 \\ k\ne q^{\times }\,/2 \end{array}}^{q-2} \omega \left( -4\psi \right) ^{4k}g\left( k\right) ^2g\left( k+\tfrac{q^{\times }}{2}\right) ^2g\left( -4k\right) . \end{aligned} \end{aligned}$$

Next, we use the Hasse–Davenport product relation (Lemma 3.1.3(c)) with $N=2 \mid q^{\times }$ to get

$$\begin{aligned} g(2k) = -\omega (2)^{2k} \frac{g(k)}{g(0)} \frac{g\left( k+\tfrac{q^{\times }}{2}\right) }{g\left( \frac{q^{\times }}{2}\right) }, \end{aligned}$$

which rearranges using $g(0)=-1$ to

3.4.11 $$\begin{aligned} g(k)g\left( k+\tfrac{q^{\times }}{2}\right) = \omega (2)^{-2k} g(2k) g\left( \tfrac{q^{\times }}{2}\right) . \end{aligned}$$

Using Lemma 3.1.3(b) gives $g(\tfrac{q^{\times }}{2})^2 = (-1)^{q^{\times }\,/2} q$; substituting this and (3.4.11) into (3.4.10) simplifies to

$$\begin{aligned} \begin{aligned} \sum _{s \in S_2} \omega (a)^{-s} c_s&= -2\frac{(-1)^{q^{\times }\,/2}}{q^{\times }} + \frac{1}{qq^{\times }} \sum _{\begin{array}{c} k=1 \\ k\ne q^{\times }\,/2 \end{array}}^{q-2} \omega (-4\psi )^{4k}(\omega (2)^{-2k}g(2k) g(\tfrac{q^{\times }}{2}))^2 g(-4k) \\&= -2\frac{(-1)^{q^{\times }\,/2}}{q^{\times }} + \frac{1}{q^{\times }} \sum _{\begin{array}{c} k=1 \\ k\ne q^{\times }\,/2 \end{array}}^{q-2} (-1)^{q^{\times }\,/2}\omega (-2\psi )^{4k}g(2k)^2 g(-4k) \\&= (-1)^{q^{\times }\,/2}\left( -\frac{2}{q^{\times }} + \frac{1}{q^{\times }} \sum _{\begin{array}{c} k=1 \\ k\ne q^{\times }\,/2 \end{array}}^{q-2} \omega (-2\psi )^{4k}g(2k)^2 g(-4k)\right) . \end{aligned} \end{aligned}$$

Looking back at (3.4.9), we rearrange and insert a factor *q* to find the hypergeometric sum:

$$\begin{aligned} \begin{aligned} (-1)^{q^{\times }\,/2} \sum _{s \in S_2} \omega (a)^{-s} c_s&= \frac{2q-2}{q^{\times }} - \frac{2q}{q^{\times }} + \frac{q}{q^{\times }} \sum _{\begin{array}{c} m=1 \\ m\ne q^{\times }\,/2 \end{array}}^{q-2} q^{-1}\omega (2\psi )^{-4m}g(-2m)^2 g(4m) \\&= 2 + qH_q\left( \tfrac{1}{4}, \tfrac{3}{4}; 0, \tfrac{1}{2} \,|\, \psi ^{-4}\right) \end{aligned} \end{aligned}$$

as claimed. $$\square $$

##### Lemma 3.4.12

Suppose $$q \equiv 1 \pmod 4$$. Then

$$\begin{aligned} \sum _{s \in S_5} \omega (a)^{-s} c_s = (-1)^{q^{\times }\,/4} q H_q\left( \tfrac{1}{2}; 0 \,|\, \psi ^{-4}\right) + (-1)^{q^{\times }\,/4} - \frac{g\left( \tfrac{q^{\times }}{4}\right) ^2 + g\left( \tfrac{3q^{\times }}{4}\right) ^2}{g\left( \tfrac{q^{\times }}{2}\right) }. \end{aligned}$$

##### Proof

Plugging into the definition of the finite-field hypergeometric sum and then pulling out terms $m=jq^{\times }\,/4$ with $j=0,1,2,3$, we get

3.4.13 $$\begin{aligned} \begin{aligned} H_q\left( \tfrac{1}{2}; 0 \,|\, \psi ^{-4}\right)&= \frac{1}{q^{\times }} \sum _{m=0}^{q-2} \omega \left( -\psi ^{-4}\right) ^m \frac{g\left( m + \tfrac{q^{\times }}{2}\right) g\left( -m\right) }{g\left( \tfrac{q^{\times }}{2}\right) } \\&= -\frac{2}{q^{\times }} + \frac{\left( -1\right) ^{q^{\times }\,/4}}{q^{\times }g\left( \tfrac{q^{\times }}{2}\right) }\left( g\left( \tfrac{q^{\times }}{4}\right) ^2+g\left( \tfrac{3q^{\times }}{4}\right) ^2\right) \\&\quad + \frac{1}{q^{\times }} \sum _{\begin{array}{c} m=0 \\ q^{\times }\not \mid 4m \end{array}}^{q-2} \omega \left( -\psi ^{-4}\right) ^{m} \frac{g\left( m + \tfrac{q^{\times }}{2}\right) g\left( -m\right) }{g\left( \tfrac{q^{\times }}{2}\right) }. \end{aligned} \end{aligned}$$

Hasse–Davenport (Lemma 3.1.3(c)) implies

3.4.14 $$\begin{aligned} g(4m) = -\omega (4)^{4m} \frac{g(m) g\left( m + \tfrac{q^{\times }}{4}\right) g\left( m + \tfrac{q^{\times }}{2}\right) g\left( m + \tfrac{3q^{\times }}{4}\right) }{g(0) g\left( \tfrac{q^{\times }}{4}\right) g\left( \tfrac{q^{\times }}{2}\right) g\left( \tfrac{3q^{\times }}{4}\right) }. \end{aligned}$$

For $m \ne j\tfrac{q^{\times }}{4}$, multiplying (3.4.14) by $g(-m-\tfrac{3q^{\times }}{4})g(-4m)$ and simplifying, we get

3.4.15 $$\begin{aligned} \begin{aligned}&\left( -1\right) ^{4m} q g\left( -m-\tfrac{3q^{\times }}{4}\right) = \omega \left( 4\right) ^{4m}\left( -1\right) ^{m} \frac{g\left( m\right) g\left( m + \tfrac{q^{\times }}{4}\right) g\left( m + \tfrac{q^{\times }}{2}\right) g\left( -4m\right) }{ g\left( \tfrac{q^{\times }}{2}\right) }, \\&g\left( m\right) g\left( m + \tfrac{q^{\times }}{4}\right) g\left( m + \tfrac{q^{\times }}{2}\right) g\left( -4m\right) = \left( -1\right) ^{-m} \omega \left( 4\right) ^{-4m} q g\left( \tfrac{q^{\times }}{2}\right) g\left( -m-\tfrac{3q^{\times }}{4}\right) . \end{aligned} \end{aligned}$$

Now, we look at the point count. First, we take the definition

3.4.16 $$\begin{aligned} \begin{aligned} \sum _{s \in S_5} \omega (a)^{-s} c_s&= \frac{1}{qq^{\times }} \sum _{k=0}^{q-2} \omega (-4\psi )^{4k} g(k) g\left( k+\tfrac{q^{\times }}{4}\right) ^2 g\left( k + \tfrac{q^{\times }}{2}\right) g(-4k). \end{aligned} \end{aligned}$$

We then tease out the four terms with $k=jq^{\times }\,/4$. The cases $k=0,\frac{q^{\times }}{2}$ give

3.4.17 $$\begin{aligned} \frac{1}{qq^{\times }} g\left( \tfrac{q^{\times }}{4}\right) ^2 g\left( \tfrac{q^{\times }}{2}\right) + \frac{1}{qq^{\times }} g\left( \tfrac{q^{\times }}{2}\right) g\left( \tfrac{3q^{\times }}{4}\right) ^2 = \frac{g\left( \tfrac{q^{\times }}{4}\right) ^2+g\left( \tfrac{3q^{\times }}{4}\right) ^2}{q^{\times }g\left( \tfrac{q^{\times }}{2}\right) } \end{aligned}$$

because $g(\tfrac{q^{\times }}{2})^2=q$ as $q \equiv 1 \pmod {4}$. The terms with $k=\tfrac{q^{\times }}{4},\tfrac{3q^{\times }}{4}$ are

3.4.18 $$\begin{aligned}&-\frac{1}{qq^{\times }} g\left( \tfrac{q^{\times }}{4}\right) g\left( \tfrac{q^{\times }}{2}\right) ^2 g\left( \tfrac{3q^{\times }}{4}\right) -\frac{1}{qq^{\times }} g\left( \tfrac{3q^{\times }}{4}\right) g\left( \tfrac{q^{\times }}{4}\right) \nonumber \\&\quad = -\left( -1\right) ^{q^{\times }\,/4}\frac{q+1}{q^{\times }} = \left( -1\right) ^{q^{\times }\,/4}\left( 1-\frac{2q}{q^{\times }}\right) . \end{aligned}$$

using Lemma 3.1.3(b) with $m=\tfrac{q^{\times }}{4}$ to get $g(\tfrac{q^{\times }}{4})g(\tfrac{3q^{\times }}{4})=(-1)^{q^{\times }\,/4} q$.

For the remaining terms in the sum, we plug in (3.4.15) to get

3.4.19 $$\begin{aligned} \begin{aligned}&\frac{1}{qq^{\times }} \sum _{\begin{array}{c} k=0 \\ q^{\times }\not \mid 4k \end{array} }^{q-2} \omega \left( -4\psi \right) ^{4k} g\left( k\right) g\left( k+\tfrac{q^{\times }}{4}\right) ^2 g\left( k + \tfrac{q^{\times }}{2}\right) g\left( -4k\right) \\&\quad = \frac{1}{qq^{\times }} \sum _{\begin{array}{c} k=0 \\ q^{\times }\not \mid 4k \end{array} }^{q-2} \omega \left( -4\psi \right) ^{4k} g\left( k+\tfrac{q^{\times }}{4}\right) \left( -1\right) ^{-k} \omega \left( 4\right) ^{-4k} q g\left( \tfrac{q^{\times }}{2}\right) g\left( -k-\tfrac{3q^{\times }}{4}\right) \\&\quad =\frac{q}{q^{\times }} \sum _{\begin{array}{c} k=0 \\ q^{\times }\not \mid 4k \end{array} }^{q-2} \omega \left( -\psi ^4\right) ^{k} \frac{ g\left( k+\tfrac{q^{\times }}{4}\right) g\left( -k-\tfrac{3q^{\times }}{4}\right) }{g\left( \tfrac{q^{\times }}{2}\right) }. \end{aligned} \end{aligned}$$

Next, we reindex this summation with the substitution $m = -k - \tfrac{q^{\times }}{4}$ to obtain

3.4.20 $$\begin{aligned}&\frac{q}{q^{\times }} \sum _{\begin{array}{c} m=0 \\ q^{\times }\not \mid 4m \end{array} }^{q-2} \omega \left( -\psi ^4\right) ^{-m-q^{\times }\,/4} \frac{g\left( -m\right) g\left( m+ \tfrac{q^{\times }}{2}\right) }{g\left( \tfrac{q^{\times }}{2}\right) } \nonumber \\&\quad = \left( -1\right) ^{q^{\times }\,/4}\frac{q}{q^{\times }} \sum _{\begin{array}{c} m=0 \\ q^{\times }\not \mid 4m \end{array} }^{q-2} \omega \left( -\psi ^{-4}\right) ^{m} \frac{g\left( m+\tfrac{q^{\times }}{2}\right) g\left( -m\right) }{g\left( \tfrac{q^{\times }}{2}\right) }. \end{aligned}$$

Taking (3.4.16), expanding and substituting (3.4.17), (3.4.18), and (3.4.20) then give

$$\begin{aligned}&\left( -1\right) ^{q^{\times }\,/4}\sum _{s \in S_5} \omega \left( a\right) ^{-s} c_s \\&\quad = \left( -1\right) ^{q^{\times }\,/4}\frac{g\left( \tfrac{q^{\times }}{4}\right) ^2+g\left( \tfrac{3q^{\times }}{4}\right) ^2}{q^{\times }g\left( \tfrac{q^{\times }}{2}\right) } +1-\frac{2q}{q^{\times }} \\&\qquad + \frac{q}{q^{\times }} \sum _{\begin{array}{c} m=0 \\ q^{\times }\not \mid 4m \end{array} }^{q-2} \omega \left( -\psi ^{-4}\right) ^{m} \frac{g\left( m+\tfrac{q^{\times }}{2}\right) g\left( -m\right) }{g\left( \tfrac{q^{\times }}{2}\right) }. \end{aligned}$$

We are quite close to (3.4.13), but the first term is off by a factor *q*. Adding and subtracting give

$$\begin{aligned} \left( -1\right) ^{q^{\times }\,/4}\sum _{s \in S_5} \omega \left( a\right) ^{-s} c_s = 1+qH_q\left( \tfrac{1}{2};0 \,|\,\psi ^{-4}\right) - \left( -1\right) ^{q^{\times }\,/4}\frac{g\left( \tfrac{q^{\times }}{4}\right) ^2+g\left( \tfrac{3q^{\times }}{4}\right) ^2}{g\left( \tfrac{q^{\times }}{2}\right) } \end{aligned}$$

as claimed. $$\square $$

##### Lemma 3.4.21

If $$q \equiv 1 \pmod 4$$, then

$$\begin{aligned} \sum _{s \in S_5} \omega (a)^{-s} c_s = \sum _{s \in S_6} \omega (a)^{-s} c_s = \sum _{s \in S_7} \omega (a)^{-s} c_s. \end{aligned}$$

##### Proof

We start with (3.4.16) and reindex with $m = k+\tfrac{q^{\times }}{2}$:

$$\begin{aligned} \sum _{s \in S_5} \omega \left( a\right) ^{-s} c_s&= \frac{1}{qq^{\times }} \sum _{k=0}^{q-2} \omega \left( -4\psi \right) ^{4k} g\left( k\right) g\left( k+\tfrac{q^{\times }}{4}\right) ^2 g\left( k + \tfrac{q^{\times }}{2}\right) g\left( -4k\right) \\&= \frac{1}{qq^{\times }} \sum _{m=0}^{q-2} \omega \left( -4\psi \right) ^{4m} g\left( m + \tfrac{q^{\times }}{2}\right) g\left( m+\tfrac{3q^{\times }}{4}\right) ^2 g\left( m\right) g\left( -4m\right) \\&= \sum _{s \in S_6} \omega \left( a\right) ^{-s} c_s. \end{aligned}$$

The equality for $S_7$ holds reindexing with $m=k+\tfrac{q^{\times }}{4}$. $\square$

We now put these pieces together to give the point count for the toric hypersurface.

##### Proposition 3.4.22

Let $$\psi \in {\mathbb {F}}_q^\times $$.

- If $$q \equiv 3 \pmod 4$$, then
  3.4.23 $$\begin{aligned} \#U_{{\mathsf {F}}_4, \psi }\left( {\mathbb {F}}_q\right) = q^2 -3q -3 + H_q\left( \tfrac{1}{4}, \tfrac{1}{2}, \tfrac{3}{4}; 0,0,0 \,|\, \psi ^{-4}\right) - 3 qH_q\left( \tfrac{1}{4}, \tfrac{3}{4}; 0, \tfrac{1}{2} \,|\, \psi ^{-4}\right) ). \end{aligned}$$
- If $$q \equiv 1 \pmod 4$$, then
  3.4.24 $$\begin{aligned} \begin{aligned} \#U_{{\mathsf {F}}_4, \psi }\left( {\mathbb {F}}_q\right)&= q^2-3q+9 + H_q\left( \tfrac{1}{4}, \tfrac{1}{2}, \tfrac{3}{4}; 0,0,0 \,|\, \psi ^{-4}\right) + 3qH_q\left( \tfrac{1}{4}, \tfrac{3}{4}; 0, \tfrac{1}{2} \,|\, \psi ^{-4}\right) \\&\quad + 12\left( \left( -1\right) ^{q^{\times }\,/4}q H_q\left( \tfrac{1}{2}; 0 \,|\, \psi ^{-4}\right) + \left( -1\right) ^{q^{\times }\,/4} - \frac{g\left( \tfrac{q^{\times }}{4}\right) ^2 + g\left( \tfrac{3q^{\times }}{4}\right) ^2}{g\left( \tfrac{q^{\times }}{2}\right) }\right) . \end{aligned} \end{aligned}$$

##### Proof

For $q\equiv 3\pmod 4$, we have from Lemmas 3.4.4 and 3.4.7:

3.4.25 $$\begin{aligned} \begin{aligned} \#U_{{\mathsf {F}}_4, \psi }\left( {\mathbb {F}}_q\right)&= \sum _{i=1}^4 \sum _{s \in S_i} \omega \left( a\right) ^{-s} c_s \\&= q^2-3q+3 + H_q\left( \tfrac{1}{4}, \tfrac{1}{2}, \tfrac{3}{4}; 0,0,0 \,|\, \psi ^{-4}\right) + 3\left( -2 - qH_q\left( \tfrac{1}{4}, \tfrac{3}{4}; 0, \tfrac{1}{2} \,|\, \psi ^{-4}\right) \right) \\&=q^2 -3q -3 + H_q\left( \tfrac{1}{4}, \tfrac{1}{2}, \tfrac{3}{4}; 0,0,0 \,|\, \psi ^{-4}\right) - 3 qH_q\left( \tfrac{1}{4}, \tfrac{3}{4}; 0, \tfrac{1}{2} \,|\, \psi ^{-4}\right) ) \end{aligned} \end{aligned}$$

For $q\equiv 1 \pmod 4$, we have from Lemmas 3.4.4, 3.4.7,  3.4.12, and 3.4.21, we have that:

3.4.26 $$\begin{aligned} \begin{aligned} \#U_{{\mathsf {F}}_4, \psi }\left( {\mathbb {F}}_q\right)&= \sum _{i=1}^4 \sum _{s \in S_i} \omega \left( a\right) ^{-s} c_s + 12\sum _{s \in S_5} \omega \left( a\right) ^{-s} c_s \\&= q^2-3q+3 + H_q\left( \tfrac{1}{4}, \tfrac{1}{2}, \tfrac{3}{4}; 0,0,0 \,|\, \psi ^{-4}\right) \\&\quad + 3\left( 2 + qH_q\left( \tfrac{1}{4}, \tfrac{3}{4}; 0, \tfrac{1}{2} \,|\, \psi ^{-4}\right) \right) \\&\qquad + 12\left( \left( -1\right) ^{q^{\times }\,/4} q H_q\left( \tfrac{1}{2}; 0 \,|\, \psi ^{-4}\right) + \left( -1\right) ^{q^{\times }\,/4} - \frac{g\left( \tfrac{q^{\times }}{4}\right) ^2 + g\left( \tfrac{3q^{\times }}{4}\right) ^2}{g\left( \tfrac{q^{\times }}{2}\right) }\right) , \end{aligned} \end{aligned}$$

which simplifies to the result. $\square$

#### Step 3: Count points when at least one coordinate is zero

##### Lemma 3.4.27

If $$q \equiv 3 \pmod 4$$, then

$$\begin{aligned} \#X_{{\mathsf {F}}_4,\psi }({\mathbb {F}}_q) - \#U_{{\mathsf {F}}_4,\psi }({\mathbb {F}}_q) = 4q+4. \end{aligned}$$

##### Proof

First, we compute the number of points when $x_3=0$ and $x_0x_1x_2 \ne 0$, i.e., count points on the Fermat quartic curve $V:x_0^4+x_1^4+x_2^4=0$ with coordinates in the torus. All points in $V({\mathbb {F}}_q)$ lie on the torus: If, e.g., $x_0=0$ and $x_1 \ne 0$, then $-1=(x_2/x_1)^4$, but $-1 \not \in {\mathbb {F}}_q^{\times 2}$ since $q \equiv 3 \pmod {4}$. We claim that $\#V({\mathbb {F}}_q)=q+1$; this can be proven in many ways. First, we sketch an elementary argument, working affinely on $x_0^4+x_1^4=-1$. The map $(x_0,x_1) \mapsto (x_0^2,x_1^2)$ gives a map to the affine curve defined by $C:w_0^2+w_1^2=-1$. The number of points on this curve over ${\mathbb {F}}_q$ is $q+1$ (the projective closure is a smooth conic with no points at infinity), and again all such solutions have $w_0,w_1 \in {\mathbb {F}}_{q}^\times$. Since $q \equiv 3 \pmod {4}$, the squaring map ${\mathbb {F}}_q^{\times 2} \rightarrow {\mathbb {F}}_q^{\times 4}$ is bijective. Therefore, for the four points $(\pm w_0,\pm w_1)$ with $w_0^2+w_1^2=-1$, there are exactly four points $(\pm x_0, \pm x_1)$ with $x_i^4=w_i^2$ for $i=0,1$. Thus, $\#V({\mathbb {F}}_q)=\#C({\mathbb {F}}_q)=q+1$. (Alternatively, the map $(x_0,x_1) \mapsto (x_0^2,x_1)$ is bijective, with image a supersingular genus 1 curve over ${\mathbb {F}}_q$).

Second, and for consistency, we again apply the formula of Koblitz! For the characters, we solve

3.4.28 $$\begin{aligned} \begin{pmatrix} 4&{}\quad 0&{}\quad 0\\ 0&{}\quad 4&{}\quad 0\\ 0&{}\quad 0&{}\quad 4\\ 1&{}\quad 1&{}\quad 1 \end{pmatrix} \begin{pmatrix} s_1 \\ s_2 \\ s_3 \end{pmatrix} \equiv 0 \pmod {q^{\times }}. \end{aligned}$$

There are exactly four solutions when $q \equiv 3 \pmod 4$:

$$\begin{aligned} S= \left\{ (0,0,0), \tfrac{q^{\times }}{2} (1,1,0), \tfrac{q^{\times }}{2} (1,0,1), \tfrac{q^{\times }}{2} (0,1,1)\right\} . \end{aligned}$$

Then by Theorem 3.3.3,

3.4.29 $$\begin{aligned} \begin{aligned} \#V({\mathbb {F}}_q)&= c_{(0,0,0)} + c_{(q^{\times }\,/2)(1,1,0)} + c_{(q^{\times }\,/2)(1,0,1)}+ c_{(q^{\times }\,/2)(0,1,1)} \\&= \frac{(q-1)^{2} - (-1)^2}{q} + 3(-1)^{2} \frac{1}{q} g\left( \tfrac{q^{\times }}{2}\right) ^2 g\left( \tfrac{q^{\times }}{2}\right) g(0) \\&= \frac{q^2 - 2q}{q} + 3 = q+1. \end{aligned} \end{aligned}$$

By symmetry, repeating in each of the four coordinate hyperplanes, we obtain $\#X_{{\mathsf {F}}_4,\psi }({\mathbb {F}}_q) - \#U_{{\mathsf {F}}_4,\psi }({\mathbb {F}}_q) = 4(q+1) = 4q + 4$. $\square$

##### Lemma 3.4.30

If $$q\equiv 1\pmod 4$$, then

$$\begin{aligned} \#X_{{\mathsf {F}}_4, \psi }({\mathbb {F}}_q) - \#U_{{\mathsf {F}}_4, \psi }({\mathbb {F}}_q) = 4q - 8 -12(-1)^{q^{\times }\,/4} + 12\frac{g\left( \tfrac{q^{\times }}{4}\right) ^2+g\left( \tfrac{3q^{\times }}{4}\right) ^2}{g\left( \tfrac{q^{\times }}{2}\right) }. \end{aligned}$$

##### Proof

We repeat the argument in the preceding lemma. We cluster solutions to (3.4.28) and count the number of solutions in the following way:

3.4.31 $$\begin{aligned} \begin{aligned} \#V\left( {\mathbb {F}}_q\right)&= c_{\left( 0,0,0\right) } + 6c_{\left( q^{\times }\,/4\right) \left( 1,3,0\right) } + 3c_{\left( q^{\times }\,/4\right) \left( 2,2,0\right) } + 3c_{\left( q^{\times }\,/4\right) \left( 1,1,2\right) } + 3c_{\left( q^{\times }\,/4\right) \left( 3,3,2\right) } \\&= q-2 - 6 \left( -1\right) ^{q^{\times }\,/4} - 3 + \frac{3}{q} g\left( \tfrac{q^{\times }}{4}\right) ^2 g\left( \tfrac{q^{\times }}{2}\right) + \frac{3}{q} g\left( \tfrac{3q^{\times }}{4}\right) ^2g\left( \tfrac{q^{\times }}{2}\right) \\&= q-5 - 6 \left( -1\right) ^{q^{\times }\,/4} + 3\frac{g\left( \tfrac{q^{\times }}{4}\right) ^2+g\left( \tfrac{3q^{\times }}{4}\right) ^2}{g\left( \tfrac{q^{\times }}{2}\right) }. \end{aligned} \end{aligned}$$

There are 2 solutions to $x_0^4+x_1^4=0$ with $x_0x_1 \ne 0$ if $q \equiv 1 \pmod {8}$ and zero otherwise, so $2+2(-1)^{q^{\times }\,/4}$ solutions in either case. Adding up, we get

$$\begin{aligned} \#X_{{\mathsf {F}}_4, \psi }\left( {\mathbb {F}}_q\right) - \#U_{{\mathsf {F}}_4, \psi }\left( {\mathbb {F}}_q\right)&= 4 \#V\left( {\mathbb {F}}_q\right) + 6\left( 2+2\left( -1\right) ^{q^{\times }\,/4}\right) \\&= 4q - 8 -12\left( -1\right) ^{q^{\times }\,/4} + 12 \frac{1}{q} g\left( \tfrac{q^{\times }}{4}\right) ^2 g\left( \tfrac{q^{\times }}{2}\right) \\&\quad + 12\frac{g\left( \tfrac{q^{\times }}{4}\right) ^2+g\left( \tfrac{3q^{\times }}{4}\right) ^2}{g\left( \tfrac{q^{\times }}{2}\right) }. \end{aligned}$$

$$\square $$

#### Step 4: Conclude

We now conclude the proof.

##### Proof of Proposition 3.4.1

We combine Proposition 3.4.22 with Lemmas 3.4.27 and 3.4.30. If $q\equiv 3 \pmod 4$, then

$$\begin{aligned} \#X_{{\mathsf {F}}_4, \psi }\left( {\mathbb {F}}_q\right)&= \#U_{{\mathsf {F}}_4, \psi }\left( {\mathbb {F}}_q\right) + \left( \#X_{{\mathsf {F}}_4, \psi }\left( {\mathbb {F}}_q\right) - \#U_{{\mathsf {F}}_4, \psi }\left( {\mathbb {F}}_q\right) \right) \\&= \left( q^2 -3q -3 + H_q\left( \tfrac{1}{4}, \tfrac{1}{2}, \tfrac{3}{4}; 0,0,0 \,|\, \psi ^{-4}\right) \right. \\&\qquad \left. -\, 3 qH_q\left( \tfrac{1}{4}, \tfrac{3}{4}; 0, \tfrac{1}{2} \,|\, \psi ^{-4}\right) \right) + \left( 4q+4\right) \\&= q^2 + q + 1 + H_q\left( \tfrac{1}{4}, \tfrac{1}{2}, \tfrac{3}{4}; 0,0,0 \,|\, \psi ^{-4}\right) - 3 qH_q\left( \tfrac{1}{4}, \tfrac{3}{4}; 0, \tfrac{1}{2} \,|\, \psi ^{-4}\right) . \end{aligned}$$

If $q\equiv 1\pmod 4$, then the ugly terms cancel, and we have simply

$$\begin{aligned} \#X_{{\mathsf {F}}_4, \psi }\left( {\mathbb {F}}_q\right)&= \#U_{{\mathsf {F}}_4, \psi }\left( {\mathbb {F}}_q\right) + \left( \#X_{{\mathsf {F}}_4, \psi }\left( {\mathbb {F}}_q\right) - \#U_{{\mathsf {F}}_4, \psi }\left( {\mathbb {F}}_q\right) \right) \\&= \left( q^2-3q+9 + H_q\left( \tfrac{1}{4}, \tfrac{1}{2}, \tfrac{3}{4}; 0,0,0 \,|\, \psi ^{-4}\right) + 3qH_q\left( \tfrac{1}{4}, \tfrac{3}{4}; 0, \tfrac{1}{2} \,|\, \psi ^{-4}\right) \right. \\&\qquad \left. + 12\left( -1\right) ^{q^{\times }\,/4}q H_q\left( \tfrac{1}{2}; 0 \,|\, \psi ^{-4}\right) \right) + \left( 4q-8\right) \\&= q^2 + q + 1 + H_q\left( \tfrac{1}{4}, \tfrac{1}{2}, \tfrac{3}{4}; 0,0,0 \,|\, \psi ^{-4}\right) + 3qH_q\left( \tfrac{1}{4}, \tfrac{3}{4}; 0, \tfrac{1}{2} \,|\, \psi ^{-4}\right) \\&\qquad + 12\left( -1\right) ^{q^{\times }\,/4} q H_q\left( \tfrac{1}{2}; 0 \,|\, \psi ^{-4}\right) . \end{aligned}$$

$$\square $$

### The Klein–Mukai pencil ${\mathsf {F}}_1{\mathsf {L}}_3$

In this section, we repeat the steps of the previous section but for the Klein–Mukai pencil ${\mathsf {F}}_1{\mathsf {L}}_3$. We suppose throughout this section that *q* is coprime to 14. Our main result is as follows.

#### Proposition 3.5.1

For *q* coprime to 14 and $\psi \in {\mathbb {F}}_q^\times$, the following statements hold.

- If $$q\not \equiv 1\pmod 7$$, then
  $$\begin{aligned} \#X_{{\mathsf {F}}_1{\mathsf {L}}_3, \psi }\left( {\mathbb {F}}_q\right) =q^2+q+1+H_q\left( \tfrac{1}{4},\tfrac{1}{2},\tfrac{3}{4};0,0,0\,|\,\psi ^{-4}\right) . \end{aligned}$$
- If $$q\equiv 1\pmod 7$$, then
  $$\begin{aligned} \#X_{{\mathsf {F}}_1{\mathsf {L}}_3, \psi }\left( {\mathbb {F}}_q\right)&=q^2+q+1+H_q\left( \tfrac{1}{4},\tfrac{1}{2},\tfrac{3}{4};0,0,0\,|\,\psi ^{-4}\right) \\&\quad +3qH_q\left( \tfrac{1}{14},\tfrac{9}{14},\tfrac{11}{14};0,\tfrac{1}{4},\tfrac{3}{4}\,|\,\psi ^4\right) \\&\quad +3qH_q\left( \tfrac{3}{14},\tfrac{5}{14},\tfrac{13}{14};0,\tfrac{1}{4},\tfrac{3}{4}\,|\,\psi ^4\right) . \end{aligned}$$

#### Remark 3.5.2

The new parameters $\tfrac{1}{14},\tfrac{9}{14},\tfrac{11}{14};0,\tfrac{1}{4},\tfrac{3}{4}$ and $\tfrac{3}{14},\tfrac{5}{14},\tfrac{13}{14};0,\tfrac{1}{4},\tfrac{3}{4}$ match the Picard–Fuchs equations in Proposition 2.6.1 as elements of ${\mathbb {Q}}/{\mathbb {Z}}$, with the same multiplicity.

#### Step 1: Computing and clustering the characters

As before, we first have to compute the solutions to the system of congruences:

$$\begin{aligned} \begin{pmatrix} 3&{}\quad 0&{}\quad 1&{}\quad 0&{}\quad 1\\ 1&{}\quad 3&{}\quad 0&{}\quad 0&{}\quad 1\\ 0&{}\quad 1&{}\quad 3&{}\quad 0&{}\quad 1\\ 0&{}\quad 0&{}\quad 0&{}\quad 4&{}\quad 1\\ 1&{}\quad 1&{}\quad 1&{}\quad 1&{}\quad 1\\ \end{pmatrix}\begin{pmatrix} w_1\\ w_2\\ w_3\\ w_4\\ w_5 \end{pmatrix} \equiv \begin{pmatrix} 0\\ 0\\ 0\\ 0\\ 0\end{pmatrix} \pmod {q^{\times }}. \end{aligned}$$

By linear algebra over ${\mathbb {Z}}$, we compute that if $q\not \equiv 1\pmod 7$, then the set of solutions is

$$\begin{aligned} S=\{(1,1,1,1,-4)w: w\in {\mathbb {Z}}/q^{\times }{\mathbb {Z}}\}. \end{aligned}$$

On the other hand if $q \equiv 1 \pmod {7}$, then the set splits into three classes:

(i) the set $$S_1 = \{k(1,1,1,1,-4): k\in {\mathbb {Z}}/q^{\times }{\mathbb {Z}}\}$$,
(ii) three sets of the form $S_8 = \left\{ k(1,1,1,1,-4) + \tfrac{q^{\times }}{7}(1,4,2,0,0): k\in {\mathbb {Z}}/q^{\times }{\mathbb {Z}}\right\}$, and
(iii) three sets of the form $S_9 = \left\{ k(1,1,1,1,-4) + \tfrac{q^{\times }}{7}(3,5,6,0,0): k\in {\mathbb {Z}}/q^{\times }{\mathbb {Z}}\right\}$.

The multiplicity of the latter two sets corresponds to cyclic permutations yielding the same product of Gauss sums.

#### Step 2: Counting points on the open subset with nonzero coordinates

As in the previous section, the hard work is in counting points in the toric hypersurface. We now proceed with each cluster.

##### Lemma 3.5.3

If $$q \equiv 1 \pmod 7$$, then

3.5.4 $$\begin{aligned} \sum _{s \in S_8} \omega \left( a\right) ^{-s} c_s = qH_q\left( \tfrac{1}{14}, \tfrac{9}{14}, \tfrac{11}{14}; 0, \tfrac{1}{4}, \tfrac{3}{4} \,|\, \psi ^4\right) - \frac{1}{q} g\left( \tfrac{q^{\times }}{7}\right) g\left( \tfrac{4q^{\times }}{7}\right) g\left( \tfrac{2q^{\times }}{7}\right) . \end{aligned}$$

##### Proof

Recall our hybrid hypergeometric sum (3.2.14) from Example 3.2.13, plugging in $t=\psi ^4$:

3.5.5 $$\begin{aligned} \begin{aligned}&H_q\left( \tfrac{1}{14}, \tfrac{9}{14}, \tfrac{11}{14}; 0,\tfrac{1}{4},\tfrac{3}{4} \,|\, \psi ^4\right) \\&\quad = \frac{1}{q^{\times }} -\frac{1}{qq^{\times }} g\left( \tfrac{q^{\times }}{7}\right) g\left( \tfrac{2q^{\times }}{7}\right) g\left( \tfrac{4q^{\times }}{7}\right) \\&\qquad +\frac{1}{qq^{\times }} \sum _{\begin{array}{c} m=1 \\ m \ne q^{\times }\,/2 \end{array}}^{q-2} \frac{g\left( m+ \frac{1}{14}q^{\times }\right) g\left( m+ \frac{9}{14}q^{\times }\right) g\left( m+ \frac{11}{14}q^{\times }\right) }{g\left( \frac{1}{14} q^{\times }\right) g\left( \frac{9}{14} q^{\times }\right) g\left( \frac{11}{14} q^{\times }\right) } \\&\qquad \cdot g\left( 2m\right) g\left( -m\right) g\left( -4m\right) \omega \left( -4^3\psi ^4\right) ^m. \end{aligned} \end{aligned}$$

Our point count formula expands to

$$\begin{aligned} \sum _{s \in S_8} \omega \left( a\right) ^{-s} c_s&= \frac{1}{qq^{\times }} g\left( \tfrac{q^{\times }}{7}\right) g\left( \tfrac{4q^{\times }}{7}\right) g\left( \tfrac{2q^{\times }}{7}\right) - \frac{1}{qq^{\times }} g\left( \tfrac{9q^{\times }}{14}\right) g\left( \tfrac{q^{\times }}{14}\right) g\left( \tfrac{11q^{\times }}{14}\right) g\left( \tfrac{q^{\times }}{2}\right) \\&\quad +\frac{1}{qq^{\times }} \sum _{\begin{array}{c} k=0 \\ q^{\times }\not \mid 2k \end{array}}^{q-2} \omega \left( -4\psi \right) ^{4k} g\left( k+\tfrac{q^{\times }}{7}\right) \\&\quad \cdot \, g\left( k+\tfrac{4q^{\times }}{7}\right) g\left( k + \tfrac{2q^{\times }}{7}\right) g\left( k\right) g\left( -4k\right) . \end{aligned}$$

We work on the sum. Changing indices to $m=k+\tfrac{q^{\times }}{2}$, using the identity

$$\begin{aligned} g\left( m+\tfrac{q^{\times }}{2}\right) = \omega \left( 4\right) ^{-m} \left( -1\right) ^mq^{-1} g\left( \tfrac{q^{\times }}{2}\right) g\left( -m\right) g\left( 2m\right) \end{aligned}$$

found by using Hasse–Davenport for $N=2$, and applying Lemma 3.2.11, gives us the summand

$$\begin{aligned}&\omega \left( -4\psi \right) ^{4m} g\left( m+\tfrac{9q^{\times }}{14}\right) g\left( m+\tfrac{q^{\times }}{14}\right) g\left( m + \tfrac{11q^{\times }}{14}\right) g\left( m + \tfrac{q^{\times }}{2}\right) g\left( -4m\right) \\&\quad =\omega \left( -4\psi \right) ^{4m} g\left( m+\tfrac{q^{\times }}{14}\right) g\left( m+\tfrac{9q^{\times }}{14}\right) g\left( m + \tfrac{11q^{\times }}{14}\right) \\&\quad \cdot \omega \left( 4\right) ^{-m} \left( -1\right) ^mq^{-1} g\left( \tfrac{q^{\times }}{2}\right) g\left( -m\right) g\left( 2m\right) g\left( -4m\right) \\&\quad =\omega \left( -4^3\psi ^4\right) ^{m}\frac{g\left( m+\tfrac{q^{\times }}{14}\right) g\left( m+\tfrac{9q^{\times }}{14}\right) g\left( m + \tfrac{11q^{\times }}{14}\right) }{g\left( \tfrac{q^{\times }}{14}\right) g\left( \tfrac{9q^{\times }}{14}\right) g\left( \tfrac{11q^{\times }}{14}\right) }\\&\quad \cdot q^{-1} g\left( \tfrac{q^{\times }}{2}\right) ^4 g\left( 2m\right) g\left( -m\right) g\left( -4m\right) \\&\quad =q\omega \left( -4^3\psi ^4\right) ^{m}\frac{g\left( m+\tfrac{q^{\times }}{14}\right) g\left( m+\tfrac{9q^{\times }}{14}\right) g\left( m + \tfrac{11q^{\times }}{14}\right) }{q g\left( \tfrac{q^{\times }}{14}\right) g\left( \tfrac{9q^{\times }}{14}\right) g\left( \tfrac{11q^{\times }}{14}\right) }g\left( 2m\right) g\left( -m\right) g\left( -4m\right) . \end{aligned}$$

Plugging back in, we can relate this to hypergeometric function (3.5.5):

$$\begin{aligned}&\sum _{s \in S_8} \omega \left( a\right) ^{-s} c_s = \frac{1}{qq^{\times }} g\left( \tfrac{q^{\times }}{7}\right) g\left( \tfrac{2q^{\times }}{7}\right) g\left( \tfrac{4q^{\times }}{7}\right) - \frac{1}{qq^{\times }} g\left( \tfrac{q^{\times }}{14}\right) g\left( \tfrac{9q^{\times }}{14}\right) g\left( \tfrac{11q^{\times }}{14}\right) g\left( \tfrac{q^{\times }}{2}\right) \\&\qquad + \frac{1}{qq^{\times }} \sum _{\begin{array}{c} m=0 \\ q^{\times }\not \mid 2m \end{array}}^{q-2} q\omega \left( -4^3\psi ^4\right) ^{m}\frac{g\left( m+\tfrac{q^{\times }}{14}\right) g\left( m+\tfrac{9q^{\times }}{14}\right) g\left( m + \tfrac{11q^{\times }}{14}\right) }{g\left( \tfrac{q^{\times }}{14}\right) g\left( \tfrac{9q^{\times }}{14}\right) g\left( \tfrac{11q^{\times }}{14}\right) }\\&\quad \cdot g\left( 2m\right) g\left( -m\right) g\left( -4m\right) \\&\quad = \frac{1}{qq^{\times }} g\left( \tfrac{q^{\times }}{7}\right) g\left( \tfrac{2q^{\times }}{7}\right) g\left( \tfrac{4q^{\times }}{7}\right) - \frac{1}{qq^{\times }} g\left( \tfrac{q^{\times }}{14}\right) g\left( \tfrac{9q^{\times }}{14}\right) g\left( \tfrac{11q^{\times }}{14}\right) g\left( \tfrac{q^{\times }}{2}\right) \\&\qquad + qH_q\left( \tfrac{1}{14}, \tfrac{9}{14}, \tfrac{11}{14}; 0, \tfrac{1}{4}, \tfrac{3}{4} \,|\, \psi ^4\right) + \frac{q}{q^{\times }} - \frac{1}{q^{\times }}g\left( \tfrac{q^{\times }}{7}\right) g\left( \tfrac{2q^{\times }}{7}\right) g\left( \tfrac{4q^{\times }}{7}\right) \\&\quad = qH_q\left( \tfrac{1}{14}, \tfrac{9}{14}, \tfrac{11}{14}; 0, \tfrac{1}{4}, \tfrac{3}{4} \,|\, \psi ^4\right) - \frac{1}{q} g\left( \tfrac{q^{\times }}{7}\right) g\left( \tfrac{2q^{\times }}{7}\right) g\left( \tfrac{4q^{\times }}{7}\right) . \end{aligned}$$

$$\square $$

##### Lemma 3.5.6

If $$q \equiv 1 \pmod 7$$, then

3.5.7 $$\begin{aligned} \sum _{s \in S_9} \omega \left( a\right) ^{-s} c_s = qH_q\left( \tfrac{3}{14}, \tfrac{5}{14}, \tfrac{13}{14}; 0, \tfrac{1}{4}, \tfrac{3}{4} \,|\, \psi ^4\right) - \frac{1}{q} g\left( \tfrac{3q^{\times }}{7}\right) g\left( \tfrac{5q^{\times }}{7}\right) g\left( \tfrac{6q^{\times }}{7}\right) . \end{aligned}$$

##### Proof

Apply complex conjugation to Lemma 3.5.3; the effect is to negate indices, as in Proposition 3.2.8. $\square$

We now put the pieces together to prove the main result in this step.

##### Proposition 3.5.8

Suppose $$\psi \in {\mathbb {F}}_q^{\times }$$.

- If $$q \not \equiv 1 \pmod 7$$, then
  3.5.9 $$\begin{aligned} \#U_{{\mathsf {F}}_1{\mathsf {L}}_3, \psi }\left( {\mathbb {F}}_q\right) = q^2-3q+3 + H_q\left( \tfrac{1}{4}, \tfrac{1}{2}, \tfrac{3}{4}; 0,0,0 \,|\, \psi ^{-4}\right) . \end{aligned}$$
- If $$q\equiv 1 \pmod 7$$, then
  3.5.10 $$\begin{aligned} \#U_{{\mathsf {F}}_1{\mathsf {L}}_3, \psi }\left( {\mathbb {F}}_q\right)&= q^2-3q+3 + H_q\left( \tfrac{1}{4}, \tfrac{1}{2}, \tfrac{3}{4}; 0,0,0 \,|\, \psi ^{-4}\right) \nonumber \\&\quad + 3qH_q\left( \tfrac{1}{14}, \tfrac{9}{14}, \tfrac{11}{14}; 0, \tfrac{1}{4}, \tfrac{3}{4} \,|\, \psi ^4\right) + 3qH_q\left( \tfrac{3}{14}, \tfrac{5}{14}, \tfrac{13}{14}; 0, \tfrac{1}{4}, \tfrac{3}{4} \,|\, \psi ^4\right) \nonumber \\&\quad -\frac{3}{q}\left( g\left( \tfrac{q^{\times }}{7}\right) g\left( \tfrac{2q^{\times }}{7}\right) g\left( \tfrac{4q^{\times }}{7}\right) + g\left( \tfrac{3q^{\times }}{7}\right) g\left( \tfrac{5q^{\times }}{7}\right) g\left( \tfrac{6q^{\times }}{7}\right) \right) .\nonumber \\ \end{aligned}$$

##### Proof

When $q \not \equiv 1 \pmod 7$, there is only one cluster of characters, $S_1$. By Lemma 3.4.4, we know that

3.4.11 $$\begin{aligned} \#U_{{\mathsf {F}}_1{\mathsf {L}}_3, \psi }\left( {\mathbb {F}}_q\right) = \sum _{s \in S_1} \omega \left( a\right) ^{-s} c_s= q^2-3q+3 + H_q\left( \tfrac{1}{4}, \tfrac{1}{2}, \tfrac{3}{4}; 0,0,0 \,|\, \psi ^{-4}\right) . \end{aligned}$$

When $q\equiv 1 \pmod 7$, we have three clusters of characters, the latter two ($S_8$ and $S_9$) with multiplicity 3. By Lemmas 3.4.4, 3.5.3, and 3.5.6, these sum to the result. $\square$

#### Step 3: Count points when at least one coordinate is zero.

Recall that *q* is coprime to 14.

##### Lemma 3.5.12

Let $$\psi \in {\mathbb {F}}_q^\times $$.

- If $$q\not \equiv 1 \pmod 7$$, then
  $$\begin{aligned} \#X_{{\mathsf {F}}_1{\mathsf {L}}_3, \psi }({\mathbb {F}}_q) - \#U_{{\mathsf {F}}_1{\mathsf {L}}_3, \psi }({\mathbb {F}}_q) = 4q-2. \end{aligned}$$
- If $$q\equiv 1 \pmod 7$$, then
  $$\begin{aligned} \#X_{{\mathsf {F}}_1{\mathsf {L}}_3, \psi }\left( {\mathbb {F}}_q\right) - \#U_{{\mathsf {F}}_1{\mathsf {L}}_3, \psi }\left( {\mathbb {F}}_q\right)&= 4q - 2 + \frac{3}{q}(g\left( \tfrac{q^{\times }}{7}\right) g\left( \tfrac{2q^{\times }}{7}\right) g\left( \tfrac{4q^{\times }}{7}\right) \\&\quad + g\left( \tfrac{3q^{\times }}{7}g\left( \tfrac{5q^{\times }}{7}\right) g\left( \tfrac{6q^{\times }}{7}\right) \right) . \end{aligned}$$

##### Proof

We count solutions with at least one coordinate zero. If $x_3=0$ but $x_0x_1x_2 \ne 0$, we count points on $x_0^4+x_1^3x_2=0$: solving for $x_2$, we see there are $q-1$ solutions; repeating this for the cases $x_1=0$ or $x_2=0$, we get $3q-3$ points.

Now, suppose $x_0=0$ but $x_1x_2x_3 \ne 0$, we look at the equation $x_1^3x_2+x_2^3x_3+x_3^3x_1=0$ defining the Klein quartic. Applying Theorem 3.3.3 again, we find that

3.5.13 $$\begin{aligned} \begin{pmatrix} 3&{}\quad 1&{}\quad 0\\ 0&{}\quad 3&{}\quad 1\\ 1&{}\quad 0&{}\quad 3\\ 1&{}\quad 1&{}\quad 1 \end{pmatrix} \begin{pmatrix} s_1 \\ s_2 \\ s_3 \end{pmatrix} \equiv 0 \pmod {q^{\times }}. \end{aligned}$$

If $q\not \equiv 1\pmod 7$, then only (0, 0, 0) is a solution and $c_{(0,0,0)} = q-2$. If $q \equiv 1 \pmod 7$, then the solutions are $\left\{ k(\tfrac{q^{\times }}{7},\tfrac{4q^{\times }}{7},\tfrac{2q^{\times }}{7}) : k\in {\mathbb {Z}}/7{\mathbb {Z}}\right\}$ which gives the point count

$$\begin{aligned} q-2 + \frac{3}{q}g\left( \tfrac{q^{\times }}{7}\right) g\left( \tfrac{2q^{\times }}{7}\right) g\left( \tfrac{4q^{\times }}{7}\right) + \frac{3}{q}g\left( \tfrac{3q^{\times }}{7}\right) g\left( \tfrac{5q^{\times }}{7}\right) g\left( \tfrac{6q^{\times }}{7}\right) . \end{aligned}$$

If now at least two of the variables among $\{x_1,x_2,x_3\}$ are zero, then the equation is just $x_0^4 = 0$; hence, the last one is also zero and there is only one such point. If $x_0=x_1=0$, then the equation is $x_2^3 x_3=0$; hence, another of the first three variables is zero. Consequently, there are exactly 3 such points. Totaling up gives the result. $\square$

#### Step 4: Conclude

We now prove Proposition 3.5.1.

##### Proof of Proposition 3.5.1

By Proposition 3.5.8 and Lemma 3.5.12, if $q\not \equiv 1\pmod 7$, then

3.4.14 $$\begin{aligned} \begin{aligned} \#X_{{\mathsf {F}}_1{\mathsf {L}}_3, \psi }\left( {\mathbb {F}}_q\right)&= q^2-3q+3 + H_q\left( \tfrac{1}{4}, \tfrac{1}{2}, \tfrac{3}{4}; 0,0,0 \,|\, \psi ^{-4}\right) + \left( 4q-2\right) \\&= q^2 + q + 1+ H_q\left( \tfrac{1}{4}, \tfrac{1}{2}, \tfrac{3}{4}; 0,0,0 \,|\, \psi ^{-4}\right) . \end{aligned} \end{aligned}$$

If $q\equiv 1 \pmod 7$, then the ugly terms cancel and we get

3.4.15 $$\begin{aligned} \begin{aligned} \#X_{{\mathsf {F}}_1{\mathsf {L}}_3, \psi }\left( {\mathbb {F}}_q\right)&= q^2-3q+3 + H_q\left( \tfrac{1}{4}, \tfrac{1}{2}, \tfrac{3}{4}; 0,0,0 \,|\, \psi ^{-4}\right) + \\&\quad + 3qH_q\left( \tfrac{1}{14}, \tfrac{9}{14}, \tfrac{11}{14}; 0, \tfrac{1}{4}, \tfrac{3}{4} \,|\, \psi ^4\right) + 3qH_q\left( \tfrac{3}{14}, \tfrac{5}{14}, \tfrac{13}{14}; 0, \tfrac{1}{4}, \tfrac{3}{4} \,|\, \psi ^4\right) \\&\quad - 3\tfrac{1}{q} g\left( \tfrac{q^{\times }}{7}\right) g\left( \tfrac{4q^{\times }}{7}\right) g\left( \tfrac{2q^{\times }}{7}\right) + \left( 4q - 2\right) \\&= q^2 + q + 1+ H_q\left( \tfrac{1}{4}, \tfrac{1}{2}, \tfrac{3}{4}; 0,0,0 \,|\, \psi ^{-4}\right) \\&\quad + 3qH_q\left( \tfrac{1}{14}, \tfrac{9}{14}, \tfrac{11}{14}; 0, \tfrac{1}{4}, \tfrac{3}{4} \,|\, \psi ^4\right) + 3qH_q\left( \tfrac{3}{14}, \tfrac{5}{14}, \tfrac{13}{14}; 0, \tfrac{1}{4}, \tfrac{3}{4} \,|\, \psi ^4\right) \end{aligned} \end{aligned}$$

as desired. $$\square $$

### Remaining pencils

For the remaining three pencils ${\mathsf {F}}_2{\mathsf {L}}_2$, ${\mathsf {L}}_2{\mathsf {L}}_2$, and ${\mathsf {L}}_4$, the formula for the point counts can be derived in a similar manner. The details can be found in Appendix 4.8; we state here only the results.

#### Proposition 3.6.1

For *q* odd and $\psi \in {\mathbb {F}}_q^\times$, the following statements hold.

- If $$q\equiv 3\pmod 4$$, then
  $$\begin{aligned} \#X_{{\mathsf {F}}_2{\mathsf {L}}_2,\psi }\left( {\mathbb {F}}_q\right)&=q^2-q+1+H_q\left( \tfrac{1}{4},\tfrac{1}{2},\tfrac{3}{4};0,0,0\,|\,\psi ^{-4}\right) - q H_q\left( \tfrac{1}{4},\tfrac{3}{4};0,\tfrac{1}{2} \,|\,\psi ^{-4}\right) . \end{aligned}$$
- If $$q\equiv 5\pmod 8$$, then
  $$\begin{aligned} \#X_{{\mathsf {F}}_2{\mathsf {L}}_2,\psi }\left( {\mathbb {F}}_q\right)&=q^2-q+1+H_q\left( \tfrac{1}{4},\tfrac{1}{2},\tfrac{3}{4};0,0,0\,|\,\psi ^{-4}\right) \\&\quad +qH_q\left( \tfrac{1}{4},\tfrac{3}{4};0,\tfrac{1}{2} \,|\,\psi ^{-4}\right) -2qH_q\left( \tfrac{1}{2};0\,|\,\psi ^{-4}\right) . \end{aligned}$$
- If $$q\equiv 1\pmod 8$$, then
  $$\begin{aligned} \#X_{{\mathsf {F}}_2{\mathsf {L}}_2,\psi }\left( {\mathbb {F}}_q\right)&= q^2+7q +1 +H_q\left( \tfrac{1}{4},\tfrac{1}{2},\tfrac{3}{4};0,0,0\,|\,\psi ^{-4}\right) +qH_q\left( \tfrac{1}{4}, \tfrac{3}{4}; 0, \tfrac{1}{2} \,|\, \psi ^{-4}\right) \\&\quad + 2q H_q\left( \tfrac{1}{2}; 0 \,|\, \psi ^{-4}\right) + 2\omega \left( 2\right) ^{q^{\times }\,/4}qH_q\left( \tfrac{1}{8}, \tfrac{5}{8}; 0, \tfrac{1}{4} \,|\, \psi ^4\right) \\&\quad + 2\omega \left( 2\right) ^{q^{\times }\,/4}qH_q\left( \tfrac{3}{8}, \tfrac{7}{8}; 0, \tfrac{3}{4} \,|\, \psi ^4\right) . \end{aligned}$$

#### Proof

See Proposition B.2.1. $$\square $$

#### Proposition 3.6.2

For *q* odd and $\psi \in {\mathbb {F}}_q^\times$, the following statements hold.

- If $$q\equiv 3\pmod 4$$, then
  $$\begin{aligned} \#X_{{\mathsf {L}}_2{\mathsf {L}}_2,\psi }\left( {\mathbb {F}}_q\right) = q^2 + q+1 + H_q\left( \tfrac{1}{4}, \tfrac{1}{2}, \tfrac{3}{4}; 0,0,0 \,|\, \psi ^{-4}\right) - qH_q\left( \tfrac{1}{4}, \tfrac{3}{4}; 0, \tfrac{1}{2} \,|\, \psi ^{-4}\right) . \end{aligned}$$
- If $$q\equiv 1\pmod 4$$, then
  $$\begin{aligned} \#X_{{\mathsf {L}}_2{\mathsf {L}}_2,\psi }\left( {\mathbb {F}}_q\right)&= q^2+9q+1 + H_q\left( \tfrac{1}{4}, \tfrac{1}{2}, \tfrac{3}{4}; 0,0,0 \,|\, \psi ^{-4}\right) + qH_q\left( \tfrac{1}{4}, \tfrac{3}{4}; 0, \tfrac{1}{2} \,|\, \psi ^{-4}\right) \\&\quad + 2 \left( -1\right) ^{q^{\times }\,/4} \omega \left( \psi \right) ^{q^{\times }\,/2} q H_q\left( \tfrac{1}{8},\tfrac{3}{8},\tfrac{5}{8},\tfrac{7}{8};0,\tfrac{1}{4},\tfrac{1}{2},\tfrac{3}{4}\,|\,\psi ^{-4}\right) . \end{aligned}$$

#### Proof

See Proposition B.3.1. $$\square $$

#### Proposition 3.6.3

For *q* coprime to 10 and $\psi \in {\mathbb {F}}_q^\times$, the following statements hold.

- If $$q\not \equiv 1\pmod 5$$, then
  $$\begin{aligned} \#X_{{\mathsf {L}}_4,\psi }\left( {\mathbb {F}}_q\right) =q^2+3q+1+H_q\left( \tfrac{1}{4},\tfrac{1}{2},\tfrac{3}{4};0,0,0\,|\,\psi ^{-4}\right) . \end{aligned}$$
- If $$q\equiv 1 \pmod 5$$, then
  $$\begin{aligned} \#X_{{\mathsf {L}}_4,\psi }\left( {\mathbb {F}}_q\right)&=q^2+3q+1+H_q\left( \tfrac{1}{4},\tfrac{1}{2},\tfrac{3}{4};0,0,0\,|\,\psi ^{-4}\right) \\&\quad +\,4qH_q\left( \tfrac{1}{5},\tfrac{2}{5}, \tfrac{3}{5},\tfrac{4}{5} ;0, \tfrac{1}{4},\tfrac{1}{2}, \tfrac{3}{4}\,|\,\psi ^{4}\right) . \end{aligned}$$

#### Proof

See Proposition B.4.1. $$\square $$

## Proof of the main theorem and applications

In this section, we prove Main Theorem 1.4.1 by converting the hypergeometric point count formulas in the previous section into a global *L*-series. We conclude with some discussion and applications.

### From point counts to *L*-series

In this section, we define *L*-series of K3 surfaces and hypergeometric functions, setting up the notation we will use in the proof of our main theorem.

We begin with *L*-series of K3 surfaces. Let $\psi \in {\mathbb {Q}}{\smallsetminus } \{0,1\}$. Let $\diamond \in \{{\mathsf {F}}_4,{\mathsf {F}}_2{\mathsf {L}}_2,{\mathsf {F}}_1{\mathsf {L}}_3,{\mathsf {L}}_2{\mathsf {L}}_2,{\mathsf {L}}_4\}$ signify one of the five K3 families in (1.2.1). Let $S=S(\diamond ,\psi )$ be the set of bad primes in (1.2.1) together with the primes dividing the numerator or denominator of either $\psi ^4$ or $\psi ^4-1$.

#### Lemma 4.1.1

For $p \not \in S(\diamond ,\psi )$, the surface $X_{\diamond ,\psi }$ has good reduction at *p*.

#### Proof

Straightforward calculation. $$\square $$

Let $p \not \in S(\diamond ,\psi )$. The zeta function of $X_{\diamond ,\psi }$ over ${\mathbb {F}}_p$ is of the form

4.1.2 $$\begin{aligned} Z_p(X_{\diamond ,\psi },T) = \frac{1}{(1-T)(1-pT)P_{\diamond ,\psi ,p}(T)(1-p^2 T)} \end{aligned}$$

where $P_{\diamond ,\psi ,p}(T) \in 1+T{\mathbb {Z}}[T]$. The Hodge numbers of $X_{\diamond ,\psi }$ imply that the polynomial $P_{\diamond ,\psi ,p}(T)$ has degree 21. Equivalently, we have that

4.1.3 $$\begin{aligned} P_{\diamond ,\psi ,p}(T) = \det (1 - {{\,\mathrm{Frob}\,}}_p^{-1} T \,|\, H_{\acute{\hbox {e}}\text {t},\text {prim}}^2(X_{\diamond ,\psi },{\mathbb {Q}}_\ell )) \end{aligned}$$

is the characteristic polynomial of the Frobenius automorphism acting on primitive second-degree étale cohomology for $\ell \ne p$ (and independent of $\ell$). We then define the (incomplete) *L*-series

4.1.4 $$\begin{aligned} L_S(X_{\diamond ,\psi },s) :=\prod _{p \not \in S} P_{\diamond ,\psi ,p}(p^{-s})^{-1}, \end{aligned}$$

convergent for $s \in {\mathbb {C}}$ in a right half-plane by elementary estimates.

We now turn to hypergeometric *L*-series, recalling the definitions made in Sects. 3.1–3.2. Let $\varvec{\alpha },\varvec{\beta }$ be multisets of rational numbers that are disjoint modulo ${\mathbb {Z}}$. Let $t \in {\mathbb {Q}}{\smallsetminus } \{0,1\}$, and let $S(\varvec{\alpha },\varvec{\beta },t)$ be the set of primes dividing a denominator in $\varvec{\alpha } \cup \varvec{\beta }$ together with the primes dividing the numerator or denominator of either *t* or $t-1$.

Recall Definition 3.2.7 of the finite-field hypergeometric sums $H_{q}(\varvec{\alpha };\varvec{\beta }\,|\,t) \in K_{\varvec{\alpha },\varvec{\beta }} \subseteq {\mathbb {C}}$. For a prime power *q* such that $\varvec{\alpha },\varvec{\beta }$ is splittable, we define the formal series

4.1.5 $$\begin{aligned} L_q(H(\varvec{\alpha },\varvec{\beta }\,|\,t), T) :=\exp \biggl (-\sum _{r=1}^{\infty } H_{q^r}(\varvec{\alpha };\varvec{\beta }\,|\,t) \frac{T^r}{r} \biggr ) \in 1+TK_{\varvec{\alpha },\varvec{\beta }}[[T]] \end{aligned}$$

using Lemma 3.2.10(b). (Note the negative sign; below, this normalization will yield polynomials instead of inverse polynomials.)

For a number field *M*, a *prime* of *M* is a nonzero prime ideal of the ring of integers ${\mathbb {Z}}_M$ of *M*. We call a prime ${\mathfrak {p}}$ of *Mgood* (with respect to $\varvec{\alpha },\varvec{\beta },\psi$) if ${\mathfrak {p}}$ lies above a prime $p \not \in S(\varvec{\alpha },\varvec{\beta },\psi )$. Now, let *M* be an abelian extension of ${\mathbb {Q}}$ containing the field of definition $K :=K_{\varvec{\alpha },\varvec{\beta }}$ with the following property:

4.1.6 $$\begin{aligned} \text { for all good primes } {\mathfrak {p}}\text { of } M, \text { we have } q={{\,\mathrm{Nm}\,}}({\mathfrak {p}}) \text { splittable for } \varvec{\alpha },\varvec{\beta }. \end{aligned}$$

For example, if *m* is the least common multiple of all denominators in $\varvec{\alpha } \cup \varvec{\beta }$, then we may take $M={\mathbb {Q}}(\zeta _m)$. We will soon see that we will need to take *M* to be nontrivial extensions of *K* in Proposition 4.3.1 to deal with the splittable hypergeometric function given in Example 3.2.4. Let *m* be the conductor of *M*, i.e., the minimal positive integer such that $M \subseteq {\mathbb {Q}}(\zeta _m)$. Under the canonical identification $({\mathbb {Z}}/m{\mathbb {Z}})^\times \xrightarrow {\sim } {{\,\mathrm{Gal}\,}}({\mathbb {Q}}(\zeta _m)\,|\,{\mathbb {Q}})$ where $k \mapsto \sigma _k$ and $\sigma _k(\zeta _m)=\zeta _m^k$, let $H_M \le ({\mathbb {Z}}/m{\mathbb {Z}})^\times$ be such that ${{\,\mathrm{Gal}\,}}(M\,|\,{\mathbb {Q}}) \simeq ({\mathbb {Z}}/m{\mathbb {Z}})^\times /H_M$.

Now, let $p \not \in S(\varvec{\alpha },\varvec{\beta },\psi )$. Let ${\mathfrak {p}}_1,\dots ,{\mathfrak {p}}_r$ be the primes above *p* in *M*, and let $q=p^f={{\,\mathrm{Nm}\,}}({\mathfrak {p}}_i)$ for any *i*. Recall (by class field theory for ${\mathbb {Q}}$) that *f* is the order of *p* in $({\mathbb {Z}}/m{\mathbb {Z}})^\times /H_M$, and $rf=[M:{\mathbb {Q}}]$. Moreover, the set of primes $\{{\mathfrak {p}}_i\}_i$ arise as ${\mathfrak {p}}_i=\sigma _{k_i}({\mathfrak {p}}_1)$ where $k_i \in {\mathbb {Z}}$ are representatives of the quotient $({\mathbb {Z}}/m{\mathbb {Z}})^\times /\langle H_M, p \rangle$ of $({\mathbb {Z}}/m{\mathbb {Z}})^\times$ by the subgroup generated by $H_M$ and *p*. We then define

4.1.7 $$\begin{aligned} \begin{aligned} L_p(H(\varvec{\alpha },\varvec{\beta }\,|\,t), M, T)&:=\prod _{i=1}^r L_q(H(k_i\varvec{\alpha },k_i\varvec{\beta }\,|\,t),T^f) \\&= \prod _{k_i \in ({\mathbb {Z}}/m{\mathbb {Z}})^\times /\langle H_M, p \rangle } L_q(H(k_i\varvec{\alpha },k_i\varvec{\beta }\,|\,t),T^f) \in 1+TK[[T]]. \end{aligned} \end{aligned}$$

This product is well-defined up to choice of representatives $k_i$ of the cosets in $({\mathbb {Z}}/m{\mathbb {Z}})^\times /\langle H_M, p \rangle$. Indeed, by Lemma 3.2.10: part (c) gives

4.1.8 $$\begin{aligned} L_q(H(pk\varvec{\alpha },pk\varvec{\beta }\,|\,t),T^f)= L_q(H(k\varvec{\alpha },k\varvec{\beta }\,|\,t^p),T^f)= L_q(H(k\varvec{\alpha },k\varvec{\beta }\,|\,t),T^f) \end{aligned}$$

for all $k \in ({\mathbb {Z}}/m{\mathbb {Z}})^\times$ and all good primes *p*, since $t^p=t \in {\mathbb {F}}_p \subseteq {\mathbb {F}}_q$; and similarly part (a) implies it is well-defined for $k \in ({\mathbb {Z}}/m{\mathbb {Z}})^\times /H_M$ as $H_M \le H_K$.

#### Lemma 4.1.9

The following statements hold:

- We have
  4.1.10 $$\begin{aligned} L_p(H(\varvec{\alpha };\varvec{\beta }\,|\,t), M, T) \in 1+T {\mathbb {Q}}[[T]] \end{aligned}$$
  and
  4.1.11 $$\begin{aligned} L_p(H(\varvec{\alpha };\varvec{\beta }\,|\,t), M, T)=L_p(H(k\varvec{\alpha };k\varvec{\beta }\,|\,t), M, T) \end{aligned}$$
  for all $k \in {\mathbb {Z}}$ coprime to *p* and *m*.
- Let $H_K \le ({\mathbb {Z}}/m{\mathbb {Z}})^\times$ correspond to ${{\,\mathrm{Gal}\,}}(K\,|\,{\mathbb {Q}})$ as above, and let
  4.1.12 $$\begin{aligned} r(M|K,p) :=[\langle H_K, p\rangle :\langle H_M,p \rangle ]. \end{aligned}$$
  Then *r*(*M*|*K*, *p*) is the number of primes in *M* above a prime in *K* above *p*, and
  4.1.13 $$\begin{aligned} L_p(H(\varvec{\alpha },\varvec{\beta }\,|\,t), M, T) = \prod _{k_i \in ({\mathbb {Z}}/m{\mathbb {Z}})^\times /\langle H_K, p \rangle } L_q(H(k_i\varvec{\alpha },k_i\varvec{\beta }\,|\,t),T^f)^{r(M|K,p)}. \end{aligned}$$

#### Proof

For part (a), the descent to ${\mathbb {Q}}$ follows from Galois theory and Lemma 3.2.10; equality (4.1.11) follows as multiplication by *k* permutes the indices $k_i$ in $({\mathbb {Z}}/m{\mathbb {Z}})^\times /\langle H_M,p\rangle$. For part (b), the fact that *r*(*M*|*K*, *p*) counts the number of primes follows again from class field theory; to get (4.1.13), use Lemma 3.2.10 and the fact that the field of definition of $\varvec{\alpha },\varvec{\beta }$ is $K=K_{\varvec{\alpha },\varvec{\beta }}$. $\square$

We again package these together in an *L*-series:

4.1.14 $$\begin{aligned} L_S(H(\varvec{\alpha };\varvec{\beta }\,|\,t),M, s) :=\prod _{p \not \in S} L_p(H(\varvec{\alpha };\varvec{\beta }\,|\,t), M, p^{-s})^{-1}. \end{aligned}$$

We may expand (4.1.14) as a Dirichlet series

4.1.15 $$\begin{aligned} L_S(H(\varvec{\alpha };\varvec{\beta }\,|\,t),M, s) = \sum _{\begin{array}{c} {\mathfrak {n}}\subseteq {\mathbb {Z}}_M \\ {\mathfrak {n}}\ne (0) \end{array}} \frac{a_{{\mathfrak {n}}}}{{{\,\mathrm{Nm}\,}}({\mathfrak {n}})^s} \end{aligned}$$

with $a_{\mathfrak {n}}\in K=K_{\varvec{\alpha },\varvec{\beta }} \subset {\mathbb {C}}$, and again the series converges for $s \in {\mathbb {C}}$ in a right half-plane. If $M=K_{\varvec{\alpha },\varvec{\beta }}$, we suppress the notation *M* and write just $L_S(H(\varvec{\alpha };\varvec{\beta }\,|\,t),s)$, etc.

Finally, for a finite-order Dirichlet character $\chi$ over *M*, we let $L_S(H(\varvec{\alpha };\varvec{\beta }\,|\,t),M,s,\chi )$ denote the twist by $\chi$, defined by

4.1.16 $$\begin{aligned} L_S(H(\varvec{\alpha };\varvec{\beta }\,|\,t),M,s,\chi ) :=\sum _{\begin{array}{c} {\mathfrak {n}}\subseteq {\mathbb {Z}}_M \\ {\mathfrak {n}}\ne (0) \end{array}} \frac{\chi ({\mathfrak {n}}) a_{{\mathfrak {n}}}}{{{\,\mathrm{Nm}\,}}({\mathfrak {n}})^s}. \end{aligned}$$

### The Dwork pencil ${\mathsf {F}}_4$

In the remaining sections, we continue with the same notation: Let $t=\psi ^{-4}$ and let $S=S(\diamond ,\psi )$ be the set of bad primes in (1.2.1) together with the set of primes dividing the numerator or denominator of *t* or $t-1$. We now prove Main Theorem 1.4.1(a).

#### Proposition 4.2.1

Let $\psi \in {\mathbb {Q}}{\smallsetminus } \{0,1\}$, and let $t = \psi ^{-4}$. Then

$$\begin{aligned} L_S\left( X_{{\mathsf {F}}_4,\psi }, s\right)&= L_S\left( H\left( \tfrac{1}{4}, \tfrac{1}{2}, \tfrac{3}{4}; 0, 0, 0\,|\, t\right) , s\right) \\&\quad \cdot L_S\left( H\left( \tfrac{1}{4}, \tfrac{3}{4}; 0, \tfrac{1}{2} \,|\, t\right) , s-1, \phi _{-1}\right) ^3 \\&\quad \cdot L_S\left( H\left( \tfrac{1}{2}; 0 \,|\, t\right) , {\mathbb {Q}}\left( \sqrt{-1}\right) , s-1, \phi _{\sqrt{-1}}\right) ^6, \end{aligned}$$

where

4.2.2 $$\begin{aligned} \begin{aligned} \phi _{-1}\left( p\right)&=\biggl (\displaystyle {\frac{-1}{p}}\biggr ) = \left( -1\right) ^{\left( p-1\right) /2}&\text { is associated to } {\mathbb {Q}}\left( \sqrt{-1}\right) \,|\, {\mathbb {Q}}, \text { and } \\ \phi _{\sqrt{-1}}\left( {\mathfrak {p}}\right)&=\biggl (\displaystyle {\frac{\sqrt{-1}}{{\mathfrak {p}}}}\biggr )=\left( -1\right) ^{\left( {{\,\mathrm{Nm}\,}}\left( {\mathfrak {p}}\right) -1\right) /4}&\text { is associated to } {\mathbb {Q}}\left( \zeta _8\right) \,|\,{\mathbb {Q}}\left( \sqrt{-1}\right) . \end{aligned} \end{aligned}$$

#### Proof

Recall Proposition 3.4.1, where we wrote the number of ${\mathbb {F}}_q$ points on ${\mathsf {F}}_4$ in terms of the finite-field hypergeometric functions. We rewrite these for convenience:

4.2.3 $$\begin{aligned} \begin{aligned} \#X_{{\mathsf {F}}_4,\psi }\left( {\mathbb {F}}_q\right)&=q^2+q+1+H_q\left( \tfrac{1}{4},\tfrac{1}{2},\tfrac{3}{4};0,0,0\,|\,t\right) +\phi _{-1}\left( q\right) 3qH_q\left( \tfrac{1}{4},\tfrac{3}{4};0, \tfrac{1}{2}\,|\,t\right) \\&\quad +\delta [q\equiv 1~({\text {mod}}~{4})]12\phi _{\sqrt{-1}}\left( q\right) qH_q\left( \tfrac{1}{2};0\,|\,t\right) , \end{aligned} \end{aligned}$$

where $\delta [{\mathcal {P}}]=1,0$ according as if ${\mathcal {P}}$ holds or not.

Each summand in (4.2.3) corresponds to a multiplicative term in the exponential generating series. The summand $q^2+q+1$ gives the factor $(1-T)(1-qT)(1-q^2T)$ in the denominator of (4.1.2), so $L_S(X_{{\mathsf {F}}_4,\psi }, s)$ represents the rest of the sum. The summand $H_q(\tfrac{1}{4}, \tfrac{1}{2}, \tfrac{3}{4};0,0,0\,|\,t)$ yields $L_S( H(\tfrac{1}{4}, \tfrac{1}{2}, \tfrac{3}{4}; 0, 0, 0\,|\, t), s)$ by definition.

Next, we consider the summand $\phi _{-1}(q)3qH_q(\tfrac{1}{4},\tfrac{3}{4};0, \tfrac{1}{2}\,|\,t)$: For each $p \not \in S$, we have

4.2.4 $$\begin{aligned} \begin{aligned}&\exp \left( -\sum _{r=1}^\infty \phi _{-1}\left( p^r\right) 3p^rH_{p^r}\left( \tfrac{1}{4},\tfrac{3}{4};0, \tfrac{1}{2}\,|\,t\right) \frac{\left( p^{-s}\right) ^r}{r}\right) \\&\quad = \exp \left( - \sum _{r=1}^\infty \phi _{-1}\left( p^r\right) H_{p^r}\left( \tfrac{1}{4}, \tfrac{3}{4}; 0, \tfrac{1}{2} \,|\, t\right) \frac{p^{\left( 1-s\right) r}}{r}\right) ^{3} \\&\quad = L_{p}\left( H_p\left( \tfrac{1}{4}, \tfrac{3}{4}; 0, \tfrac{1}{2} \,|\, t\right) , p^{1-s}, \phi _{-1}\right) ^{3}. \end{aligned} \end{aligned}$$

Combining these for all $p \not \in S$ then gives the *L*-series $L_S( H(\tfrac{1}{4}, \tfrac{3}{4}; 0, \tfrac{1}{2} \,|\, t), s-1, \phi _{-1})^3$.

We conclude with the final term $12\phi _{\sqrt{-1}}(q)qH_q(\tfrac{1}{2};0\,|\,t)$ which exists only when $q \equiv 1 \pmod 4$. We accordingly consider two cases. First, if $p \equiv 1 \pmod 4$, then in ${\mathbb {Z}}[\sqrt{-1}]$, the two primes ${\mathfrak {p}}_1,{\mathfrak {p}}_2$ above *p* have norm *p*. We compute

4.2.5 $$\begin{aligned} \begin{aligned}&\exp \left( -\sum _{r=1}^\infty 12\phi _{\sqrt{-1}}\left( p^r\right) p^rH_{p^r}\left( \tfrac{1}{2};0\,|\,t\right) \frac{\left( p^{-s}\right) ^r}{r}\right) \\&\quad = L_p\left( H\left( \tfrac{1}{2};0\,|\,t\right) , p^{1-s}, \phi _{\sqrt{-1}}\right) ^{12}\\&\quad = L_p\left( H\left( \tfrac{1}{2};0\,|\,t\right) , p^{1-s}, \phi _{\sqrt{-1}}\right) ^{6}L_p\left( H\left( -\tfrac{1}{2};0\,|\,t\right) , p^{1-s}, \phi _{\sqrt{-1}}\right) ^{6},\\&\quad = L_p\left( H\left( \tfrac{1}{2}; 0 \,|\, t\right) , {\mathbb {Q}}\left( \sqrt{-1}\right) , p^{1-s}, \phi _{\sqrt{-1}}\right) ^6 \end{aligned} \end{aligned}$$

where the second equality holds because the definition of the hypergeometric sum only depends on parameters modulo ${\mathbb {Z}}$ and the final equality is using Definition (4.1.7) and using that ${{\,\mathrm{Gal}\,}}(M\,|\,{\mathbb {Q}})$ when $M = {\mathbb {Q}}(\sqrt{-1})$ is generated by complex conjugation.

Second, if $p \equiv 3 \pmod 4$, then there is a unique prime ideal ${\mathfrak {p}}$ above *p* with norm ${{\,\mathrm{Nm}\,}}({\mathfrak {p}}) = p^2$, and

4.2.6 $$\begin{aligned} \begin{aligned}&\exp \left( -\sum _{r=1}^\infty 12\phi _{\sqrt{-1}}\left( p^{2r}\right) p^{2r}H_{p^{2r}}\left( \tfrac{1}{2};0\,|\,t\right) \frac{\left( p^{-s}\right) ^{2r}}{2r}\right) \\&\quad = L_{p^2}\left( H\left( \tfrac{1}{2};0\,|\,t\right) , p^{2\left( 1-s\right) }, \phi _{\sqrt{-1}}\right) ^{6}\\&\quad = L_{p}\left( H\left( \tfrac{1}{2};0\,|\,t\right) , {\mathbb {Q}}\left( \sqrt{-1}\right) , p^{1-s},\phi _{\sqrt{-1}}\right) ^6. \end{aligned} \end{aligned}$$

Taking the product of (4.2.5) and (4.2.6) over all prescribed *p*, we obtain the last *L*-series factor. $\square$

### The Klein–Mukai pencil ${\mathsf {F}}_1{\mathsf {L}}_3$

We now prove Theorem 1.4.1(b).

#### Proposition 4.3.1

For the Klein–Mukai pencil ${\mathsf {F}}_1 {\mathsf {L}}_3$,

$$\begin{aligned} L_S\left( X_{{\mathsf {F}}_1{\mathsf {L}}_3,\psi }, s\right)&= L_S\left( H\left( \tfrac{1}{4}, \tfrac{1}{2}, \tfrac{3}{4}; 0, 0, 0\,|\, t\right) , s\right) \\&\quad \cdot L_S\left( H\left( \tfrac{1}{14}, \tfrac{9}{14}, \tfrac{11}{14}; 0, \tfrac{1}{4}, \tfrac{3}{4} \,|\, t^{-1}\right) , {\mathbb {Q}}\left( \zeta _7\right) , s-1\right) \end{aligned}$$

where for $\varvec{\alpha },\varvec{\beta }=\{\tfrac{1}{14}, \tfrac{9}{14}, \tfrac{11}{14}\},\{0, \tfrac{1}{4}, \tfrac{3}{4}\}$ we have field of definition $K_{\varvec{\alpha },\varvec{\beta }}={\mathbb {Q}}(\sqrt{-7})$.

#### Remark 4.3.2

By Lemma 4.1.9, we have

$$\begin{aligned} L_S\left( H\left( \tfrac{1}{14}, \tfrac{9}{14}, \tfrac{11}{14}; 0, \tfrac{1}{4}, \tfrac{3}{4} \,|\, t^{-1}\right) , s\right) = L_S\left( H\left( \tfrac{3}{14}, \tfrac{5}{14}, \tfrac{13}{14}; 0, \tfrac{1}{4}, \tfrac{3}{4} \,|\, t^{-1}\right) , s\right) . \end{aligned}$$

#### Proof

Recall that by Proposition 3.5.1, we have

$$\begin{aligned} \begin{aligned} \#X_{{\mathsf {F}}_1{\mathsf {L}}_3,\psi }\left( {\mathbb {F}}_q\right)&=q^2+q+1+H_q\left( \tfrac{1}{4},\tfrac{1}{2},\tfrac{3}{4};0,0,0\,|\,t\right) \\&\quad +3q\delta [q \equiv 1 ~({\text {mod}}~{7})]\left( H_q\left( \tfrac{1}{14},\tfrac{9}{14},\tfrac{11}{14};0,\tfrac{1}{4},\tfrac{3}{4}\,|\,t^{-1}\right) \right. \\&\quad \left. +\,H_q\left( \tfrac{3}{14},\tfrac{5}{14},\tfrac{13}{14};0,\tfrac{1}{4},\tfrac{3}{4}\,|\,t^{-1}\right) \right) . \end{aligned} \end{aligned}$$

We compute that the field of definition (see Definition 3.1.10) associated to the parameters $\varvec{\alpha },\varvec{\beta }=\{\tfrac{1}{14}, \tfrac{9}{14}, \tfrac{11}{14}\},\{0, \tfrac{1}{4}, \tfrac{3}{4}\}$ and to $\varvec{\alpha },\varvec{\beta }=\{\tfrac{3}{14}, \tfrac{5}{14}, \tfrac{13}{14}\},\{0, \tfrac{1}{4}, \tfrac{3}{4}\}$ is ${\mathbb {Q}}(\sqrt{-7})$. We take $M={\mathbb {Q}}(\zeta _7)$ and consider a prime ${\mathfrak {p}}$ of *M*, and let $q={{\,\mathrm{Nm}\,}}({\mathfrak {p}})$. Then $q \equiv 1 \pmod {7}$, and in Example 3.2.4, we have seen that *q* is splittable for $\varvec{\alpha },\varvec{\beta }$.

We proceed in two cases, according to the splitting behavior of *p* in $K=K_{\varvec{\alpha },\varvec{\beta }}={\mathbb {Q}}(\sqrt{-7})$. First, suppose that $p\equiv 1,2,4 \pmod 7$, or equivalently *p* splits in *K*. We have $2r(M|K,p)f=6$ so $r(M|K,p)=3$. We then apply Lemma 4.1.9(b), with $({\mathbb {Z}}/m{\mathbb {Z}})^\times / \langle H_K, p\rangle = \{\pm 1\}$, to obtain

4.3.3 $$\begin{aligned}&\exp \left( -\sum _{r=1}^\infty 3p^{fr}H_{p^{fr}}\left( \tfrac{1}{14},\tfrac{9}{14},\tfrac{11}{14};0,\tfrac{1}{4},\tfrac{3}{4}\,|\,t\right) \frac{\left( p^{-s}\right) ^{fr}}{fr} \right. \nonumber \\&\qquad \left. -3p^{fr}H_{p^{fr}}\left( \tfrac{3}{14},\tfrac{5}{14},\tfrac{13}{14};0,\tfrac{1}{4},\tfrac{3}{4}\,|\,t\right) \frac{\left( p^{-s}\right) ^{fr}}{fr} \right) \nonumber \\&\quad = L_{p^f}\left( H\left( \tfrac{1}{14},\tfrac{9}{14},\tfrac{11}{14};0,\tfrac{1}{4},\tfrac{3}{4}\,|\,t\right) , p^{1-s}\right) ^{3/f} L_{p^f}\left( H\left( \tfrac{3}{14},\tfrac{5}{14},\tfrac{13}{14};0,\tfrac{1}{4},\tfrac{3}{4}\,|\,t\right) , p^{1-s}\right) ^{3/f} \nonumber \\&\quad = \prod _{k_i \in \left( {\mathbb {Z}}/m{\mathbb {Z}}\right) ^\times / \langle H_K, p\rangle } L_p\left( H\left( \tfrac{1}{14}k_i,\tfrac{9}{14}k_i,\tfrac{11}{14}k_i;0,\tfrac{1}{4}k_i,\tfrac{3}{4}k_i\,|\,t\right) , p^{1-s}\right) ^{r\left( M|K,p\right) } \nonumber \\&\quad = L_p\left( H\left( \tfrac{1}{14},\tfrac{9}{14},\tfrac{11}{14};0,\tfrac{1}{4},\tfrac{3}{4}\,|\,t\right) , {\mathbb {Q}}\left( \zeta _7\right) , p^{1-s}\right) . \end{aligned}$$

To conclude, suppose $p \equiv 3,5,6 \pmod {7}$, i.e., *p* is inert in *K*. Now, $({\mathbb {Z}}/m{\mathbb {Z}})^\times / \langle H_K, p\rangle = \{1\}$ and $6 = r(M|K, p)f$. By Lemma 3.2.10(c), for all $q \equiv 1 \pmod 7$, we have that

4.3.4 $$\begin{aligned} H_{q}\left( \tfrac{1}{14},\tfrac{9}{14},\tfrac{11}{14};0,\tfrac{1}{4},\tfrac{3}{4}\,|\,t\right)= & {} H_{q}\left( \tfrac{1}{14}p,\tfrac{9}{14}p,\tfrac{11}{14}p;0,-\tfrac{1}{4},-\tfrac{3}{4}\,|\,t^p\right) \nonumber \\= & {} H_{q}\left( \tfrac{3}{14},\tfrac{5}{14},\tfrac{13}{14};0,\tfrac{1}{4},\tfrac{3}{4}\,|\,t\right) . \end{aligned}$$

Using the previous line and Lemma 4.1.9(b),

4.3.5 $$\begin{aligned}&\exp \left( -\sum _{r=1}^\infty 3p^{fr}H_{p^{fr}}\left( \tfrac{1}{14},\tfrac{9}{14},\tfrac{11}{14};0,\tfrac{1}{4},\tfrac{3}{4}\,|\,t\right) \frac{\left( p^{-s}\right) ^{fr}}{fr} \right. \nonumber \\&\qquad \left. +3p^{fr}H_{p^{fr}}\left( \tfrac{3}{14},\tfrac{5}{14},\tfrac{13}{14};0,\tfrac{1}{4},\tfrac{3}{4}\,|\,t\right) \frac{\left( p^{-s}\right) ^{fr}}{fr} \right) \nonumber \\&\quad = \exp \left( -\sum _{r=1}^\infty 6H_{p^{fr}}\left( \tfrac{1}{14},\tfrac{9}{14},\tfrac{11}{14};0,\tfrac{1}{4},\tfrac{3}{4}\,|\,t\right) \frac{\left( p^{1-s}\right) ^{fr}}{fr}\right) \nonumber \\&\quad = L_{p^f}\left( H\left( \tfrac{1}{14},\tfrac{9}{14},\tfrac{11}{14};0,\tfrac{1}{4},\tfrac{3}{4}\,|\,t\right) , p^{1-s}\right) ^{6/f} \nonumber \\&\quad = L_{p^f}\left( H\left( \tfrac{1}{14},\tfrac{9}{14},\tfrac{11}{14};0,\tfrac{1}{4},\tfrac{3}{4}\,|\,t\right) , p^{1-s}\right) ^{r\left( M|K, p\right) } \nonumber \\&\quad = L_p\left( H\left( \tfrac{1}{14},\tfrac{9}{14},\tfrac{11}{14};0,\tfrac{1}{4},\tfrac{3}{4}\,|\,t\right) , {\mathbb {Q}}\left( \zeta _7\right) , p^{1-s}\right) . \end{aligned}$$

$$\square $$

### The pencil ${\mathsf {F}}_2{\mathsf {L}}_2$

We now prove Theorem 1.4.1(c):

#### Proposition 4.4.1

For the pencil ${\mathsf {F}}_2 {\mathsf {L}}_2$,

$$\begin{aligned} L_S\left( X_{{\mathsf {F}}_2{\mathsf {L}}_2,\psi }, s\right)&= L_S\left( H\left( \tfrac{1}{4}, \tfrac{1}{2}, \tfrac{3}{4}; 0, 0, 0\,|\, t\right) , s\right) \\&\quad \cdot L_S\left( {\mathbb {Q}}\left( \zeta _8\right) \,|\,{\mathbb {Q}},s-1\right) ^2 \\&\quad \cdot L_S\left( H\left( \tfrac{1}{4}, \tfrac{3}{4}; 0, \tfrac{1}{2} \,|\, t\right) , s-1, \phi _{-1}\right) \\&\quad \cdot L_S\left( H\left( \tfrac{1}{2};0 \,|\, t\right) , {\mathbb {Q}}\left( \sqrt{-1}\right) , s-1, \phi _{\sqrt{-1}}\right) \\&\quad \cdot L_S\left( H\left( \tfrac{1}{8}, \tfrac{5}{8}; 0, \tfrac{1}{4} \,|\, t^{-1}\right) , {\mathbb {Q}}\left( \zeta _8\right) , s-1, \phi _{\sqrt{2}}\right) \end{aligned}$$

where

- the character $\phi _{\sqrt{-1}}$ is defined in (4.2.2),
- for $\varvec{\alpha },\varvec{\beta }=\{\tfrac{1}{8}, \tfrac{5}{8}\},\{0, \tfrac{1}{4}\}$ we have the field of definition $K_{\varvec{\alpha },\varvec{\beta }}={\mathbb {Q}}(\sqrt{-1})$, $M = {\mathbb {Q}}(\zeta _8)$, and
  $$\begin{aligned} \phi _{\sqrt{2}}({\mathfrak {p}}) :=\biggl (\displaystyle {\frac{\sqrt{2}}{{\mathfrak {p}}}}\biggr ) \equiv 2^{({{\,\mathrm{Nm}\,}}({\mathfrak {p}})-1)/4} ~({\text {mod}}~{{\mathfrak {p}}}) \text { is associated to } {\mathbb {Q}}(\zeta _8,\root 4 \of {2}) \,|\, {\mathbb {Q}}(\zeta _8), \text {and} \end{aligned}$$
- $L({\mathbb {Q}}(\zeta _8)\,|\,{\mathbb {Q}},s) = \zeta _{{\mathbb {Q}}(\zeta _8)}(s)/\zeta (s)$, where $\zeta _{{\mathbb {Q}}(\zeta _8)}(s)$ is the Dedekind zeta function of ${\mathbb {Q}}(\zeta _8)$ and $\zeta (s)=\zeta _{\mathbb {Q}}(s)$ the Riemann zeta function.

#### Proof

We now appeal to Proposition 3.6.1, which we summarize as:

4.4.2 $$\begin{aligned} \begin{aligned} \#X_{{\mathsf {F}}_2{\mathsf {L}}_2,\psi }\left( {\mathbb {F}}_q\right)&= q^2 + q + 1 + 2q\cdot {\left\{ \begin{array}{ll} 3 &{}\quad \text { if } q \equiv 1 \pmod 8 \\ -1 &{}\quad \text { if } q \not \equiv 1 \pmod 8\end{array}\right. } \\&\quad + H_q\left( \tfrac{1}{4},\tfrac{1}{2},\tfrac{3}{4};0,0,0\,|\,t\right) + \phi _{-1}\left( q\right) qH_q\left( \tfrac{1}{4},\tfrac{3}{4};0,\tfrac{1}{2} \,|\,t\right) \\&\quad + 2\phi _{\sqrt{-1}}\left( q\right) q\delta [q\equiv 1 ~({\text {mod}}~{4})]H_q\left( \tfrac{1}{2}; 0 \,|\, t\right) \\&\quad + 2 \phi _{\sqrt{2}}\left( q\right) q\delta [q\equiv 1 ~({\text {mod}}~{8})]\left( H_q\left( \tfrac{1}{8}, \tfrac{5}{8}; 0, \tfrac{1}{4} \,|\, t^{-1}\right) \right. \\&\quad \left. + H_q\left( \tfrac{3}{8}, \tfrac{7}{8}; 0, \tfrac{3}{4} \,|\, t^{-1}\right) \right) . \end{aligned} \end{aligned}$$

For the new sum with parameters $\varvec{\alpha },\varvec{\beta }=\{\tfrac{1}{8}, \tfrac{5}{8}\},\{0, \tfrac{1}{4}\}$, we have field of definition $K_{\varvec{\alpha },\varvec{\beta }}={\mathbb {Q}}(\sqrt{-1})$ because the subgroup of $({\mathbb {Z}}/8{\mathbb {Z}})^\times$ preserving these subsets is generated by 5.

The term $q^2+q+1$ in (4.4.2) is handled as before. For the next term, by splitting behavior in the biquadratic field ${\mathbb {Q}}(\zeta _8)={\mathbb {Q}}(\sqrt{-1},\sqrt{2})$ we obtain

4.4.3 $$\begin{aligned} L_p({\mathbb {Q}}(\zeta _8)\,|\,{\mathbb {Q}},pT) = {\left\{ \begin{array}{ll} \left( 1-pT\right) ^3 &{}\quad \text { if } p \equiv 1 ~({\text {mod}}~{8}), \\ \displaystyle {\frac{\left( 1-\left( pT\right) ^2\right) ^2}{1-pT}=\left( 1-pT\right) \left( 1+pT\right) ^2} &{}\quad \text { if } p \not \equiv 1 ~({\text {mod}}~{8}). \end{array}\right. } \end{aligned}$$

For $q \equiv 1 \pmod {8}$, the contribution to the exponential generating series is 3*q*, otherwise the contribution is $q-2q=-q$.

All remaining terms except for the term

$$\begin{aligned} 2\phi _{\sqrt{2}}\left( q\right) q\delta [q\equiv 1 ~({\text {mod}}~{8})]\left( H_q\left( \tfrac{1}{8}, \tfrac{5}{8}; 0, \tfrac{1}{4} \,|\, t^{-1}\right) + H_q\left( \tfrac{3}{8}, \tfrac{7}{8}; 0, \tfrac{3}{4} \,|\, t^{-1}\right) \right) \end{aligned}$$

are handled in the proof of Proposition 4.2.1. We choose $M = {\mathbb {Q}}(\zeta _8)$ which has conductor $m=8$. Then $H_K = \langle 5\rangle \le ({\mathbb {Z}}/8{\mathbb {Z}})^\times$. Let $\epsilon =\biggl (\displaystyle {\frac{\sqrt{2}}{{\mathfrak {p}}}}\biggr )$ for a prime ${\mathfrak {p}}$ above *p* in ${\mathbb {Q}}(\zeta _8)$ (and independent of this choice).

Suppose that $p \equiv 1 \pmod 8$. We compute that $f=1$ and $r(M|K,p) = 2$. By applying Lemma 4.1.9(b), we have:

4.4.4 $$\begin{aligned}&\exp \left( -\sum _{r=1}^\infty 2 \epsilon ^r p^{r}H_{p^{r}}\left( \tfrac{1}{8},\tfrac{5}{8};0,\tfrac{1}{4}\,|\,t^{-1}\right) \frac{\left( p^{-s}\right) ^{r}}{r} - \sum _{r=1}^\infty 2 \epsilon ^r p^r H_{p^{r}}\left( \tfrac{3}{8},\tfrac{7}{8};0,\tfrac{3}{4}\,|\,t^{-1}\right) \frac{\left( p^{-s}\right) ^{r}}{r} \right) \nonumber \\&\quad = L_p\left( H\left( \tfrac{1}{8},\tfrac{5}{8};0,\tfrac{1}{4}\,|\,t^{-1}\right) ,p^{1-s}, \phi _{\sqrt{2}}\right) ^2L_p\left( H_{p^{r}}\left( \tfrac{3}{8},\tfrac{7}{8};0,\tfrac{3}{4}\,|\,t^{-1}\right) , p^{1-s}, \phi _{\sqrt{2}}\right) ^2 \nonumber \\&\quad = \prod _{k \in \left( {\mathbb {Z}}/ 8{\mathbb {Z}}\right) ^\times / \langle H_K, p\rangle } L_p\left( H\left( \tfrac{1}{8}k,\tfrac{5}{8}k;0,\tfrac{1}{4}k\,|\,t^{-1}\right) ,p^{1-s}, \phi _{\sqrt{2}}\right) ^2 \nonumber \\&\quad = L_p\left( H\left( \tfrac{1}{8},\tfrac{5}{8};0,\tfrac{1}{4}\,|\,t^{-1}\right) , {\mathbb {Q}}\left( \zeta _8\right) ,p^{1-s}, \phi _{\sqrt{2}}\right) . \end{aligned}$$

Suppose now that $p\equiv 3,7 \pmod 8$. Then $f = 2$ and $r(M|K,p) = 2$. By Lemma 3.2.10(c), for all *q* a power of *p* so that $q \equiv 1 \pmod 8$, we have that

4.4.5 $$\begin{aligned} H_{q}\left( \tfrac{1}{8},\tfrac{5}{8};0,\tfrac{1}{4}\,|\,t^{-1}\right) = H_{q}\left( \tfrac{1}{8}p,\tfrac{5}{8}p;0,\tfrac{1}{4}p\,|\,t^{-p}\right) = H_{q}\left( \tfrac{3}{8},\tfrac{7}{8};0,\tfrac{3}{4}\,|\,t^{-1}\right) . \end{aligned}$$

Again applying Lemma 4.1.9(b), we have:

4.4.6 $$\begin{aligned}&\exp \left( -\sum _{r=1}^\infty 2\epsilon ^r p^{2r}H_{p^{2r}}\left( \tfrac{1}{8},\tfrac{5}{8};0,\tfrac{1}{4}\,|\,t^{-1}\right) \frac{\left( p^{-s}\right) ^{2r}}{2r} \right. \nonumber \\&\qquad \left. - \sum _{r=1}^\infty 2\epsilon ^r p^{2r}H_{p^{2r}}\left( \tfrac{3}{8},\tfrac{7}{8};0,\tfrac{3}{4}\,|\,t^{-1}\right) \frac{\left( p^{-s}\right) ^{2r}}{2r} \right) \nonumber \\&\quad =\exp \left( -\sum _{r=1}^\infty 2\epsilon ^rp^{2r}H_{p^{2r}}\left( \tfrac{1}{8},\tfrac{5}{8};0,\tfrac{1}{4}\,|\,t^{-1}\right) \frac{\left( p^{-s}\right) ^{2r}}{r}\right) \nonumber \\&\quad = L_{p^2}\left( H\left( \tfrac{1}{8},\tfrac{5}{8};0,\tfrac{1}{4}\,|\,t^{-1}\right) ,p^{1-s}, \phi _{\sqrt{2}}\right) ^2 \nonumber \\&\quad = L_{p^2}\left( H\left( \tfrac{1}{8},\tfrac{5}{8};0,\tfrac{1}{4}\,|\,t^{-1}\right) ,p^{1-s}, \phi _{\sqrt{2}}\right) ^{r\left( M|K,p\right) } \nonumber \\&\quad = L_{p^2}\left( H\left( \tfrac{1}{8},\tfrac{5}{8};0,\tfrac{1}{4}\,|\,t^{-1}\right) , {\mathbb {Q}}\left( \zeta _8\right) ,p^{1-s}, \phi _{\sqrt{2}}\right) . \end{aligned}$$

Finally, suppose that $p \equiv 5 \pmod 8$. Then $f=2$ and $r(M|K, p) = 1$, and now

4.4.7 $$\begin{aligned}&\exp \left( -\sum _{r=1}^\infty 2\epsilon ^rp^{2r}H_{p^{2r}}\left( \tfrac{1}{8},\tfrac{5}{8};0,\tfrac{1}{4}\,|\,t^{-1}\right) \frac{\left( p^{-s}\right) ^{2r}}{2r}\right. \nonumber \\&\qquad \left. - \sum _{r=1}^\infty 2\epsilon ^rp^{2r}H_{p^{2r}}\left( \tfrac{3}{8},\tfrac{7}{8};0,\tfrac{3}{4}\,|\,t^{-1}\right) \frac{\left( p^{-s}\right) ^{2r}}{2r} \right) \nonumber \\&\quad = L_{p^2}\left( H\left( \tfrac{1}{8},\tfrac{5}{8};0,\tfrac{1}{4}\,|\,t^{-1}\right) ,p^{1-s}, \phi _{\sqrt{2}}\right) L_{p^2}\left( H\left( \tfrac{3}{8},\tfrac{7}{8};0,\tfrac{3}{4}\,|\,t^{-1}\right) ,p^{1-s}, \phi _{\sqrt{2}}\right) \nonumber \\&\quad = \prod _{k\in \left( {\mathbb {Z}}/ 8{\mathbb {Z}}\right) ^\times / \langle H_K, p\rangle } L_{p^2}\left( H\left( \tfrac{1}{8}k,\tfrac{5}{8}k;0,\tfrac{1}{4}k\,|\,t^{-1}\right) ,p^{1-s}, \phi _{\sqrt{2}}\right) \nonumber \\&\quad = L_p\left( H\left( \tfrac{1}{8},\tfrac{5}{8};0,\tfrac{1}{4}\,|\,t^{-1}\right) , {\mathbb {Q}}\left( \zeta _8\right) ,p^{1-s}, \phi _{\sqrt{2}}\right) . \end{aligned}$$

$$\square $$

### The pencil ${\mathsf {L}}_2{\mathsf {L}}_2$

We now prove Theorem 1.4.1(d).

#### Proposition 4.5.1

For the pencil ${\mathsf {L}}_2 {\mathsf {L}}_2$, we have

$$\begin{aligned} L_S\left( X_{{\mathsf {L}}_2{\mathsf {L}}_2,\psi }, s\right)&= L_S\left( H\left( \tfrac{1}{4}, \tfrac{1}{2}, \tfrac{3}{4}; 0, 0, 0\,|\, t\right) , s\right) \\&\quad \cdot \zeta _{{\mathbb {Q}}\left( \sqrt{-1}\right) }\left( s-1\right) ^4 \\&\quad \cdot L_S\left( H\left( \tfrac{1}{4}, \tfrac{3}{4}; 0, \tfrac{1}{2} \,|\, t\right) , s-1, \phi _{-1}\right) \\&\quad \cdot L_S\left( H\left( \tfrac{1}{8}, \tfrac{3}{8}, \tfrac{5}{8}, \tfrac{7}{8}; 0, \tfrac{1}{4}, \tfrac{1}{2}, \tfrac{3}{4}\,|\, t\right) , {\mathbb {Q}}\left( i\right) , s-1, \phi _{\sqrt{-1}}\phi _{\psi }\right) , \end{aligned}$$

where the characters $\phi _{-1},\phi _{\sqrt{-1}}$ are defined in (4.2.2) and

4.5.2 $$\begin{aligned} \begin{aligned} \phi _{\psi }(p)&=\biggl (\displaystyle {\frac{\psi }{p}}\biggr )&\text { is associated to } {\mathbb {Q}}(\sqrt{\psi })\,|\,{\mathbb {Q}}\end{aligned} \end{aligned}$$

and $\zeta _{{\mathbb {Q}}(\sqrt{-1})}(s)$ is the Dedekind zeta function of ${\mathbb {Q}}(\sqrt{-1})$.

#### Proof

By Proposition 3.6.2, we have the point counts

4.5.3 $$\begin{aligned} \begin{aligned} \#X_{{\mathsf {L}}_2{\mathsf {L}}_2,\psi }\left( {\mathbb {F}}_q\right)&= q^2 + q+1 + 4q\cdot {\left\{ \begin{array}{ll} 2 &{}\quad \text { if } q \equiv 1 \pmod 4, \\ 0 &{} \quad \text { if } q \equiv 3 \pmod 4\end{array}\right. } \\&\quad + H_q\left( \tfrac{1}{4}, \tfrac{1}{2}, \tfrac{3}{4}; 0,0,0 \,|\, t\right) +\left( -1\right) ^{q^{\times }\,/2}qH_q\left( \tfrac{1}{4}, \tfrac{3}{4}; 0, \tfrac{1}{2} \,|\, t\right) \\&\quad + 2 \left( -1\right) ^{q^{\times }\,/4} \omega \left( \psi \right) ^{q^{\times }\,/2} q\delta [q\equiv 1~({\text {mod}}~{4})] H_q\left( \tfrac{1}{8},\tfrac{3}{8},\tfrac{5}{8},\tfrac{7}{8};0,\tfrac{1}{4},\tfrac{1}{2},\tfrac{3}{4}\,|\,t\right) . \end{aligned} \end{aligned}$$

Again by splitting behavior, we have

4.5.4 $$\begin{aligned} \zeta _{{\mathbb {Q}}(i),p}(pT) = {\left\{ \begin{array}{ll} (1-pT)^2 &{}\quad \text { if } p \equiv 1 ~({\text {mod}}~{4}), \\ 1-p^2T^2 &{}\quad \text { if } p \equiv 3 ~({\text {mod}}~{4}). \end{array}\right. } \end{aligned}$$

For $q \equiv 1 \pmod {4}$, the contribution to the exponential generating series is 2*q*, otherwise the contribution is 0.

All, but the last summand have been identified in the previous propositions, and this one follows in a similar, but easier manner (because it has ${\mathbb {Q}}$ as field of definition) applying Lemma 4.1.9(b), with $M={\mathbb {Q}}(\sqrt{-1})$ and $fr(M|{\mathbb {Q}},p)=2$:

4.5.5 $$\begin{aligned}&\exp \left( -\sum _{r=1}^\infty 2\left( -1\right) ^{\left( p^{fr}\right) ^\times \,/4} \omega \left( \psi \right) ^{\left( p^{fr}\right) ^\times \,/2} p^{fr} H_q\left( \tfrac{1}{8},\tfrac{3}{8},\tfrac{5}{8},\tfrac{7}{8};0,\tfrac{1}{4},\tfrac{1}{2},\tfrac{3}{4}\,|\,t\right) \frac{\left( p^{-s}\right) ^{fr}}{fr} \right) \nonumber \\&\quad = L_{p^f}\left( H\left( \tfrac{1}{8},\tfrac{3}{8},\tfrac{5}{8},\tfrac{7}{8};0,\tfrac{1}{4},\tfrac{1}{2},\tfrac{3}{4}\,|\,t\right) ,p^{1-s},\phi _{\psi }\phi _{-1}\right) ^{2/f}\nonumber \\&\quad = L_{p^f}\left( H\left( \tfrac{1}{8},\tfrac{3}{8},\tfrac{5}{8},\tfrac{7}{8};0,\tfrac{1}{4},\tfrac{1}{2},\tfrac{3}{4}\,|\,t\right) ,p^{1-s},\phi _{\psi }\phi _{-1}\right) ^{r\left( M|{\mathbb {Q}},p\right) } \nonumber \\&\quad = L_p\left( H\left( \tfrac{1}{8},\tfrac{3}{8},\tfrac{5}{8},\tfrac{7}{8};0,\tfrac{1}{4},\tfrac{1}{2},\tfrac{3}{4}\,|\,t\right) , {\mathbb {Q}}\left( \sqrt{-1}\right) ,p^{1-s},\phi _{\psi }\phi _{-1}\right) . \end{aligned}$$

$$\square $$

### The pencil ${\mathsf {L}}_4$

Here, we prove Theorem 1.4.1(e).

#### Proposition 4.6.1

For the pencil ${\mathsf {L}}_4$,

$$\begin{aligned} L_S\left( X_{{\mathsf {L}}_4,\psi }, s\right)&= L_S\left( H\left( \tfrac{1}{4}, \tfrac{1}{2}, \tfrac{3}{4}; 0, 0, 0\,|\, t\right) , s\right) \zeta \left( s-1\right) ^2 \\&\quad \cdot L_S\left( H\left( \tfrac{1}{5}, \tfrac{2}{5}, \tfrac{3}{5}, \tfrac{4}{5}; 0, \tfrac{1}{4}, \tfrac{1}{2}, \tfrac{3}{4} \,|\, t^{-1}\right) , {\mathbb {Q}}\left( \zeta _5\right) , s-1\right) . \end{aligned}$$

#### Proof

By Proposition 3.6.3, we have that

$$\begin{aligned} \#X_{{\mathsf {L}}_4}\left( \psi \right)&=q^2+3q+1+H_q\left( \tfrac{1}{4},\tfrac{1}{2},\tfrac{3}{4};0,0,0\,|\,t\right) \\&\quad + 4q\delta [q\equiv 1 ~({\text {mod}}~{5})]H_q\left( \tfrac{1}{5},\tfrac{2}{5}, \tfrac{3}{5},\tfrac{4}{5} ;0, \tfrac{1}{4},\tfrac{1}{2}, \tfrac{3}{4}\,|\,t^{-1}\right) . \end{aligned}$$

The extra two summands of *q* in the point count correspond to the *L*-series factor $\zeta (s-1)^2$. We now focus on the remaining new summand $4qH_q(\tfrac{1}{5},\tfrac{2}{5}, \tfrac{3}{5},\tfrac{4}{5} ;0, \tfrac{1}{4},\tfrac{1}{2}, \tfrac{3}{4}\,|\,\psi ^{4})$ that occurs exactly when $q \equiv 1 \pmod 5$. Let *f* be the order of *p* in $({\mathbb {Z}}/5{\mathbb {Z}})^\times$, which divides 4. Take $M = {\mathbb {Q}}(\zeta _5)$. We know that $K = {\mathbb {Q}}$ for all possible *p*, hence $r(M|K, p) = 4f^{-1}$. By Lemma 4.1.9, we have

4.6.2 $$\begin{aligned}&\exp \left( -\sum _{r=1}^\infty 4p^{rf}H_{p^{rf}}\left( \tfrac{1}{5},\tfrac{2}{5}, \tfrac{3}{5},\tfrac{4}{5} ;0, \tfrac{1}{4},\tfrac{1}{2}, \tfrac{3}{4}\,|\,t^{-1}\right) \frac{\left( p^{-s}\right) ^{rf}}{fr}\right) \nonumber \\&\quad = L_{p^f}\left( H\left( \tfrac{1}{5},\tfrac{2}{5}, \tfrac{3}{5},\tfrac{4}{5} ;0, \tfrac{1}{4},\tfrac{1}{2}, \tfrac{3}{4}\,|\,t^{-1}\right) , p^{1-s}\right) ^{4/f} \nonumber \\&\quad = L_{p^f}\left( H\left( \tfrac{1}{5},\tfrac{2}{5}, \tfrac{3}{5},\tfrac{4}{5} ;0, \tfrac{1}{4},\tfrac{1}{2}, \tfrac{3}{4}\,|\,t^{-1}\right) , p^{1-s}\right) ^{r\left( M|{\mathbb {Q}},p\right) } \nonumber \\&\quad = L_p\left( H\left( \tfrac{1}{5},\tfrac{2}{5}, \tfrac{3}{5},\tfrac{4}{5} ;0, \tfrac{1}{4},\tfrac{1}{2}, \tfrac{3}{4}\,|\,t^{-1}\right) ,{\mathbb {Q}}\left( \zeta _5\right) , p^{1-s}\right) . \end{aligned}$$

$$\square $$

### Algebraic hypergeometric functions

We now turn to some applications of our main theorem. We begin in this section by setting up a discussion of explicit identification of the algebraic hypergeometric functions that arise in our decomposition, following foundational work of Beukers–Heckman [4].

Recall the hypergeometric function $F(z)=F(\varvec{\alpha };\varvec{\beta } \,|\, z)$ (Definition 2.4.1) for parameters $\varvec{\alpha },\varvec{\beta }$. For certain special parameters, this function may be *algebraic* over ${\mathbb {C}}(z)$, i.e., the field ${\mathbb {C}}(z,F(z))$ is a finite extension of ${\mathbb {C}}(z)$. By a criterion of Beukers–Heckman, *F*(*z*) is algebraic if and only if the parameters interlace (ordering the parameters, they alternate between elements of $\varvec{\alpha }$ and $\varvec{\beta }$) [4, Theorem 4.8]; moreover, all sets of interlacing parameters are classified [4, Theorem 7.1]. We see in Main Theorem 1.4.1 that for all, but the common factor $L_S(H(\tfrac{1}{4},\tfrac{1}{2},\tfrac{3}{4};0,0,0\,|\,t),s)$, the parameters interlace, so this theory applies.

#### Conjecture 4.7.1

Let $t \in {\mathbb {Q}}$, let $\varvec{\alpha },\varvec{\beta }$ with $\#\varvec{\alpha }=\varvec{\beta }=d$ be such that the hypergeometric function $F(\varvec{\alpha };\varvec{\beta } \,|\, z)$ is algebraic. Let *M* satisfy (4.1.6). Then $L_S(H(\varvec{\alpha },\varvec{\beta }\,|\,t),M,s)$ is an Artin *L*-series of degree $d[M:K_{\varvec{\alpha },\varvec{\beta }}]$; in particular, for all good primes *p*, we have $L_p(H(\varvec{\alpha },\varvec{\beta }\,|\,t),M,T) \in 1+T{\mathbb {Q}}[T]$ a polynomial of degree $d[M:K_{\varvec{\alpha },\varvec{\beta }}]$.

Conjecture 4.7.1 is implicit in work of Katz [31, Chapter 8], and there is current, ongoing work on the theory of hypergeometric motives that is expected to prove this conjecture, at least for certain choices of *M*. An explicit version of Conjecture 4.7.1 could be established in each case for the short list of parameters that arise in our Main Theorem. For example, we can use the following proposition about *L*-series and apply it for the family ${\mathsf {F}}_4$, proving a conjecture of Duan [19].

#### Proposition 4.7.2

(Cohen) We have the following *L*-series relations:

4.7.3 $$\begin{aligned} \begin{aligned} L_S\left( H\left( \tfrac{1}{4}, \tfrac{3}{4}; 0, \tfrac{1}{2}\,|\, \psi ^{-4}\right) , s, \phi _{-1}\right)&= L_S\left( s, \phi _{1-\psi ^2}\right) L_S\left( s,\phi _{ -1-\psi ^2}\right) , \\ L_S\left( \tfrac{1}{2};0\,|\,\psi ^{-4}, {\mathbb {Q}}\left( \sqrt{-1}\right) ,s, \phi _{\sqrt{-1}}\right)&= L_S\left( s, \phi _{2\left( 1-\psi ^4\right) }\right) L_S\left( s,\phi _{-2\left( 1-\psi ^4\right) }\right) , \end{aligned} \end{aligned}$$

where $\phi _a = \biggl (\displaystyle {\frac{a}{p}}\biggr )$ is the Legendre symbol.

#### Proof

The hypergeometric *L*-series were computed explicitly by Cohen [13, Propositions 6.4 and 7.32], and the above formulation follows directly from this computation. $\square$

More generally, Naskręcki [44] has given an explicit description for algebraic hypergeometric *L*-series of low degree defined over ${\mathbb {Q}}$ using the variety defined by Beukers–Cohen–Mellit [3].

Proposition 4.7.2, plugged into our hypergeometric decomposition, gives an explicit decomposition of the polynomial $Q_{{\mathsf {F}}_4, \psi ,q}$ as follows.

#### Corollary 4.7.4

We have:

$$\begin{aligned} \begin{aligned}&Q_{{\mathsf {F}}_4, \psi ,q}(T) \\&\quad = {\left\{ \begin{array}{ll} \left( 1-\textstyle {\biggl (\displaystyle {\frac{1-\psi ^2}{q}}\biggr )}qT\right) ^3\left( 1-\biggl (\displaystyle {\frac{-1-\psi ^2}{q}}\biggr )qT\right) ^3 \left( 1-\biggl (\displaystyle {\frac{1-\psi ^4}{q}}\biggr )qT\right) ^{12}, &{}\quad \text { if } q \equiv 1 ~({\text {mod}}~{4}); \\ \left( 1-\biggl (\displaystyle {\frac{1-\psi ^2}{q}}\biggr )qT\right) ^3(1-qT)^6(1+qT)^6, &{}\quad \text { if } q \equiv 3 ~({\text {mod}}~{4}). \end{array}\right. } \end{aligned} \end{aligned}$$

where $\biggl (\displaystyle {\frac{a}{q}}\biggr )$ denotes the Jacobi symbol.

Corollary 4.7.4 explains why the field of definition of the Picard group involves square roots of $1 + \psi ^2$ and $1 - \psi ^2$.

### Applications to zeta functions

To conclude, we give an application to zeta functions. In Sects. 2 and 3, we established a relationship between the periods and the point counts for our collection of invertible K3 polynomial families and hypergeometric functions. In particular, both the periods and the point counts decompose naturally in terms of the group action into hypergeometric components.

It is easy to see that the zeta function is the characteristic polynomial of Frobenius acting on our cohomology (i.e., the collection of periods). In this sense, both Sects. 2 and 3 suggest that, as long as the group action and the action of Frobenius commute, the splitting of Frobenius by the group action translates into factors, each corresponding to the Frobenius acting only on a given isotypical component of the action. However, a priori we only know that this factorization over ${\overline{{\mathbb {Q}}}}$ (see, e.g., work of Miyatani [42]).

Thus, we have the following corollary of Main Theorem 1.4.1.

#### Corollary 4.8.1

Assuming Conjecture 4.7.1, for smooth $X_{\diamond ,\psi ,q}$, the polynomials $Q_{\diamond ,\psi ,q}(T)$ factor over ${\mathbb {Q}}[T]$ under the given hypothesis as follows: 
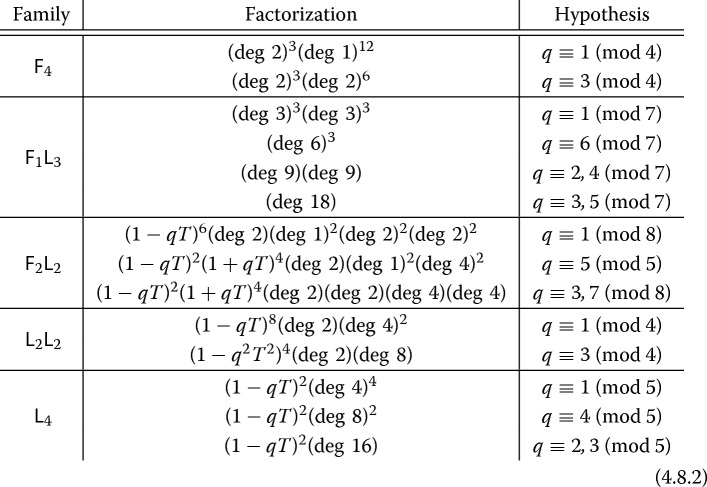


The factorization in Corollary 4.8.1 is to be read as follows: For the family ${\mathsf {L}}_2{\mathsf {L}}_2$ when $q \equiv 1\pmod {4}$, we have $Q_{\diamond ,\psi ,q}(T)=(1-qT)^8 Q_1(T)Q_2(T)^2$ where $\deg Q_1(T)=2$ and $\deg Q_2(T)=4$, but we do not claim that $Q_1,Q_2$ are irreducible. A complete factorization into irreducibles depends on $\psi \in {\mathbb {F}}_q^\times$ and can instead be computed from the explicit Artin *L*-series.

#### Proof

For each case, we need to identify the field of definition for the terms associated to hypergeometric functions other than $H(\tfrac{1}{4}, \tfrac{1}{2}, \tfrac{3}{4}; 0, 0, 0\,|\, t)$ and check the degrees of the resulting zeta function factors using Lemma 4.1.9 and Conjecture 4.7.1.

$${\mathsf {F}}_4$$.
The case where $q\equiv 3 \pmod 4$ is straightforward from the statement of Proposition 4.2.1. In the case where $q \equiv 1 \pmod 4$, we see in the proof that the *L*-series
  $$\begin{aligned} L_S\left( H_q\left( \tfrac{1}{2};0\,|\,t\right) , {\mathbb {Q}}\left( \sqrt{-1}\right) , s-1, \phi _{\sqrt{-1}}\right) \end{aligned}$$
  factors into a square (see Eqs. (4.2.5) and (4.2.6)).
$${\mathsf {F}}_1{\mathsf {L}}_3$$.
In the case where $q\equiv 1,2,4 \pmod 7$, we see in Eq. (4.3.3) that the *L*-series associated to $H(\tfrac{1}{14}, \tfrac{9}{14}, \tfrac{11}{14}; 0, \tfrac{1}{4}, \tfrac{3}{4} \,|\, t)$ factorizes into two terms with multiplicity 3/*f* where *f* is the order of *q* in $({\mathbb {Z}}/7{\mathbb {Z}})^\times$. The analogous argument holds for when $q\equiv 3,5,6 \pmod 7$ using Eq. (4.3.5) to see that the *L*-series factors into one term with multiplicity 6/*f*.
$${\mathsf {F}}_2{\mathsf {L}}_2$$.
The explicit factors follow directly from Eq. (4.4.3). The next two factors come from the *L*-series $L_S( H(\tfrac{1}{4}, \tfrac{3}{4}; 0, \tfrac{1}{2} \,|\, t), s-1, \phi _{-1})$ and $L_S( H( \tfrac{1}{2};0 \,|\, t) , {\mathbb {Q}}(\sqrt{-1}), s-1, \phi _{\sqrt{-1}})$ were dealt with in the ${\mathsf {F}}_4$ case. The zeta function factorization implied by the *L*-series $L_S( H(\tfrac{1}{8}, \tfrac{5}{8}; 0, \tfrac{1}{4} \,|\, t^{-1}), {\mathbb {Q}}(\zeta _8), s-1)$ follows by using Eqs. (4.4.4), (4.4.6), and (4.4.7).
$${\mathsf {L}}_2{\mathsf {L}}_2$$.
The explicit factors follow directly from Eq. (4.5.4). The next factor has been dealt with above. The final factor is implied by Eq. (4.5.5).
$${\mathsf {L}}_4$$.
The term $\zeta (s-1)^2$ gives the $(1-qT)^2$ factor. The last factor is direct from Eq. (4.6.2). $\square$

#### Example 4.8.3

Because the reciprocal roots of $Q_{\diamond ,\psi ,q}(T)$ are of the form *q* times a root of 1, the factors of $Q_{\diamond ,\psi ,q}(T)$ over ${\mathbb {Z}}$ are of the form $\Phi (qT)$, where $\Phi$ is a cyclotomic polynomial. We now give the explicit zeta functions for the case where $q=281$ and $\psi =18$ in the table below. We use a SageMath interface to C code written by Costa, which is described in a paper of Costa–Tschinkel [14]. Note that the factorizations in Corollary 4.8.1 are sharp for the families ${\mathsf {F}}_1{\mathsf {L}}_3$ and ${\mathsf {L}}_4$. 
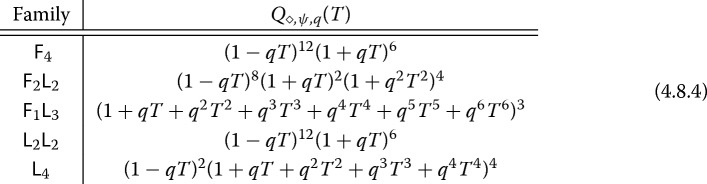


## Appendix A. Remaining Picard–Fuchs equations

In this appendix, we provide the details in the computation of the remaining three pencils ${\mathsf {F}}_2{\mathsf {L}}_2$, ${\mathsf {L}}_2{\mathsf {L}}_2$, and ${\mathsf {L}}_4$. We follow the same strategy as in Sects. 2.5–2.6.

### A.1. The ${\mathsf {F}}_2{\mathsf {L}}_2$ pencil

Take the pencil

$$\begin{aligned} F_{\psi } :=x_0^4+x_1^4 + x_2^3 x_3 + x_3^3x_2 - 4\psi x_0x_1x_2x_3 \end{aligned}$$

that defines the pencil of projective hypersurfaces $X_{\psi } = Z(F_\psi ) \subset {\mathbb {P}}^3$. There is a ${\mathbb {Z}}/8{\mathbb {Z}}$ scaling symmetry of this family generated by the element

$$\begin{aligned} g (x_0: x_1: x_2: x_3) = (\xi ^2 x_0: x_1: \xi x_2 : \xi ^5 x_3) \end{aligned}$$

where $\xi$ is a primitive eighth root of unity. There are eight characters $\chi _k: H \rightarrow {\mathbb {C}}^\times$, where $\chi _k(g) = \xi ^k$. We can again decompose *V* into subspaces $W_{\chi _k}$ and write their monomial bases. Note that the monomial bases for $W_{\chi _1}, W_{\chi _3}, W_{\chi _5},$ and $W_{\chi _7}$ are the same up to transpositions of $x_0$ and $x_1$ or $x_2$ and $x_3$ which leave the polynomial invariant; thus, they have the same Picard–Fuchs equations. The monomial bases for $W_{\chi _2}$ and $W_{\chi _6}$ are related by transposing $x_0$ and $x_1$, so they also have the same Picard–Fuchs equations. So, we are left with four types of monomial bases:

(i) $W_{\chi _0}$ has monomial basis $\{x_0x_1x_2x_3\}$;
(ii) $W_{\chi _1}$ has monomial basis $\{x_2^2x_0x_3, x_0^2x_1x_3\}$;
(iii) $W_{\chi _2}$ has monomial basis $\{x_0^2x_2x_3, x_1^2x_2^2, x_1^2x_3^2\}$; and
(iv) $W_{\chi _4}$ has monomial basis $\{x_0^2x_1^2,x_2^2x_3^2,x_2^2x_0x_1, x_3^2x_0x_1\}$.

Using (2.3.3), we compute the following period relations:

A.1.1 $$\begin{aligned} \begin{aligned} {\mathbf {v}} + (4,0,0,0)&= \frac{1+v_0}{4(\omega + 1)} {\mathbf {v}} + \psi ({\mathbf {v}}+(1,1,1,1)), \\ {\mathbf {v}} + (0,4,0,0)&= \frac{1+v_1}{4(\omega + 1)} {\mathbf {v}} + \psi ({\mathbf {v}}+(1,1,1,1)), \\ {\mathbf {v}} + (0,0,3,1)&= \frac{3(v_2+1)-(v_3+1)}{8(\omega + 1)} {\mathbf {v}} + \psi ({\mathbf {v}}+(1,1,1,1)), \\ {\mathbf {v}} + (0,0,1,3)&= \frac{-(v_2+1) + 3(v_3+1)}{8(\omega + 1)} {\mathbf {v}} + \psi ({\mathbf {v}}+(1,1,1,1)). \end{aligned} \end{aligned}$$

We can now use the diagram method to prove the following proposition.

#### Proposition A.1.2

For the ${\mathsf {F}}_2{\mathsf {L}}_2$ family, the primitive cohomology group $H^2_{\text {prim}}(X_{{\mathsf {F}}_2{\mathsf {L}}_2, \psi }, {\mathbb {C}})$ has 15 periods whose Picard–Fuchs equations are hypergeometric differential equations as follows:

$$\begin{aligned} \begin{aligned} 3 \text { periods are annihilated by } D\left( \tfrac{1}{4}, \tfrac{1}{2}, \tfrac{3}{4} ; 1, 1, 1 \,|\,\psi ^{-4}\right) , \\ 2 \text { periods are annihilated by } D\left( \tfrac{1}{4}, \tfrac{3}{4} ; 1, \tfrac{1}{2} \,|\,\psi ^{-4}\right) , \\ 2 \text { periods are annihilated by } D\left( \tfrac{1}{2} ; 1 \,|\,\psi ^{4}\right) , \\ 4 \text { periods are annihilated by } D\left( \tfrac{1}{8}, \tfrac{5}{8} ; 1, \tfrac{1}{4} \,|\,\psi ^{4}\right) , \text {and } \\ 4 \text { periods are annihilated by } D\left( \tfrac{-3}{8}, \tfrac{1}{8} ; 0, \tfrac{1}{4} \,|\,\psi ^{4}\right) . \end{aligned} \end{aligned}$$

We consider each of these in turn.

#### Lemma A.1.3

The Picard–Fuchs equation associated to the periods $\psi (2,2,0,0)$ and $\psi (0,0,2,2)$ is the hypergeometric differential equation $D(\tfrac{1}{4}, \tfrac{3}{4} ; 1, \tfrac{1}{2} \,|\,\psi ^{-4})$.

#### Proof

For the periods (2, 2, 0, 0) and (0, 0, 2, 2), corresponding to the quartic monomials $x_0^2x_1^2$ and $x_2^2x_3^2$, we use the diagram

and get the relations

A.1.4 $$\begin{aligned} \begin{aligned} \eta (2,2,0,0) = \psi ^2(\eta +1)(0,0,2,2),\\ \eta (0,0,2,2) = \psi ^2(\eta +1)(2,2,0,0). \end{aligned} \end{aligned}$$

For the periods (2, 2, 0, 0) and (0, 0, 2, 2), corresponding to the quartic monomials $x_0^2x_1^2$ and $x_2^2x_3^2$, we get the same Picard–Fuchs equation

A.1.5 $$\begin{aligned} \begin{aligned} \left[ (\eta -2)\eta - \psi ^4(\eta +3)(\eta +1) \right] . \end{aligned} \end{aligned}$$

By multiplying by $\psi$ and substituting $t = \psi ^{-4}$ and $\theta = t \displaystyle {\frac{\mathrm {d}}{\mathrm {d}t}} = -\eta /4$, we get

$$\begin{aligned} \psi \left[ (\eta -2)\eta - \psi ^4(\eta +3)(\eta +1) \right] (2,2,0,0)&= 0, \\ \left[ (\eta -3)(\eta -1) - \psi ^4(\eta +2)\eta \right] \psi (2,2,0,0)&= 0, \\ \left[ \left( \theta +\tfrac{3}{4}\right) \left( \theta +\tfrac{1}{4}\right) - t^{-1} \left( \theta -\tfrac{1}{2}\right) \theta \right] \psi (2,2,0,0)&= 0, \\ \left[ \left( \theta -\tfrac{1}{2}\right) \theta - t\left( \theta +\tfrac{3}{4}\right) \left( \theta +\tfrac{1}{4}\right) \right] \psi (2,2,0,0)&= 0, \end{aligned}$$

which is the hypergeometric differential equation $D(\tfrac{1}{4}, \tfrac{3}{4} ; 1, \tfrac{1}{2} \,|\,\psi ^{-4})$. $\square$

#### Lemma A.1.6

The Picard–Fuchs equation associated to the periods (2, 0, 1, 1) is the hypergeometric differential equation $D(\tfrac{1}{2} ; 1 \,|\,\psi ^{4})$.

#### Proof

For the period (2, 0, 1, 1), corresponding to the quartic monomial $x_0^2x_2x_3$, we use the diagram

One can see that $(2,0,4,2) = \frac{1}{8}(2 + \eta ) (2,0,1,1)$, which one can then use to show that

A.1.7 $$\begin{aligned} \begin{aligned} \eta (2,0,1,1)&= 8 \psi ^2(0,2,3,3) \\&= 8 \psi ^3 (1,3,3,1) \\&= 8 \psi ^4 (2,0,4,2) \\&= \psi ^4 (\eta +2) (2,0,1,1). \end{aligned} \end{aligned}$$

Thus, the period (2, 0, 1, 1) corresponding to the quartic monomial $x_0^2x_2x_3$ satisfies the differential equation:

$$\begin{aligned} \left[ \eta - \psi ^4(\eta +2)\right] (2,0,1,1)=0. \end{aligned}$$

By substituting $u = \psi ^{4}$ and $\sigma = u \displaystyle {\frac{\mathrm {d}}{\mathrm {d}u}} = 4\eta$, we get

$$\begin{aligned} \left[ \eta - \psi ^4(\eta +2)\right] (2,0,1,1)&=0, \\ \left[ \sigma - u(\sigma + \tfrac{1}{2})\right] (2,0,1,1)&=0. \end{aligned}$$

$$\square $$

#### Lemma A.1.8

The Picard–Fuchs equations associated to the periods $(2,1,0,1),\psi ^3(1,0,2,1)$ are the hypergeometric differential equations $D(\tfrac{1}{8}, \tfrac{5}{8} ; 1, \tfrac{1}{4} \,|\,\psi ^{4}),D(\tfrac{1}{8}, \tfrac{-3}{8} ; 0, \tfrac{1}{4} \,|\,\psi ^{4})$, respectively.

#### Proof

For the period (2, 1, 0, 1), corresponding to the quartic monomial $x_0^2x_1x_3$, we use the diagram

Note that:

A.1.9 $$\begin{aligned} \begin{aligned} \eta (2,1,0,1)&= 8\psi ^3(1,0,3,4),\\ (1,0,3,4)&= \tfrac{1}{8}\left( \eta +\tfrac{3}{2}\right) (1,0,2,1), \\ \eta (1,0,2,1)&= \psi \left( \eta +\tfrac{1}{2}\right) (2,1,0,1). \end{aligned} \end{aligned}$$

We can compute the two periods that satisfy each of the following Picard–Fuchs equations for the four sets of pairs:

A.1.10 $$\begin{aligned} \begin{aligned} \left[ (\eta -3) \eta - \psi ^4\left( \eta + \tfrac{5}{2}\right) \left( \eta + \tfrac{1}{2}\right) \right] (2,1,0,1)&= 0,\\ \left[ (\eta -1) \eta - \psi ^4\left( \eta + \tfrac{7}{2}\right) \left( \eta +\tfrac{3}{2}\right) \right] (1,0,2,1)&= 0. \end{aligned} \end{aligned}$$

With the first Picard–Fuchs equation, we can substitute $u = \psi ^4$, $\sigma = u\textstyle {\frac{\mathrm {d}}{\mathrm {d}u}} = 4 \eta$, and yield the equation:

$$\begin{aligned} \left[ (\eta -3) \eta - \psi ^4\left( \eta + \tfrac{5}{2}\right) \left( \eta + \tfrac{1}{2}\right) \right] (2,1,0,1)&= 0, \\ \left[ \left( \sigma -\tfrac{3}{4}\right) \sigma - u\left( \sigma + \tfrac{5}{8}\right) \left( \sigma + \tfrac{1}{8}\right) \right] (2,1,0,1)&= 0. \end{aligned}$$

which is the hypergeometric differential equation $D(\tfrac{1}{8}, \tfrac{5}{8} ; 1, \tfrac{1}{4} \,|\,u)$. For the second Picard–Fuchs equation, we can multiply by $\psi ^3$ and then substitute to find

$$\begin{aligned} \left[ \left( \eta -1\right) \eta - \psi ^4\left( \eta + \tfrac{7}{2}\right) \left( \eta +\tfrac{3}{2}\right) \right] \left( 1,0,2,1\right)&= 0,\\ \psi ^3 \left[ \left( \eta -1\right) \eta - \psi ^4\left( \eta + \tfrac{7}{2}\right) \left( \eta +\tfrac{3}{2}\right) \right] \left( 1,0,2,1\right)&= 0,\\ \left[ \left( \eta -4\right) \left( \eta -3\right) - \psi ^4\left( \eta + \tfrac{1}{2}\right) \left( \eta -\tfrac{3}{2}\right) \right] \psi ^3\left( 1,0,2,1\right)&= 0,\\ \left[ \left( \sigma -1\right) \left( \sigma -\tfrac{3}{4}\right) - u\left( \sigma + \tfrac{1}{8}\right) \left( \sigma -\tfrac{3}{8}\right) \right] \psi ^3\left( 1,0,2,1\right)&= 0, \end{aligned}$$

which is the hypergeometric function $D(\tfrac{1}{8}, \tfrac{-3}{8} ; 0, \tfrac{1}{4} \,|\,\psi ^{4})$. $\square$

#### Proof of Proposition 2.7.1

The periods annihilated by $D(\tfrac{1}{4}, \tfrac{1}{2}, \tfrac{3}{4} ; 1, 1, 1 \,|\,\psi ^{-4})$ are those corresponding to the holomorphic form. The 2 periods are annihilated by $D(\tfrac{1}{4}, \tfrac{3}{4} ; 1, \tfrac{1}{2} \,|\,\psi ^{-4})$ and are provided by Lemma  A.1.3. The period annihilated by $D(\tfrac{1}{2} ; 1 \,|\,\psi ^{4})$ corresponds to a monomial in the basis for $W_{\chi _2}$, which we compute in Lemma A.1.6. Since $W_{\chi _2}$ and $W_{\chi _6}$ are related by a transposition of $x_0$ and $x_1$, there are two periods annihilated by the hypergeometric differential equation computed here. The 4 periods annihilated by $D(\tfrac{1}{8}, \tfrac{5}{8} ; 1, \tfrac{1}{4} \,|\,\psi ^{4})$ and the 4 periods annihilated by $D(\tfrac{-3}{8}, \tfrac{1}{8} ; 0, \tfrac{1}{4} \,|\,\psi ^{4})$ correspond to the monomial bases for $W_{\chi _1}, W_{\chi _3}, W_{\chi _5},$ and $W_{\chi _7}$, which are the same up to transpositions. We compute in Lemma A.1.8 the Picard–Fuchs equations for $W_{\chi _1}$ which then give us that each of those hypergeometric differential equations annihilates 4 periods. $\square$

### A.2. The ${\mathsf {L}}_2{\mathsf {L}}_2$ pencil

Now, consider

$$\begin{aligned} F_{\psi } :=x_0^3x_1+x_1^3x_0 + x_2^3 x_3 + x_3^3x_2 - 4\psi x_0x_1x_2x_3 \end{aligned}$$

that defines the pencil of projective hypersurfaces $X_{\psi } = Z(F_\psi ) \subset {\mathbb {P}}^3$. There is a ${\mathbb {Z}}/4{\mathbb {Z}}$ symmetry with generator

$$\begin{aligned} g(x_0:x_1:x_2:x_3)&= (\xi x_0:\xi ^5 x_1:\xi ^3 x_2:\xi ^7 x_3), \end{aligned}$$

where $\xi$ is a primitive eighth root of unity. There are four characters $\chi _{a}: H \rightarrow {\mathbb {G}}_m$, where $\chi _{a}(g_1) = \sqrt{-1}^{a}$. We can again decompose *V* into subspaces $W_{\chi _{a}}$. Out of the eight, the subspaces $W_{\chi _{0}}, W_{\chi _{1}}, W_{\chi _{2}},$ and $W_{\chi _{3}}$ are empty. The monomial bases for $W_{\chi _{1}}$ and $W_{\chi _{3}}$ are related by a transposition of the variables $x_0$ and $x_1$, so their Picard–Fuchs equations are the same. We have three types of monomial bases:

(i) $W_{\chi _{0}}$ has monomial basis $\{x_0x_1x_2x_3, x_0^2x_2^2, x_0^2x_3^2, x_1^2x_2^2, x_1^2x_3^2\}$;
(ii) $W_{\chi _{1}}$ has monomial basis $\{x_0^2x_1x_2, x_1^2x_0x_3, x_2^2x_1x_3, x_3^2x_0x_2\}$; and
(iii) $W_{\chi _{2}}$ has monomial basis $\{x_0^2x_1^2, x_2^2x_3^2, x_0^2x_2x_3, x_1^2x_2x_3, x_2^2x_0x_1, x_3^2x_0x_1 \}$.

Using (2.3.3), we compute the following period relations:

A.2.1 $$\begin{aligned} \begin{aligned} {\mathbf {v}}+(3,1,0,0)&= \frac{3(v_0+1) -(v_1+1)}{8(\omega +1)}{\mathbf {v}} + \psi ({\mathbf {v}}+(1,1,1,1)) \\ {\mathbf {v}}+(1,3,0,0)&= \frac{-(v_0+1) +3(v_1+1)}{8(\omega +1)}{\mathbf {v}} + \psi ({\mathbf {v}}+(1,1,1,1)) \end{aligned} \end{aligned}$$

and the two symmetric relations replacing 0, 1 with 2, 3.

#### Proposition A.2.2

For the ${\mathsf {L}}_2{\mathsf {L}}_2$ family, the primitive cohomology group $H^2_{\text {prim}}(X_{{\mathsf {L}}_2{\mathsf {L}}_2, \psi }, {\mathbb {C}})$ has 13 periods whose Picard–Fuchs equations are hypergeometric differential equations as follows:

$$\begin{aligned} \begin{aligned} 3 \text { periods are annihilated by } D\left( \tfrac{1}{4}, \tfrac{1}{2}, \tfrac{3}{4} ; 1, 1, 1 \,|\,\psi ^{-4}\right) , \\ 8 \text { periods are annihilated by } D\left( \tfrac{1}{8}, \tfrac{3}{8}, \tfrac{5}{8}, \tfrac{7}{8} ; 0, \tfrac{1}{4}, \tfrac{1}{2}, \tfrac{3}{4} \,|\,\psi ^{4}\right) , \text {and } \\ 2 \text { periods are annihilated by } D\left( \tfrac{1}{4}, \tfrac{3}{4} ; 1, \tfrac{1}{2} \,|\,\psi ^{4}\right) . \end{aligned} \end{aligned}$$

To prove Proposition A.2.2, we use the diagram method above in a few cases and then use symmetry. We first do two calculations.

#### Lemma A.2.3

The Picard–Fuchs equation associated to the periods (2, 1, 1, 0), (1, 0, 1, 2), (1, 2, 0, 1),  and (0, 1, 2, 1) is the hypergeometric differential equation $D(\tfrac{1}{8}, \tfrac{3}{8}, \tfrac{5}{8}, \tfrac{7}{8} ; 0, \tfrac{1}{4}, \tfrac{1}{2}, \tfrac{3}{4} \,|\,\psi ^{4})$.

#### Proof

To find the Picard–Fuchs equations corresponding to all these cohomology pieces, we use the diagram

We obtain the following relations:

A.2.4 $$\begin{aligned} \begin{aligned} \eta (1,0,1,2)&= \psi \left( \eta +\tfrac{1}{2}\right) (2,1,1,0), \\ \eta (1,2,0,1)&= \psi \left( \eta +\tfrac{1}{2}\right) (1,0,1,2), \\ \eta (0,1,2,1)&= \psi \left( \eta +\tfrac{1}{2}\right) (1,2,0,1), \\ \eta (2,1,1,0)&= \psi \left( \eta +\tfrac{1}{2}\right) (0,1,2,1). \end{aligned} \end{aligned}$$

Using these relations, we can get the Picard–Fuchs equation:

A.2.5 $$\begin{aligned} \left[ (\eta -3) (\eta -2)(\eta -1) \eta - \psi ^4\left( \eta + \tfrac{7}{2}\right) \left( \eta + \tfrac{5}{2}\right) \left( \eta + \tfrac{3}{2} \right) \left( \eta + \tfrac{1}{2}\right) \right] (1,0,1,2) = 0. \end{aligned}$$

By substituting $u = \psi ^4$ and $\sigma = u \displaystyle {\frac{\mathrm {d}}{\mathrm {d}u}} = \tfrac{1}{4} \eta$, we obtain:

$$\begin{aligned}&\left[ (4\sigma -3) (4\sigma -2)(4\sigma -1) 4\sigma - u\left( 4\sigma + \tfrac{7}{2}\right) \left( \eta + \tfrac{5}{2}\right) \left( 4\sigma + \tfrac{3}{2} \right) \left( 4\sigma + \tfrac{1}{2}\right) \right] \\&\quad \cdot (1,0,1,2) = 0, \\&\left[ (\sigma -\tfrac{3}{4}) (\sigma -\tfrac{1}{2})(\sigma -\tfrac{1}{4}) \sigma - u\left( \sigma + \tfrac{7}{8}\right) \left( \sigma + \tfrac{5}{8}\right) \left( \sigma + \tfrac{3}{8} \right) \left( \sigma + \tfrac{1}{8}\right) \right] \\&\quad \cdot (1,0,1,2) = 0, \end{aligned}$$

which is the hypergeometric differential equation $D(\tfrac{1}{8}, \tfrac{3}{8}, \tfrac{5}{8}, \tfrac{7}{8} ; 0, \tfrac{1}{4}, \tfrac{1}{2}, \tfrac{3}{4} \,|\,\psi ^{4})$. The other three Picard–Fuchs equations are the same due to the symmetry in (A.2.4). $\square$

#### Lemma A.2.6

The Picard–Fuchs equation associated to the periods (2, 2, 0, 0) and (0, 0, 2, 2) is the hypergeometric differential equation $D(\tfrac{1}{4}, \tfrac{3}{4} ; 1, \tfrac{1}{2} \,|\,\psi ^{4})$.

#### Proof

We use the diagram

We obtain the following relations:

A.2.7 $$\begin{aligned} \begin{aligned} \eta (0,0,2,2)&= \psi ^2(\eta + 1) (2,2,0,0),\\ \eta (2,2,0,0)&= \psi ^2(\eta + 1) (0,0,2,2), \end{aligned} \end{aligned}$$

giving the following Picard–Fuchs equations:

A.2.8 $$\begin{aligned} \begin{aligned} \left[ (\eta -2)\eta - \psi ^4 \left( \eta + 3\right) \left( \eta + 1\right) \right] (2,2,0,0)&= 0,\\ \left[ (\eta -2)\eta - \psi ^4 \left( \eta + 3\right) \left( \eta + 1\right) \right] (0,0,2,2)&= 0; \end{aligned} \end{aligned}$$

By substituting $u = \psi ^4$ and $\sigma = u\tfrac{\mathrm {d}}{\mathrm {d}u} = \tfrac{1}{4} \eta$, we obtain the hypergeometric form:

$$\begin{aligned} \left[ \left( \sigma -\tfrac{1}{2}\right) \sigma -u \left( \sigma + \tfrac{3}{4}\right) \left( \sigma + \tfrac{1}{4}\right) \right] \left( 2,2,0,0\right) = 0,\\ \left[ \left( \sigma -\tfrac{1}{2}\right) \sigma -u \left( \sigma + \tfrac{3}{4}\right) \left( \sigma + \tfrac{1}{4}\right) \right] \left( 0,0,2,2\right) = 0, \end{aligned}$$

which is the hypergeometric differential equation $D(\tfrac{1}{4}, \tfrac{3}{4} ; 1, \tfrac{1}{2} \,|\,\psi ^{4})$. $\square$

#### Proof of Proposition A.2.2

The first three periods are the same for each family. Next, by Lemma A.2.3, all the monomial basis elements in $W_{\chi _{1}}$ are annihilated by the hypergeometric differential equation $D(\tfrac{1}{8}, \tfrac{3}{8}, \tfrac{5}{8}, \tfrac{7}{8} ; 0, \tfrac{1}{4}, \tfrac{1}{2}, \tfrac{3}{4} \,|\,\psi ^{4})$. Since $W_{\chi _{1}}$ and $W_{\chi _{3}}$ are related by a transposition, we get 8 periods annihilated by it. The Picard–Fuchs equations for the last two periods are given by Lemma A.2.6. $\square$

### A.3. The ${\mathsf {L}}_4$ pencil

Finally, we consider

$$\begin{aligned} F_{\psi } :=x_0^3x_1+x_1^3x_2 + x_2^3 x_3 + x_3^3x_0 - 4\psi x_0x_1x_2x_3, \end{aligned}$$

that defines the pencil of projective hypersurfaces $X_{\psi } = Z(F_\psi ) \subset {\mathbb {P}}^3$. There is a $H={\mathbb {Z}}/5{\mathbb {Z}}$ scaling symmetry on $X_{\psi }$ generated by the element

$$\begin{aligned} g(x_0:x_1:x_2:x_3) = (\xi x_0:\xi ^2x_2:\xi ^4x_2: \xi ^3 x_3) \end{aligned}$$

where $\xi$ is a fifth root of unity. There are five characters $\chi _k: H\rightarrow {\mathbb {C}}^\times$ given by $\chi _k(g) = \xi ^k$. We decompose *V* into five subspaces $W_{\chi _k}$. The monomial bases for $W_{\chi _1}, W_{\chi _2}, W_{\chi _3},$ and $W_{\chi _4}$ are related by a rotation of the variables $x_1, x_2, x_3,$ and $x_4$, so their corresponding Picard–Fuchs equations are the same. We are then left with two types of monomial bases:

(i) $W_{\chi _0}$ has monomial basis $\{x_0x_1x_2x_3, x_0^2x_2^2, x_1^2x_3^2\}$; and
(ii) $W_{\chi _1}$ has monomial basis $\{x_0^2x_1^2, x_1^2x_2x_3, x_3^2x_0x_2, x_2^2x_0x_1\}$.

For this family, we can compute the period relations:

A.3.1 $$\begin{aligned} \begin{aligned} {\mathbf {v}} + (3,1,0,0) = \frac{27(1+v_0)- (1+v_1) +3(1+v_2) - 9(1+v_3)}{80(\omega +1)} {\mathbf {v}} + \psi ({\mathbf {v}}+(1,1,1,1)) \end{aligned} \end{aligned}$$

and its 4 cyclic permutations.

#### Proposition A.3.2

For the family ${\mathsf {L}}_4$, the primitive cohomology group $H^2_{\text {prim}}(X_{{\mathsf {L}}_4, \psi },{\mathbb {C}})$ has 19 periods whose Picard–Fuchs equations are hypergeometric differential equations as follows:

$$\begin{aligned} \begin{aligned} 3 \text { periods are annihilated by } D\left( \tfrac{1}{4}, \tfrac{1}{2}, \tfrac{3}{4} ; 1, 1, 1 \,|\,\psi ^{-4}\right) , \\ 4 \text { periods are annihilated by } D\left( \tfrac{1}{5}, \tfrac{2}{5}, \tfrac{3}{5}, \tfrac{4}{5}; 1, \tfrac{1}{4}, \tfrac{1}{2}, \tfrac{3}{4} \,|\, \psi ^4\right) , \\ 4 \text { periods are annihilated by } D\left( \tfrac{-1}{5}, \tfrac{1}{5}, \tfrac{2}{5}, \tfrac{3}{5}; 0, \tfrac{1}{4}, \tfrac{1}{2}, \tfrac{3}{4} \,|\,\psi ^4\right) , \\ 4 \text { periods are annihilated by } D\left( \tfrac{-2}{5}, \tfrac{-1}{5}, \tfrac{1}{5}, \tfrac{2}{5}; \tfrac{-1}{4}, 0, \tfrac{1}{4}, \tfrac{1}{2} \,|\,\psi ^4\right) , \text {and} \\ 4 \text { periods are annihilated by } D\left( \tfrac{-3}{5}, \tfrac{-2}{5}, \tfrac{-1}{5}, \tfrac{1}{5}; \tfrac{-1}{2}, \tfrac{-1}{4}, 0, \tfrac{1}{4} \,|\,\psi ^4\right) . \end{aligned} \end{aligned}$$

#### Proof

The period associated to the holomorphic form is found by the same strategy as before.

Lastly, we use (A.3.1) to construct the diagram:

and consequently obtain the following relations:

A.3.3 $$\begin{aligned} \begin{aligned} \eta (1,1,2,0)&= \psi \left( \eta + \tfrac{2}{5}\right) (2,2,0,0), \\ \eta (1,0,1,2)&= \psi \left( \eta + \tfrac{1}{5}\right) (1,1,2,0), \\ \eta (0,2,1,1)&= \psi \left( \eta + \tfrac{4}{5}\right) (1,0,1,2), \\ \eta (2,2,0,0)&= \psi \left( \eta + \tfrac{3}{5}\right) (0,2,1,1). \end{aligned} \end{aligned}$$

We then cyclically use these relations to find a recursion which yields the following Picard–Fuchs equations:

A.3.4 $$\begin{aligned} \begin{aligned} \left[ \left( \eta + \tfrac{16}{5}\right) \left( \eta + \tfrac{12}{5}\right) \left( \eta + \tfrac{8}{5}\right) \left( \eta + \tfrac{4}{5}\right) - \tfrac{1}{\psi ^4} (\eta -3)(\eta -2)(\eta -1)\eta \right] (1,0,1,2)&=0, \\ \left[ \left( \eta + \tfrac{17}{5}\right) \left( \eta + \tfrac{13}{5}\right) \left( \eta + \tfrac{9}{5}\right) \left( \eta + \tfrac{1}{5}\right) - \tfrac{1}{\psi ^4} (\eta -3)(\eta -2)(\eta -1)\eta \right] (1,1,2,0)&= 0, \\ \left[ \left( \eta + \tfrac{18}{5}\right) \left( \eta + \tfrac{14}{5}\right) \left( \eta + \tfrac{6}{5}\right) \left( \eta + \tfrac{2}{5}\right) - \tfrac{1}{\psi ^4} (\eta -3)(\eta -2)(\eta -1)\eta \right] (2,2,0,0)&=0, \\ \left[ \left( \eta + \tfrac{19}{5}\right) \left( \eta + \tfrac{11}{5}\right) \left( \eta + \tfrac{7}{5}\right) \left( \eta + \tfrac{3}{5}\right) - \tfrac{1}{\psi ^4} (\eta -3)(\eta -2)(\eta -1)\eta \right] (0,2,1,1)&=0. \end{aligned} \end{aligned}$$

By multiplying these lines by 1, $\psi$, $\psi ^2$, and $\psi ^3$, respectively, substituting $u = \psi ^{4}$, $\sigma = u \displaystyle {\frac{\mathrm {d}}{\mathrm {d}u}}$, and then multiplying by $-u$, we obtain the following equations:

$$\begin{aligned}&\left[ \left( \sigma -\tfrac{3}{4}\right) \left( \sigma -\tfrac{1}{2}\right) \left( \sigma -\tfrac{1}{4}\right) \sigma - u\left( \sigma + \tfrac{4}{5}\right) \left( \sigma + \tfrac{3}{5}\right) \left( \sigma + \tfrac{2}{5}\right) \left( \sigma + \tfrac{1}{5}\right) \right] \\&\quad \cdot \left( 1,0,1,2\right) =0 \\&\left[ \left( \sigma -1\right) \left( \sigma -\tfrac{3}{4}\right) \left( \sigma -\tfrac{1}{2}\right) \left( \sigma -\tfrac{1}{4}\right) - u\left( \sigma + \tfrac{3}{5}\right) \left( \sigma + \tfrac{2}{5}\right) \left( \sigma + \tfrac{1}{5}\right) \left( \sigma - \tfrac{1}{5}\right) \right] \psi \\&\quad \cdot \left( 1,1,2,0\right) = 0 \\&\left[ \left( \sigma -\tfrac{5}{4}\right) \left( \sigma -1\right) \left( \sigma -\tfrac{3}{4}\right) \left( \sigma -\tfrac{1}{2}\right) - u\left( \sigma + \tfrac{2}{5}\right) \left( \sigma + \tfrac{1}{5}\right) \left( \sigma - \tfrac{1}{5}\right) \left( \sigma - \tfrac{2}{5}\right) \right] \psi ^2\\&\quad \cdot \left( 2,2,0,0\right) =0\\&\left[ \left( \sigma -\tfrac{3}{2}\right) \left( \sigma -\tfrac{5}{4}\right) \left( \sigma -1\right) \left( \sigma -\tfrac{3}{4}\right) - u\left( \sigma + \tfrac{1}{5}\right) \left( \sigma - \tfrac{1}{5}\right) \left( \sigma - \tfrac{2}{5}\right) \left( \sigma - \tfrac{3}{5}\right) \right] \psi ^3\\&\quad \cdot \left( 0,2,1,1\right) =0. \end{aligned}$$

These are the claimed hypergeometric differential equations. $\square$

## Appendix B. Finite-field hypergeometric sums

In this Appendix, we write down the details of manipulations of hypergeometric sums.

### B.1. Hybrid definition

In this section, we apply the argument of Beukers–Cohen–Mellit to show that the hybrid definition of the finite-field hypergeometric sum reduces to the classical one. We retain the notation from Sects. 3.1–3.2.

#### Lemma B.1.1

Suppose that *q* is good and splittable for $\varvec{\alpha },\varvec{\beta }$. If $\alpha _i q^{\times }, \beta _i q^{\times }\in {\mathbb {Z}}$ for all $i=1,\dots ,d$, then Definitions 3.1.6 and 3.2.7 agree.

#### Proof

Our proof follows Beukers–Cohen–Mellit [3, Theorem 1.3]. We consider

$$\begin{aligned} G(m+\varvec{\alpha }'q^{\times },-m-\varvec{\beta }'q^{\times }) = \prod _{\alpha _i' \in \varvec{\alpha }'}\frac{g(m+ \alpha _i'q^{\times })}{g(\alpha _i q^{\times })} \prod _{\beta _i' \in \varvec{\beta }'}\frac{g(-m-\beta _i'q^{\times })}{g(-\beta _i q^{\times })}. \end{aligned}$$

We massage this expression, and for simplicity drop the subscripts 0. First,

$$\begin{aligned}&D\left( x\right) \prod _{\alpha _j \in \hat{\varvec{\alpha }}} \left( x- e^{2\pi \sqrt{-1} \alpha _j}\right) = \prod _{j=1}^r \left( x^{p_j} - 1\right) \quad \text {and} \\&D\left( x\right) \prod _{\beta _j \in \tilde{\varvec{\beta }}}\left( x- e^{2\pi \sqrt{-1} \beta _j}\right) = \prod _{j=1}^s \left( x^{q_j} - 1\right) . \end{aligned}$$

Write $$D(x) = \prod _{j=1}^\delta (x- e^{2\pi \sqrt{-1} c_j/q^{\times }})$$. Then

B.1.2 $$\begin{aligned} \begin{aligned}&G(m+\varvec{\alpha }'q^{\times },-m-\varvec{\beta }'q^{\times }) \\&\quad = \left( \prod _{i=1}^r \prod _{j=0}^{p_i-1} \frac{g(m+ jq^{\times }\,/p_i)}{g(jq^{\times }\,/p_i)}\right) \left( \prod _{i=1}^s \prod _{j=0}^{q_i-1} \frac{g(-m-jq^{\times }\,/q_i)}{g(-jq^{\times }\,/q_i)}\right) \prod _{j=1}^\delta \frac{g(c_j)g(-c_j)}{g(m+c_j) g(-m-c_j)}. \end{aligned} \end{aligned}$$

Since $p_i$ divides $q^{\times }$, by the Hasse–Davenport relation (Lemma 3.1.3(c)) we have that

B.1.3 $$\begin{aligned} \prod _{j=0}^{p_i-1} \frac{g(m+ jq^{\times }\,/p_i)}{g(jq^{\times }\,/p_i)} = \frac{-g(p_i m)}{\omega (p_i)^{p_im}}. \end{aligned}$$

Analogously, since $q_i$ divides $q^{\times }$, we use Hasse–Davenport to find that

B.1.4 $$\begin{aligned} \prod _{j=0}^{q_i-1} \frac{g(-m-jq^{\times }\,/q_i)}{g(-jq^{\times }\,/q_i)} = -g(-q_i m) \omega (q_i)^{q_i m}. \end{aligned}$$

Note that if $c_j \ne 0$, then $g(c_j)g(-c_j) = (-1)^{c_j}q$ and 1 otherwise, hence

$$\begin{aligned} \prod _{i=1}^\delta g(c_j)g(-c_j) = (-1)^{\sum c_j} q^{\delta - s(0)}, \end{aligned}$$

where *s*(0) is the multiplicity of 1 in *D*(*x*), or, equivalently, the number of times $c_j$ is 0. Now, note the number of times that $m+c_j = 0$ is the multiplicity of the root $e^{-2\pi \sqrt{-1} m / q^{\times }}$ in *D*(*x*), which, equivalently, is the multiplicity of $e^{2\pi \sqrt{-1} m / q^{\times }}$ in *D*(*x*) as *D*(*x*) is a product of cyclotomic polynomials. This implies that

$$\begin{aligned} \prod _{j=1}^\delta g(m+c_j)g(-m-c_j) = (-1)^{m+c_j} q^{\delta -\lambda (m)}. \end{aligned}$$

We then have that

B.1.5 $$\begin{aligned} \prod _{j=1}^\delta \frac{g(c_j)g(-c_j)}{g(m+c_j) g(-m-c_j)} = \frac{ (-1)^{\sum _j c_j} q^{\delta - s(0)}}{ (-1)^{\sum _j(m+c_j)} q^{\delta -s(m)}} = (-1)^{-\delta m} q^{s(m) - s(0)}. \end{aligned}$$

Combining Eqs. (B.1.3), (B.1.4), and (B.1.5), we then have

$$\begin{aligned}&G(m+\varvec{\alpha }'q^{\times },-m-\varvec{\beta }'q^{\times }) \\&\quad = \left( \prod _{i=1}^r \frac{-g(p_i m)}{\omega (p_i)^{p_im}}\right) \left( \prod _{i=1}^s -g(-q_i m) \omega (q_i)^{q_i m}\right) \left( (-1)^{-\delta m} q^{s(m) - s(0)}\right) \\&\quad = (-1)^{r+s}q^{s(m) - s(0)} g(p_1m)\cdots g(p_rm) g(-q_1m) \cdots g(-q_sm) \\&\qquad \cdot \omega ((-1)^{\delta }p_1^{-p_1} \cdots p_r^{-p_r} q_1^{q_1} \cdots q_s^{q_s})^m\\&\quad = (-1)^{r+s}q^{s(m) - s(0)} g(p_1m)\cdots g(p_rm) g(-q_1m) \cdots g(-q_sm) \omega ((-1)^{\delta }M)^m. \end{aligned}$$

By plugging this equation into Definition 3.1.6 for the appropriate factors, we obtain the quantity given in Definition 3.2.7. $\square$

### B.2. The pencil ${\mathsf {F}}_2{\mathsf {L}}_2$

#### Proposition B.2.1

The number of ${\mathbb {F}}_q$-points on ${\mathsf {F}}_2{\mathsf {L}}_2$ can be written in terms of hypergeometric functions, as follows:

- If $$q \equiv 3\pmod 4$$, then
  $$\begin{aligned} X_{{\mathsf {F}}_2{\mathsf {L}}_2,\psi }\left( {\mathbb {F}}_q\right)&=q^2-q+1+H_q\left( \tfrac{1}{4},\tfrac{1}{2},\tfrac{3}{4};0,0,0\,|\,\psi ^{-4}\right) - q H_q\left( \tfrac{1}{4},\tfrac{3}{4};0,\tfrac{1}{2} \,|\,\psi ^{-4}\right) . \end{aligned}$$
- If $$q\equiv 5\pmod 8$$, then
  $$\begin{aligned} X_{{\mathsf {F}}_2{\mathsf {L}}_2,\psi }\left( {\mathbb {F}}_q\right)&=q^2-q+1+H_q\left( \tfrac{1}{4},\tfrac{1}{2},\tfrac{3}{4};0,0,0\,|\,\psi ^{-4}\right) \\&\quad +qH_q\left( \tfrac{1}{4},\tfrac{3}{4};0,\tfrac{1}{2} \,|\,\psi ^{-4}\right) -2qH_q\left( \tfrac{1}{2};0\,|\,\psi ^{-4}\right) . \end{aligned}$$
- If $$q\equiv 1\pmod 8$$, then
  $$\begin{aligned} X_{{\mathsf {F}}_2{\mathsf {L}}_2,\psi }\left( {\mathbb {F}}_q\right)&= q^2+7q +1 +H_q\left( \tfrac{1}{4},\tfrac{1}{2},\tfrac{3}{4};0,0,0\,|\,\psi ^{-4}\right) +qH_q\left( \tfrac{1}{4}, \tfrac{3}{4}; 0, \tfrac{1}{2} \,|\, \psi ^{-4}\right) \\&\quad + 2q H_q\left( \tfrac{1}{2}; 0 \,|\, \psi ^{-4}\right) + 2\omega \left( 2\right) ^{q^{\times }\,/4}qH_q\left( \tfrac{1}{8}, \tfrac{5}{8}; 0, \tfrac{1}{4} \,|\, \psi ^4\right) \\&\quad + 2\omega \left( 2\right) ^{q^{\times }\,/4}qH_q\left( \tfrac{3}{8}, \tfrac{7}{8}; 0, \tfrac{3}{4} \,|\, \psi ^4\right) . \end{aligned}$$

#### Remark B.2.2

Notice that the hypergeometric functions appearing in the point count correspond to the Picard–Fuchs equations in Proposition 2.7.1. We also see the appearance of six additional trivial factors.

#### Step 1: Computing and clustering the characters

To use Theorem 3.3.3 we compute the subset $S\subset ({\mathbb {Z}}/q^{\times }{\mathbb {Z}})^r$ given by the constraints in (3.3.1).

- If $q\equiv 3 \pmod 4$, then $S$ can be clustered in the following way:
  (i) the set $S_1 = \{ k(1,1,1,1,-4) : k \in {\mathbb {Z}}/q^{\times }{\mathbb {Z}}\}$ and
  (ii) the set $S_4 = \{ k(1,1,1,1,-4) + \tfrac{q^{\times }}{2}(0,0,1,1,0): k \in {\mathbb {Z}}/q^{\times }{\mathbb {Z}}\}$.
- If $q\equiv 5 \pmod 8$, then $S$ contains the two sets above and:
  (i) the set $S_5 = \{ k(1,1,1,1,-4) + \tfrac{q^{\times }}{4}(0,2,1,1,0): k \in {\mathbb {Z}}/q^{\times }{\mathbb {Z}}\}$ and
  (ii) the set $S_6 = \{ k(1,1,1,1,-4) + 3\tfrac{q^{\times }}{4}(0,2,1,1,0): k \in {\mathbb {Z}}/q^{\times }{\mathbb {Z}}\}$.
- If $q\equiv 1 \pmod 8$, then $S$ contains the four sets above and
  (i) two sets of the form $S_{10} =\{ k(1,1,1,1,-4) + \tfrac{q^{\times }}{8}(0,2,1,5,0): k \in {\mathbb {Z}}/q^{\times }{\mathbb {Z}}\}$ and
  (ii) two sets of the form $S_{11} =\{ k(1,1,1,1,-4) + \tfrac{q^{\times }}{8}(0,6,7,3,0): k \in {\mathbb {Z}}/q^{\times }{\mathbb {Z}}\}$.

#### Step 2: Counting points on the open subset with nonzero coordinates

##### Lemma B.2.3

Suppose $$\psi \in {\mathbb {F}}_q^{\times }$$.

- If $$q \equiv 3 \pmod 4$$, then
  B.2.4 $$\begin{aligned} \#U_{{\mathsf {F}}_2{\mathsf {L}}_2, \psi }\left( {\mathbb {F}}_q\right) = q^2-3q+1+H_q\left( \tfrac{1}{4},\tfrac{1}{2},\tfrac{3}{4};0,0,0\,|\,\psi ^{-4}\right) - qH_q\left( \tfrac{1}{4}, \tfrac{3}{4}; 0, \tfrac{1}{2} \,|\, \psi ^{-4}\right) . \end{aligned}$$
- If $$q \equiv 5 \pmod 8$$, then
  B.2.5 $$\begin{aligned} \begin{aligned} \#U_{{\mathsf {F}}_2{\mathsf {L}}_2, \psi }\left( {\mathbb {F}}_q\right)&= q^2-3q+3+H_q\left( \tfrac{1}{4},\tfrac{1}{2},\tfrac{3}{4};0,0,0\,|\,\psi ^{-4}\right) + qH_q\left( \tfrac{1}{4}, \tfrac{3}{4}; 0, \tfrac{1}{2} \,|\, \psi ^{-4}\right) \\&\quad -2q H_q\left( \tfrac{1}{2}; 0 \,|\, \psi ^{-4}\right) - 2 \left( \frac{g\left( \tfrac{q^{\times }}{4}\right) ^2 + g\left( \tfrac{3q^{\times }}{4}\right) ^2}{g\left( \tfrac{q^{\times }}{2}\right) }\right) . \end{aligned} \end{aligned}$$
- If $$q \equiv 1 \pmod 8$$, then
  B.2.6 $$\begin{aligned} \begin{aligned} \#U_{{\mathsf {F}}_2{\mathsf {L}}_2, \psi }\left( {\mathbb {F}}_q\right)&= q^2-3q+7+H_q\left( \tfrac{1}{4},\tfrac{1}{2},\tfrac{3}{4};0,0,0\,|\,\psi ^{-4}\right) +qH_q\left( \tfrac{1}{4}, \tfrac{3}{4}; 0, \tfrac{1}{2} \,|\, \psi ^{-4}\right) \\&\quad + 2q H_q\left( \tfrac{1}{2}; 0 \,|\, \psi ^{-4}\right) + 2\omega \left( 2\right) ^{q^{\times }\,/4}qH_q\left( \tfrac{1}{8}, \tfrac{5}{8}; 0, \tfrac{1}{4} \,|\, \psi ^4\right) \\&\quad + 2\omega \left( 2\right) ^{q^{\times }\,/4}qH_q\left( \tfrac{3}{8}, \tfrac{7}{8}; 0, \tfrac{3}{4} \,|\, \psi ^4\right) \\&\quad - 2\left( \frac{g\left( \tfrac{q^{\times }}{4}\right) ^2 + g\left( \tfrac{3q^{\times }}{4}\right) ^2}{g\left( \tfrac{q^{\times }}{2}\right) }\right) - \tfrac{4}{q}g\left( \tfrac{q^{\times }}{8}\right) g\left( \tfrac{5q^{\times }}{8}\right) g\left( \tfrac{q^{\times }}{4}\right) \\&\quad - \tfrac{4}{q}g\left( \tfrac{3q^{\times }}{8}\right) g\left( \tfrac{7q^{\times }}{8}\right) g\left( \tfrac{3q^{\times }}{4}\right) . \end{aligned} \end{aligned}$$

##### Proof

If $q \equiv 3\pmod 4$, then by Lemmas 3.4.4 and 3.4.7

B.2.7 $$\begin{aligned} \begin{aligned} \#U_{{\mathsf {F}}_2{\mathsf {L}}_2, \psi }\left( {\mathbb {F}}_q\right)&= \sum _{s \in S_1} \omega \left( a\right) ^{-s} c_s + \sum _{s \in S_4} \omega \left( a\right) ^{-s} c_s \\&= \left( q^2-3q+3+H_q\left( \tfrac{1}{4},\tfrac{1}{2},\tfrac{3}{4};0,0,0\,|\,\psi ^{-4}\right) \right) + \\&\qquad -2 - qH_q\left( \tfrac{1}{4}, \tfrac{3}{4}; 0, \tfrac{1}{2} \,|\, \psi ^{-4}\right) \\&= q^2-3q+1+H_q\left( \tfrac{1}{4},\tfrac{1}{2},\tfrac{3}{4};0,0,0\,|\,\psi ^{-4}\right) - qH_q\left( \tfrac{1}{4}, \tfrac{3}{4}; 0, \tfrac{1}{2} \,|\, \psi ^{-4}\right) . \end{aligned} \end{aligned}$$

If $q \equiv 5 \pmod 8$, then $q\equiv 1 \pmod 4$ but $q\not \equiv 1 \pmod 8$, so by Lemmas 3.4.4, 3.4.7, 3.4.12, and 3.4.21

B.2.8 $$\begin{aligned} \begin{aligned} \#U_{{\mathsf {F}}_2{\mathsf {L}}_2, \psi }\left( {\mathbb {F}}_q\right)&= \sum _{s \in S_1} \omega \left( a\right) ^{-s} c_s + \sum _{s \in S_4} \omega \left( a\right) ^{-s} c_s + \sum _{s \in S_5} \omega \left( a\right) ^{-s} c_s + \sum _{s \in S_6} \omega \left( a\right) ^{-s} c_s \\&= \left( q^2-3q+3+H_q\left( \tfrac{1}{4},\tfrac{1}{2},\tfrac{3}{4};0,0,0\,|\,\psi ^{-4}\right) \right) \\&\quad + \left( 2 + qH_q\left( \tfrac{1}{4}, \tfrac{3}{4}; 0, \tfrac{1}{2} \,|\, \psi ^{-4}\right) \right) \\&\quad + \left( -1\right) ^{q^{\times }\,/4} q H_q\left( \tfrac{1}{2}; 0 \,|\, \psi ^{-4}\right) + \left( -1\right) ^{q^{\times }\,/4} - \left( \frac{g\left( \tfrac{q^{\times }}{4}\right) ^2 + g\left( \tfrac{3q^{\times }}{4}\right) ^2}{g\left( \tfrac{q^{\times }}{2}\right) }\right) \\&\quad + \left( -1\right) ^{q^{\times }\,/4} q H_q\left( \tfrac{1}{2}; 0 \,|\, \psi ^{-4}\right) + \left( -1\right) ^{q^{\times }\,/4} - \left( \frac{g\left( \tfrac{q^{\times }}{4}\right) ^2 + g\left( \tfrac{3q^{\times }}{4}\right) ^2}{g\left( \tfrac{q^{\times }}{2}\right) }\right) \\&= q^2-3q+3+H_q\left( \tfrac{1}{4},\tfrac{1}{2},\tfrac{3}{4};0,0,0\,|\,\psi ^{-4}\right) + qH_q\left( \tfrac{1}{4}, \tfrac{3}{4}; 0, \tfrac{1}{2} \,|\, \psi ^{-4}\right) )\\&\quad -2q H_q\left( \tfrac{1}{2}; 0 \,|\, \psi ^{-4}\right) - 2 \left( \frac{g\left( \tfrac{q^{\times }}{4}\right) ^2 + g\left( \tfrac{3q^{\times }}{4}\right) ^2}{g\left( \tfrac{q^{\times }}{2}\right) }\right) . \end{aligned} \end{aligned}$$

If $q\equiv 1 \pmod 8$, then by Lemmas 3.4.4, 3.4.7,  3.4.12, 3.4.21,  B.2.12, and B.2.17, we have:

B.2.9 $$\begin{aligned} \begin{aligned} \#U_{{\mathsf {F}}_2{\mathsf {L}}_2, \psi }\left( {\mathbb {F}}_q\right)&= \sum _{s \in S_1} \omega \left( a\right) ^{-s} c_s + \sum _{s \in S_4} \omega \left( a\right) ^{-s} c_s + \sum _{s \in S_5} \omega \left( a\right) ^{-s} c_s + \sum _{s \in S_6} \omega \left( a\right) ^{-s} c_s \\&\quad + \sum _{s \in S_{10}} \omega \left( a\right) ^{-s} c_s + \sum _{s \in S_{11}} \omega \left( a\right) ^{-s} c_s \\&= \left( q^2-3q+3+H_q\left( \tfrac{1}{4},\tfrac{1}{2},\tfrac{3}{4};0,0,0\,|\,\psi ^{-4}\right) \right) \\&\quad + \left( 2 + qH_q\left( \tfrac{1}{4}, \tfrac{3}{4}; 0, \tfrac{1}{2} \,|\, \psi ^{-4}\right) \right) \\&\quad + 2q H_q\left( \tfrac{1}{2}; 0 \,|\, \psi ^{-4}\right) + 2 - 2\left( \frac{g\left( \tfrac{q^{\times }}{4}\right) ^2 + g\left( \tfrac{3q^{\times }}{4}\right) ^2}{g\left( \tfrac{q^{\times }}{2}\right) }\right) \\&\quad + 2\omega \left( 2\right) ^{q^{\times }\,/4} qH_q\left( \tfrac{1}{8}, \tfrac{5}{8}; 0, \tfrac{1}{4} \,|\, \psi ^4\right) + 2\omega \left( 2\right) ^{q^{\times }\,/4}qH_q\left( \tfrac{3}{8}, \tfrac{7}{8}; 0, \tfrac{3}{4} \,|\, \psi ^4\right) \\&\quad - \tfrac{4}{q}g\left( \tfrac{q^{\times }}{8}\right) g\left( \tfrac{5q^{\times }}{8}\right) g\left( \tfrac{q^{\times }}{4}\right) - \tfrac{4}{q}g\left( \tfrac{3q^{\times }}{8}\right) g\left( \tfrac{7q^{\times }}{8}\right) g\left( \tfrac{3q^{\times }}{4}\right) \\&= q^2-3q+7+H_q\left( \tfrac{1}{4},\tfrac{1}{2},\tfrac{3}{4};0,0,0\,|\,\psi ^{-4}\right) +qH_q\left( \tfrac{1}{4}, \tfrac{3}{4}; 0, \tfrac{1}{2} \,|\, \psi ^{-4}\right) \\&\quad + 2q H_q\left( \tfrac{1}{2}; 0 \,|\, \psi ^{-4}\right) + 2\omega \left( 2\right) ^{q^{\times }\,/4} qH_q\left( \tfrac{1}{8}, \tfrac{5}{8}; 0, \tfrac{1}{4} \,|\, \psi ^4\right) \\&\quad + 2\omega \left( 2\right) ^{q^{\times }\,/4}qH_q\left( \tfrac{3}{8}, \tfrac{7}{8}; 0, \tfrac{3}{4} \,|\, \psi ^4\right) - 2\left( \frac{g\left( \tfrac{q^{\times }}{4}\right) ^2 + g\left( \tfrac{3q^{\times }}{4}\right) ^2}{g\left( \tfrac{q^{\times }}{2}\right) }\right) \\&\quad - \tfrac{4}{q}g\left( \tfrac{q^{\times }}{8}\right) g\left( \tfrac{5q^{\times }}{8}\right) g\left( \tfrac{q^{\times }}{4}\right) - \tfrac{4}{q}g\left( \tfrac{3q^{\times }}{8}\right) g\left( \tfrac{7q^{\times }}{8}\right) g\left( \tfrac{3q^{\times }}{4}\right) \end{aligned} \end{aligned}$$

as claimed. $$\square $$

Before proving the lemmas that associate the quantities $\sum _{s \in S_{10}}\omega (a)^{-s} c_s$ and $\sum _{s \in S_{11}}\omega (a)^{-s} c_s$ to hypergeometric sums, we need the following lemma.

##### Lemma B.2.10

Suppose $q \equiv 1 \pmod 8$ and $q = p^r$ for some natural number *r* and prime *p*. Then

$$\begin{aligned} \frac{g\left( \tfrac{3q^{\times }}{4}\right) g\left( \tfrac{q^{\times }}{8}\right) g\left( \tfrac{5q^{\times }}{8}\right) }{g\left( \tfrac{q^{\times }}{2}\right) } = \omega \left( 2\right) ^{q^{\times }\,/4} q. \end{aligned}$$

##### Proof

Since $q\equiv 1 \pmod 8$, we can use Hasse–Davenport with $N=2$ and $m = \tfrac{q^{\times }}{8}$ to get that

B.2.11 $$\begin{aligned} g\left( \tfrac{q^{\times }}{4}\right) = \omega \left( 2\right) ^{q^{\times }/4} \frac{g\left( \tfrac{q^{\times }}{8}\right) g\left( \tfrac{5q^{\times }}{8}\right) }{g\left( \tfrac{q^{\times }}{2}\right) }. \end{aligned}$$

By multiplying both sides by $g(\tfrac{3q^{\times }}{4})$, and dividing by $\omega (2)^{q^{\times }/4}$, we have

$$\begin{aligned} \omega \left( 2\right) ^{-q^{\times }/4}g\left( \tfrac{q^{\times }}{4}\right) g\left( \tfrac{3q^{\times }}{4}\right) =\frac{g\left( \tfrac{q^{\times }}{8}\right) g\left( \tfrac{5q^{\times }}{8}\right) g\left( \tfrac{3q^{\times }}{4}\right) }{g\left( \tfrac{q^{\times }}{2}\right) }. \end{aligned}$$

We obtain the identity above after noting that $g(\tfrac{q^{\times }}{4})g(\tfrac{3q^{\times }}{4}) = (-1)^{q^{\times }/4} q = q$, since $q\equiv 1 \pmod 8$ and that $\omega (2)^{q^{\times }\,/4} = \pm 1$ when $q\equiv 1\pmod 8$; hence, $\omega (2)^{q^{\times }\,/4} = \omega (2)^{-q^{\times }\,/4}$. $\square$

##### Lemma B.2.12

Suppose $q \equiv 1 \pmod 8$. Then for

$$\begin{aligned} S_{10} = \left\{ k(1,1,1,1,-4) + \tfrac{q^{\times }}{8}(0,2,1,5,0) : k \in {\mathbb {Z}}/q^{\times }{\mathbb {Z}}\right\} \end{aligned}$$

we have

$$\begin{aligned}&\sum _{s \in S_{10}} \omega \left( a\right) ^{-s} c_s = \omega \left( 2\right) ^{q^{\times }\,/4}qH_q\left( \tfrac{1}{8}, \tfrac{5}{8}; 0, \tfrac{1}{4} \,|\, \psi ^4\right) - \frac{1}{q}g\left( \tfrac{q^{\times }}{8}\right) g\left( \tfrac{5q^{\times }}{8}\right) g\left( \tfrac{q^{\times }}{4}\right) \\&\quad - \frac{1}{q}g\left( \tfrac{3q^{\times }}{8}\right) g\left( \tfrac{7q^{\times }}{8}\right) g\left( \tfrac{3q^{\times }}{4}\right) . \end{aligned}$$

##### Proof

First, we take the definition of the sum and take out all terms that are of the form $\ell \tfrac{q^{\times }}{4}$ to obtain the equality:

B.2.13 $$\begin{aligned} \begin{aligned} \sum _{s \in S_{10}} \omega \left( a\right) ^{-s} c_s&= \frac{1}{qq^{\times }} g\left( \tfrac{q^{\times }}{4}\right) g\left( \tfrac{q^{\times }}{8}\right) g\left( \tfrac{5q^{\times }}{8}\right) - \frac{1}{qq^{\times }} g\left( \tfrac{q^{\times }}{4}\right) g\left( \tfrac{q^{\times }}{2}\right) g\left( \tfrac{3q^{\times }}{8}\right) g\left( \tfrac{7q^{\times }}{8}\right) \\&\quad - \frac{1}{qq^{\times }} g\left( \tfrac{q^{\times }}{2}\right) g\left( \tfrac{3q^{\times }}{4}\right) g\left( \tfrac{q^{\times }}{8}\right) g\left( \tfrac{5q^{\times }}{8}\right) \\&\quad + \frac{1}{qq^{\times }} g\left( \tfrac{3q^{\times }}{4}\right) g\left( \tfrac{7q^{\times }}{8}\right) g\left( \tfrac{q^{\times }}{8}\right) \\&\quad + \frac{1}{qq^{\times }} \sum _{{\mathop {4k\not \equiv 0~({\text {mod}}~{q^{\times }})}\limits ^{k=0}}}^{q-2}\omega \left( 4\psi \right) ^{4k}g\left( k\right) \\&\quad \cdot g\left( k+\tfrac{q^{\times }}{4}\right) g\left( k + \tfrac{q^{\times }}{8}\right) g\left( k+\tfrac{5q^{\times }}{8}\right) g\left( -4k\right) . \end{aligned} \end{aligned}$$

Next, we use the Hasse–Davenport relationship to expand $g(-4k)$ and then use relation from 3.1.3(b) to cancel out the $g(k+\tfrac{q^{\times }}{4})$ factor in the summation. Through this, we obtain:

B.2.14 $$\begin{aligned} \begin{aligned} \sum _{s \in S_{10}} \omega \left( a\right) ^{-s} c_s&= \frac{1}{qq^{\times }} g\left( \tfrac{q^{\times }}{4}\right) g\left( \tfrac{q^{\times }}{8}\right) g\left( \tfrac{5q^{\times }}{8}\right) - \frac{1}{qq^{\times }} g\left( \tfrac{q^{\times }}{4}\right) g\left( \tfrac{q^{\times }}{2}\right) g\left( \tfrac{3q^{\times }}{8}\right) g\left( \tfrac{7q^{\times }}{8}\right) \\&\quad - \frac{1}{qq^{\times }} g\left( \tfrac{q^{\times }}{2}\right) g\left( \tfrac{3q^{\times }}{4}\right) g\left( \tfrac{q^{\times }}{8}\right) g\left( \tfrac{5q^{\times }}{8}\right) \\&\quad + \frac{1}{qq^{\times }} g\left( \tfrac{3q^{\times }}{4}\right) g\left( \tfrac{7q^{\times }}{8}\right) g\left( \tfrac{q^{\times }}{8}\right) \\&\quad + \frac{1}{q^{\times }} \sum _{{\mathop {4k\not \equiv 0~({\text {mod}}~{q^{\times }})}\limits ^{k=0}}}^{q-2} \omega \left( \psi \right) ^{4k} \\&\quad \cdot \frac{g\left( k+\tfrac{q^{\times }}{8}\right) g\left( k+\tfrac{5q^{\times }}{8}\right) g\left( -k+\tfrac{q^{\times }}{4}\right) g\left( -k+\tfrac{q^{\times }}{2}\right) }{g\left( \tfrac{q^{\times }}{2}\right) }. \end{aligned} \end{aligned}$$

Here, we reindex the summation by $m = k+ \tfrac{q^{\times }}{2}$ to obtain:

B.2.15 $$\begin{aligned} \begin{aligned} \sum _{s \in S_{10}} \omega \left( a\right) ^{-s} c_s&= \frac{1}{qq^{\times }} g\left( \tfrac{q^{\times }}{4}\right) g\left( \tfrac{q^{\times }}{8}\right) g\left( \tfrac{5q^{\times }}{8}\right) - \frac{1}{qq^{\times }} g\left( \tfrac{q^{\times }}{4}\right) g\left( \tfrac{q^{\times }}{2}\right) g\left( \tfrac{3q^{\times }}{8}\right) g\left( \tfrac{7q^{\times }}{8}\right) \\&\quad - \frac{1}{qq^{\times }} g\left( \tfrac{q^{\times }}{2}\right) g\left( \tfrac{3q^{\times }}{4}\right) g\left( \tfrac{q^{\times }}{8}\right) g\left( \tfrac{5q^{\times }}{8}\right) \\&\quad + \frac{1}{qq^{\times }} g\left( \tfrac{3q^{\times }}{4}\right) g\left( \tfrac{7q^{\times }}{8}\right) g\left( \tfrac{q^{\times }}{8}\right) \\&\quad + \frac{1}{q-1} \sum _{{\mathop {4m\not \equiv 0~({\text {mod}}~{q^{\times }})}\limits ^{m=0}}}^{q-2} \omega \left( \psi \right) ^{4m} \\&\quad \cdot \frac{g\left( m+\tfrac{q^{\times }}{8}\right) g\left( m+\tfrac{5q^{\times }}{8}\right) g\left( -m-\tfrac{q^{\times }}{4}\right) g\left( -m\right) }{g\left( \tfrac{q^{\times }}{2}\right) }. \end{aligned} \end{aligned}$$

We now multiply the final form by the expression

$$\begin{aligned} \frac{g\left( \tfrac{3q^{\times }}{4}\right) g\left( \tfrac{q^{\times }}{8}\right) g\left( \tfrac{5q^{\times }}{8}\right) }{g\left( \tfrac{3q^{\times }}{4}\right) g\left( \tfrac{q^{\times }}{8}\right) g\left( \tfrac{5q^{\times }}{8}\right) }=1. \end{aligned}$$

We put the denominator of this factor into the summation to relate the summation to a hypergeometric function but factor out the numerator along with a factor of $g(\tfrac{q^{\times }}{2})$. We then apply Lemma B.2.10 to this factor ahead of the summation. We thus obtain:

B.2.16 $$\begin{aligned} \sum _{s \in S_{10}} \omega \left( a\right) ^{-s} c_s&= \frac{1}{qq^{\times }} g\left( \tfrac{q^{\times }}{4}\right) g\left( \tfrac{q^{\times }}{8}\right) g\left( \tfrac{5q^{\times }}{8}\right) - \frac{1}{qq^{\times }} g\left( \tfrac{q^{\times }}{4}\right) g\left( \tfrac{q^{\times }}{2}\right) g\left( \tfrac{3q^{\times }}{8}\right) g\left( \tfrac{7q^{\times }}{8}\right) \nonumber \\&\quad - \frac{1}{qq^{\times }} g\left( \tfrac{q^{\times }}{2}\right) g\left( \tfrac{3q^{\times }}{4}\right) g\left( \tfrac{q^{\times }}{8}\right) g\left( \tfrac{5q^{\times }}{8}\right) \nonumber \\&\quad + \frac{1}{qq^{\times }} g\left( \tfrac{3q^{\times }}{4}\right) g\left( \tfrac{7q^{\times }}{8}\right) g\left( \tfrac{q^{\times }}{8}\right) \nonumber \\&\quad - \frac{ \omega \left( 2\right) ^{q^{\times }\,/4} q}{q^{\times }} \sum _{{\mathop {4m\not \equiv 0~({\text {mod}}~{q^{\times }})}\limits ^{m=0}}}^{q-2} \omega \left( \psi \right) ^{4m} \nonumber \\&\quad \cdot \frac{g\left( m+\tfrac{q^{\times }}{8}\right) g\left( m+\tfrac{5q^{\times }}{8}\right) g\left( -m-\tfrac{q^{\times }}{4}\right) g\left( -m\right) }{g\left( \tfrac{q^{\times }}{8}\right) g\left( \tfrac{5q^{\times }}{8}\right) g\left( -\tfrac{q^{\times }}{4}\right) g\left( 0\right) }. \end{aligned}$$

By comparing terms of the summations above and the hypergeometric function itself, we obtain the desired result. $\square$

##### Lemma B.2.17

Suppose $q \equiv 1 \pmod 8$. Then for

$$\begin{aligned} S_{11} = \left\{ k(1,1,1,1,-4) +\tfrac{q^{\times }}{8}(0,6,7,3,0) : k \in {\mathbb {Z}}/q^{\times }{\mathbb {Z}}\right\} \end{aligned}$$

we have

$$\begin{aligned}&\sum _{s \in S_{11}} \omega \left( a\right) ^{-s} c_s = \omega \left( 2\right) ^{q^{\times }\,/4} qH_q\left( \tfrac{3}{8}, \tfrac{7}{8}; 0, \tfrac{3}{4} \,|\, \psi ^4\right) \\&\quad - \frac{1}{q}g\left( \tfrac{q^{\times }}{8}\right) g\left( \tfrac{5q^{\times }}{8}\right) g\left( \tfrac{q^{\times }}{4}\right) - \frac{1}{q}g\left( \tfrac{3q^{\times }}{8}\right) g\left( \tfrac{7q^{\times }}{8}\right) g\left( \tfrac{3q^{\times }}{4}\right) . \end{aligned}$$

##### Proof

The proof is analogous to the proof in Lemma B.2.12 except we substitute $m = k + \tfrac{q^{\times }}{2}$. Alternatively, apply complex conjugation to the conclusion of Lemma  B.2.12, negating indices as in the proof of Lemma 3.5.6. $\square$

#### Step 3: Count points when at least one coordinate is zero

##### Lemma B.2.18

The following statements hold.

- If $$q\equiv 3 \pmod 4$$, then
  $$\begin{aligned} \#X_{{\mathsf {F}}_2{\mathsf {L}}_2, \psi }({\mathbb {F}}_q) - \#U_{{\mathsf {F}}_2{\mathsf {L}}_2, \psi }({\mathbb {F}}_q) = 2q. \end{aligned}$$
- If $$q\equiv 5 \pmod 8$$, then
  $$\begin{aligned} \#X_{{\mathsf {F}}_2{\mathsf {L}}_2, \psi }\left( {\mathbb {F}}_q\right) - \#U_{{\mathsf {F}}_2{\mathsf {L}}_2, \psi }\left( {\mathbb {F}}_q\right) = 2q-2 + 2\left( \frac{g\left( \tfrac{q^{\times }}{4}\right) ^2 + g\left( \tfrac{3q^{\times }}{4}\right) ^2}{g\left( \tfrac{q^{\times }}{2}\right) }\right) . \end{aligned}$$
- If $$q \equiv 1 \pmod 8$$, then
  $$\begin{aligned} \#X_{{\mathsf {F}}_2{\mathsf {L}}_2, \psi }\left( {\mathbb {F}}_q\right) - \#U_{{\mathsf {F}}_2{\mathsf {L}}_2, \psi }\left( {\mathbb {F}}_q\right)&= 10q - 6 + 2\left( \frac{g\left( \tfrac{q^{\times }}{4}\right) ^2 + g\left( \tfrac{3q^{\times }}{4}\right) ^2}{g\left( \tfrac{q^{\times }}{2}\right) }\right) \\&\quad + \tfrac{4}{q}g\left( \tfrac{q^{\times }}{4}\right) g\left( \tfrac{q^{\times }}{8}\right) g\left( \tfrac{5q^{\times }}{8}\right) \\&\quad + \tfrac{4}{q}g\left( \tfrac{3q^{\times }}{4}\right) g\left( \tfrac{3q^{\times }}{8}\right) g\left( \tfrac{7q^{\times }}{8}\right) . \end{aligned}$$

##### Proof

We do this case by case. If $x_1$ is the only variable equaling zero, then we must count the number of solutions in the open torus for the hypersurface $Z(x^4 + y^3z+ z^3y)\subset {\mathbb {P}}^2$. We can see by using Theorem 3.3.3 that this depends on *q*. Here, in case (a) we get $q-1$ points, in case (b) we get $q-3 + (g(\tfrac{q^{\times }}{4})^2 + g(\tfrac{3q^{\times }}{4})^2)g(\tfrac{q^{\times }}{2})^{-1}$, and in case (c) we get $q-3 + (g(\tfrac{q^{\times }}{4})^2 + g(\tfrac{3q^{\times }}{4})^2)g(\tfrac{q^{\times }}{2})^{-1} + 2q^{-1}(g(\tfrac{q^{\times }}{4})g(\tfrac{q^{\times }}{8})g(\tfrac{5q^{\times }}{8}) + g(\tfrac{3q^{\times }}{4})g(\tfrac{3q^{\times }}{8})g(\tfrac{7q^{\times }}{8}))$. There are two such cases, when either $x_1$ or $x_2$ is the only variable equaling 0.

Next is when both $x_1$ and $x_2$ are zero and the other two variables are nonzero. Here, the number of solutions is $1 + (-1)^{q^{\times }\,/2}$.

Next is when $x_3$ is zero but the rest are nonzero. Here, this is $(q-1)$ times the number of solutions of $Z(x^4+y^4)$ in the open torus of ${\mathbb {P}}^1$. We then get that the number of solutions is 0 if $q \not \equiv 1 \pmod 8$ and 4 if $q\equiv 1 \pmod 8$, hence $4q-4$ points. There are two such cases, when $x_3$ or $x_4$ are uniquely zero.

The next case is when $x_3$ and $x_4$ are both zero. Then the number of nonzero solutions is exactly the number of solutions of $Z(x^4+y^4)$ in the open torus of ${\mathbb {P}}^1$, i.e., 0 if $q \not \equiv 1 \pmod 8$ and 4 if $q\equiv 1 \pmod 8$.

There are no rational points where $x_1$ and $x_3$ are both zero and $x_2$ nonzero and the same when you swap $x_1$ with $x_2$ or $x_3$ with $x_4$. Finally, there are two more solutions: (0 : 0 : 1 : 0) and (0 : 0 : 0 : 1). We now count.

- If $q\not \equiv 1 \pmod 4$ and *q* is odd, then
  $$\begin{aligned} \#X_{{\mathsf {F}}_2{\mathsf {L}}_2, \psi }({\mathbb {F}}_q) - \#U_{{\mathsf {F}}_2{\mathsf {L}}_2, \psi }({\mathbb {F}}_q) = 2(q-1) + 0 + 0 + 0 + 2 = 2q. \end{aligned}$$
- If $$q\equiv 5 \pmod 8$$, then
  $$\begin{aligned} \#X_{{\mathsf {F}}_2{\mathsf {L}}_2, \psi }\left( {\mathbb {F}}_q\right) - \#U_{{\mathsf {F}}_2{\mathsf {L}}_2, \psi }\left( {\mathbb {F}}_q\right)&= 2\left( q-3 +\frac{g\left( \tfrac{q^{\times }}{4}\right) ^2 + g\left( \tfrac{3q^{\times }}{4}\right) ^2}{g\left( \tfrac{q^{\times }}{2}\right) }\right) \\&\quad + 2 + 0 + 0 + 2 \\&= 2q-2 + 2\left( \frac{g\left( \tfrac{q^{\times }}{4}\right) ^2 + g\left( \tfrac{3q^{\times }}{4}\right) ^2}{g\left( \tfrac{q^{\times }}{2}\right) }\right) . \end{aligned}$$
- If $$q\equiv 1 \pmod 8$$, then
  $$\begin{aligned}&\#X_{{\mathsf {F}}_2{\mathsf {L}}_2, \psi }\left( {\mathbb {F}}_q\right) - \#U_{{\mathsf {F}}_2{\mathsf {L}}_2, \psi }\left( {\mathbb {F}}_q\right) \\&\quad = 2\left( q-3 +\frac{g\left( \tfrac{q^{\times }}{4}\right) ^2 + g\left( \tfrac{3q^{\times }}{4}\right) ^2}{g\left( \tfrac{q^{\times }}{2}\right) } + \frac{2}{q}g\left( \tfrac{q^{\times }}{4}\right) g\left( \tfrac{q^{\times }}{8}\right) g\left( \tfrac{5q^{\times }}{8}\right) \right. \\&\qquad \left. + \frac{2}{q}g\left( \tfrac{3q^{\times }}{4}\right) g\left( \tfrac{3q^{\times }}{8}\right) g\left( \tfrac{7q^{\times }}{8}\right) \right) + 2 + 2\left( 4\left( q-1\right) \right) + 4 + 2 \\&= 10q - 6 + 2\frac{g\left( \tfrac{q^{\times }}{4}\right) ^2 + g\left( \tfrac{3q^{\times }}{4}\right) ^2}{g\left( \tfrac{q^{\times }}{2}\right) }+ \tfrac{4}{q}g\left( \tfrac{q^{\times }}{4}\right) g\left( \tfrac{q^{\times }}{8}\right) g\left( \tfrac{5q^{\times }}{8}\right) \\&\qquad + \tfrac{4}{q}g\left( \tfrac{3q^{\times }}{4}\right) g\left( \tfrac{3q^{\times }}{8}\right) g\left( \tfrac{7q^{\times }}{8}\right) . \end{aligned}$$

$$\square $$

#### Step 4: Combine Steps 2 and 3 to reach the conclusion

##### Proof of Proposition 3.6.1

We now combine Lemmas B.2.3 and B.2.18 as follows. For (a), for $q \equiv 3 \pmod {4}$,

$$\begin{aligned} \#X_{{\mathsf {F}}_2{\mathsf {L}}_2, \psi }\left( {\mathbb {F}}_q\right)&= q^2-3q+1+H_q\left( \tfrac{1}{4},\tfrac{1}{2},\tfrac{3}{4};0,0,0\,|\,\psi ^{-4}\right) - qH_q\left( \tfrac{1}{4}, \tfrac{3}{4}; 0, \tfrac{1}{2} \,|\, \psi ^{-4}\right) + 2q \\&= q^2-q+1+H_q\left( \tfrac{1}{4},\tfrac{1}{2},\tfrac{3}{4};0,0,0\,|\,\psi ^{-4}\right) - qH_q\left( \tfrac{1}{4}, \tfrac{3}{4}; 0, \tfrac{1}{2} \,|\, \psi ^{-4}\right) . \end{aligned}$$

For (b), for $$q\equiv 5 \pmod 8$$,

$$\begin{aligned} \#X_{{\mathsf {F}}_2{\mathsf {L}}_2, \psi }\left( {\mathbb {F}}_q\right)&=q^2-3q+3+H_q\left( \tfrac{1}{4},\tfrac{1}{2},\tfrac{3}{4};0,0,0\,|\,\psi ^{-4}\right) + qH_q\left( \tfrac{1}{4}, \tfrac{3}{4}; 0, \tfrac{1}{2} \,|\, \psi ^{-4}\right) \\&\quad -2q H_q\left( \tfrac{1}{2}; 0 \,|\, \psi ^{-4}\right) - 2 \left( \frac{g\left( \tfrac{q^{\times }}{4}\right) ^2 + g\left( \tfrac{3q^{\times }}{4}\right) ^2}{g\left( \tfrac{q^{\times }}{2}\right) }\right) \\&\quad + 2q-2 + 2\left( \frac{g\left( \tfrac{q^{\times }}{4}\right) ^2 + g\left( \tfrac{3q^{\times }}{4}\right) ^2}{g\left( \tfrac{q^{\times }}{2}\right) }\right) \\&= q^2-q+1+H_q\left( \tfrac{1}{4},\tfrac{1}{2},\tfrac{3}{4};0,0,0\,|\,\psi ^{-4}\right) \\&\quad + qH_q\left( \tfrac{1}{4}, \tfrac{3}{4}; 0, \tfrac{1}{2} \,|\, \psi ^{-4}\right) -2q H_q\left( \tfrac{1}{2}; 0 \,|\, \psi ^{-4}\right) \end{aligned}$$

Finally, for (c) with $q\equiv 1 \pmod 8$,

$$\begin{aligned}&\#X_{{\mathsf {F}}_2{\mathsf {L}}_2, \psi }\left( {\mathbb {F}}_q\right) = q^2-3q+5+H_q\left( \tfrac{1}{4},\tfrac{1}{2},\tfrac{3}{4};0,0,0\,|\,\psi ^{-4}\right) +qH_q\left( \tfrac{1}{4}, \tfrac{3}{4}; 0, \tfrac{1}{2} \,|\, \psi ^{-4}\right) \\&\qquad + 2q H_q\left( \tfrac{1}{2}; 0 \,|\, \psi ^{-4}\right) + 2\omega \left( 2\right) ^{q^{\times }\,/4}qH_q\left( \tfrac{1}{8}, \tfrac{5}{8}; 0, \tfrac{1}{4} \,|\, \psi ^4\right) \\&\qquad + 2\omega \left( 2\right) ^{q^{\times }\,/4}qH_q\left( \tfrac{3}{8}, \tfrac{7}{8}; 0, \tfrac{3}{4} \,|\, \psi ^4\right) \\&\qquad - 2\left( \frac{g\left( \tfrac{q^{\times }}{4}\right) ^2 + g\left( \tfrac{3q^{\times }}{4}\right) ^2}{g\left( \tfrac{q^{\times }}{2}\right) }\right) \\&\qquad - \tfrac{4}{q}g\left( \tfrac{q^{\times }}{8}\right) g\left( \tfrac{5q^{\times }}{8}\right) g\left( \tfrac{q^{\times }}{4}\right) - \tfrac{4}{q}g\left( \tfrac{3q^{\times }}{8}\right) g\left( \tfrac{7q^{\times }}{8}\right) g\left( \tfrac{3q^{\times }}{4}\right) \\&\qquad + 10q - 6 + 2\left( \frac{g\left( \tfrac{q^{\times }}{4}\right) ^2 + g\left( \tfrac{3q^{\times }}{4}\right) ^2}{g\left( \tfrac{q^{\times }}{2}\right) }\right) + \tfrac{4}{q}g\left( \tfrac{q^{\times }}{4}\right) g\left( \tfrac{q^{\times }}{8}\right) g\left( \tfrac{5q^{\times }}{8}\right) \\&\qquad + \tfrac{4}{q}g\left( \tfrac{3q^{\times }}{4}\right) g\left( \tfrac{3q^{\times }}{8}\right) g\left( \tfrac{7q^{\times }}{8}\right) \\&\quad = q^2+7q +1 +H_q\left( \tfrac{1}{4},\tfrac{1}{2},\tfrac{3}{4};0,0,0\,|\,\psi ^{-4}\right) +qH_q\left( \tfrac{1}{4}, \tfrac{3}{4}; 0, \tfrac{1}{2} \,|\, \psi ^{-4}\right) \\&\qquad + 2q H_q\left( \tfrac{1}{2}; 0 \,|\, \psi ^{-4}\right) + 2\omega \left( 2\right) ^{q^{\times }\,/4}qH_q\left( \tfrac{1}{8}, \tfrac{5}{8}; 0, \tfrac{1}{4} \,|\, \psi ^4\right) \\&\qquad +2\omega \left( 2\right) ^{q^{\times }\,/4}qH_q\left( \tfrac{3}{8}, \tfrac{7}{8}; 0, \tfrac{3}{4} \,|\, \psi ^4\right) . \end{aligned}$$

$$\square $$

### B.3. The pencil ${\mathsf {L}}_2{\mathsf {L}}_2$

#### Proposition B.3.1

The number of ${\mathbb {F}}_q$-points on ${\mathsf {L}}_2{\mathsf {L}}_2$ can be written in terms of hypergeometric functions, as follows:

- If $$q\equiv 3\pmod 4$$, then
  B.3.2 $$\begin{aligned} \#X_{{\mathsf {L}}_2{\mathsf {L}}_2,\psi }\left( {\mathbb {F}}_q\right) = q^2 + q+1 + H_q\left( \tfrac{1}{4}, \tfrac{1}{2}, \tfrac{3}{4}; 0,0,0 \,|\, \psi ^{-4}\right) - qH_q\left( \tfrac{1}{4}, \tfrac{3}{4}; 0, \tfrac{1}{2} \,|\, \psi ^{-4}\right) . \end{aligned}$$
- If $$q\equiv 1\pmod 4$$, then
  B.3.3 $$\begin{aligned} \begin{aligned} \#X_{{\mathsf {L}}_2{\mathsf {L}}_2,\psi }\left( {\mathbb {F}}_q\right)&= q^2+9q+1 + H_q\left( \tfrac{1}{4}, \tfrac{1}{2}, \tfrac{3}{4}; 0,0,0 \,|\, \psi ^{-4}\right) + qH_q\left( \tfrac{1}{4}, \tfrac{3}{4}; 0, \tfrac{1}{2} \,|\, \psi ^{-4}\right) \\&\quad + 2 \left( -1\right) ^{q^{\times }\,/4} \omega \left( \psi \right) ^{q^{\times }\,/2} q H_q\left( \tfrac{1}{8},\tfrac{3}{8},\tfrac{5}{8},\tfrac{7}{8};0,\tfrac{1}{4},\tfrac{1}{2},\tfrac{3}{4}\,|\,\psi ^{-4}\right) . \end{aligned} \end{aligned}$$

#### Remark B.3.4

Again, notice that the hypergeometric functions appearing in the point count correspond to exactly one of the Picard–Fuchs equations in Proposition A.2.2. We also see the appearance of eight additional trivial factors.

#### Step 1: Computing and clustering the characters

As with all the previous families, we first compute the set $S$ of solutions to the system of congruences given by Theorem 3.3.3:

- If $q\not \equiv 1\pmod 4$, and *q* is odd, then $S$ consists of
  (i) the set $S_1 = \{k(1,1,1,1,-4) : k \in {\mathbb {Z}}/q^{\times }{\mathbb {Z}}\}$ and
  (ii) the set $S_4 = \{k(1,1,1,1,-4) + \tfrac{q^{\times }}{2}(0,0,1,1,0) : k \in {\mathbb {Z}}/q^{\times }{\mathbb {Z}}\}.$
- If $$q\equiv 1 \pmod 4$$, then
  (i) the set $$S_1 = \{k(1,1,1,1,-4) : k \in {\mathbb {Z}}/q^{\times }{\mathbb {Z}}\},$$
  (ii) the set $S_4 = \{k(1,1,1,1,-4) + \tfrac{q^{\times }}{2}(0,0,1,1,0) : k \in {\mathbb {Z}}/q^{\times }{\mathbb {Z}}\},$ and
  (iii) two sets of the form $S_{12} = \{k(1,1,1,1,-4) + \tfrac{q^{\times }}{4}(0,2,3,1,2) : k \in {\mathbb {Z}}/q^{\times }{\mathbb {Z}}\}$.

#### Step 2: Counting points on the open subset with nonzero coordinates

##### Lemma B.3.5

Suppose $\psi \in {\mathbb {F}}_q^\times$. For *q*, we have:

- If $$q\equiv 3\pmod 4$$, then
  B.3.6 $$\begin{aligned} \#U_{{\mathsf {L}}_2{\mathsf {L}}_2, \psi }\left( {\mathbb {F}}_q\right) = q^2 -3q+1 + H_q\left( \tfrac{1}{4}, \tfrac{1}{2}, \tfrac{3}{4}; 0,0,0 \,|\, \psi ^{-4}\right) - qH_q\left( \tfrac{1}{4}, \tfrac{3}{4}; 0, \tfrac{1}{2} \,|\, \psi ^{-4}\right) . \end{aligned}$$
- If $$q\equiv 1\pmod 4$$, then
  B.3.7 $$\begin{aligned} \begin{aligned} \#U_{{\mathsf {L}}_2{\mathsf {L}}_2, \psi }\left( {\mathbb {F}}_q\right)&= q^2-3q+5 + H_q\left( \tfrac{1}{4}, \tfrac{1}{2}, \tfrac{3}{4}; 0,0,0 \,|\, \psi ^{-4}\right) + qH_q\left( \tfrac{1}{4}, \tfrac{3}{4}; 0, \tfrac{1}{2} \,|\, \psi ^{-4}\right) \\&\qquad + 2 \left( -1\right) ^{q^{\times }\,/4} \omega \left( \psi \right) ^{q^{\times }\,/2} q H_q\left( \tfrac{1}{8},\tfrac{3}{8},\tfrac{5}{8},\tfrac{7}{8};0,\tfrac{1}{4},\tfrac{1}{2},\tfrac{3}{4}\,|\,\psi ^{-4}\right) . \end{aligned} \end{aligned}$$

##### Proof

We do this by cases. For (a), where $q\equiv 3 \pmod 4$, then by using Theorem 3.3.3 with Lemmas 3.4.4 and 3.4.7 we have that

B.3.8 $$\begin{aligned} \begin{aligned} \#U_{{\mathsf {L}}_2{\mathsf {L}}_2, \psi }\left( {\mathbb {F}}_q\right)&= \sum _{s \in S_1} \omega \left( a\right) ^{-s}c_s + \sum _{s \in S_4} \omega \left( a\right) ^{-s}c_s \\&= q^2-3q+3 + H_q\left( \tfrac{1}{4}, \tfrac{1}{2}, \tfrac{3}{4}; 0,0,0 \,|\, \psi ^{-4}\right) -2 - qH_q\left( \tfrac{1}{4}, \tfrac{3}{4}; 0, \tfrac{1}{2} \,|\, \psi ^{-4}\right) \\&= q^2 -3q+1 + H_q\left( \tfrac{1}{4}, \tfrac{1}{2}, \tfrac{3}{4}; 0,0,0 \,|\, \psi ^{-4}\right) - qH_q\left( \tfrac{1}{4}, \tfrac{3}{4}; 0, \tfrac{1}{2} \,|\, \psi ^{-4}\right) . \end{aligned} \end{aligned}$$

For (b) with $q\equiv 1 \pmod 4$, by using Theorem 3.3.3 with Lemmas 3.4.4, 3.4.7, and B.3.10 below, we have that

B.3.9 $$\begin{aligned} \#U_{{\mathsf {L}}_2{\mathsf {L}}_2, \psi }\left( {\mathbb {F}}_q\right)&= \sum _{s \in S_1} \omega \left( a\right) ^{-s}c_s + \sum _{s \in S_4} \omega \left( a\right) ^{-s}c_s +2\sum _{s \in S_{12}} \omega \left( a\right) ^{-s}c_s \nonumber \\&= q^2-3q+3 + H_q\left( \tfrac{1}{4}, \tfrac{1}{2}, \tfrac{3}{4}; 0,0,0 \,|\, \psi ^{-4}\right) + 2 + qH_q\left( \tfrac{1}{4}, \tfrac{3}{4}; 0, \tfrac{1}{2} \,|\, \psi ^{-4}\right) \nonumber \\&\quad + 2 \left( -1\right) ^{q^{\times }\,/4} \omega \left( \psi \right) ^{q^{\times }\,/2} q H_q\left( \frac{1}{8},\frac{3}{8},\frac{5}{8},\frac{7}{8};0,\frac{1}{4},\frac{1}{2},\frac{3}{4}\,|\,\psi ^{-4}\right) \nonumber \\&= q^2-3q+5 + H_q\left( \tfrac{1}{4}, \tfrac{1}{2}, \tfrac{3}{4}; 0,0,0 \,|\, \psi ^{-4}\right) + qH_q\left( \tfrac{1}{4}, \tfrac{3}{4}; 0, \tfrac{1}{2} \,|\, \psi ^{-4}\right) \nonumber \\&\quad + 2 \left( -1\right) ^{q^{\times }\,/4} \omega \left( \psi \right) ^{q^{\times }\,/2} q H_q\left( \tfrac{1}{8},\tfrac{3}{8},\tfrac{5}{8},\tfrac{7}{8};0,\tfrac{1}{4},\tfrac{1}{2},\tfrac{3}{4}\,|\,\psi ^{-4}\right) . \end{aligned}$$

$$\square $$

##### Lemma B.3.10

Suppose that $q \equiv 1 \pmod 4$. Then

$$\begin{aligned} \sum _{s \in S_{12}} \omega \left( a\right) ^{-s} c_s = \left( -1\right) ^{q^{\times }\,/4} \omega \left( \psi \right) ^{q^{\times }\,/2} q H_q\left( \tfrac{1}{8},\tfrac{3}{8},\tfrac{5}{8},\tfrac{7}{8};0,\tfrac{1}{4},\tfrac{1}{2},\tfrac{3}{4}\,|\,\psi ^{-4}\right) . \end{aligned}$$

##### Proof

We start with the definition, factor out $\omega (\psi )^{q^{\times }\,/2}$, and then use the Hasse–Davenport relation (3.1.4) with $N=4$ with respect to $m=k$ to obtain:

B.3.11 $$\begin{aligned} \begin{aligned} \sum _{s \in S_{12}} \omega \left( a\right) ^{-s} c_s&= \frac{1}{qq^{\times }} \sum _{k=0}^{q-2} \omega \left( -4\psi \right) ^{4k + \tfrac{q^{\times }}{2}} g\left( k\right) g\left( k+ \tfrac{q^{\times }}{4}\right) g\left( k+\tfrac{q^{\times }}{2}\right) \\&\quad \cdot g\left( k+\tfrac{3q^{\times }}{4}\right) g\left( -4k+\tfrac{q^{\times }}{2}\right) \\&= \frac{\omega \left( \psi \right) ^{q^{\times }\,/2}}{qq^{\times }} \sum _{k=0}^{q-2} \omega \left( -4\psi \right) ^{4k} g\left( 4k\right) \omega \left( 4\right) ^{-4k} g\left( \tfrac{q^{\times }}{2}\right) \\&\quad \cdot g\left( \tfrac{q^{\times }}{4}\right) g\left( \tfrac{3q^{\times }}{4}\right) g\left( -4k+\tfrac{q^{\times }}{2}\right) . \end{aligned} \end{aligned}$$

Simplify with Lemma 3.1.3(b) to get

B.3.12 $$\begin{aligned} \sum _{s \in S_{12}} \omega \left( a\right) ^{-s} c_s = \frac{\left( -1\right) ^{q^{\times }\,/4}\omega \left( \psi \right) ^{q^{\times }\,/2}}{q-1} \sum _{k=0}^{q-2} \omega \left( -\psi \right) ^{4k} g\left( 4k\right) g\left( \tfrac{q^{\times }}{2}\right) g\left( -4k+\tfrac{q^{\times }}{2}\right) . \end{aligned}$$

Now, we use the Hasse–Davenport relation again with $N=2$ and $m=-4k$ to find:

B.3.13 $$\begin{aligned} \sum _{s \in S_{12}} \omega \left( a\right) ^{-s} c_s= & {} \frac{\left( -1\right) ^{q^{\times }\,/4}\omega \left( \psi \right) ^{q^{\times }\,/2}}{q-1} \sum _{k=0}^{q-2} \omega \left( -\psi \right) ^{4k} g\left( 4k\right) g\left( \tfrac{q^{\times }}{2}\right) \nonumber \\&\cdot \left( \frac{g\left( -8k\right) g\left( \tfrac{q^{\times }}{2}\right) \omega \left( 2\right) ^{8k}}{g\left( -4k\right) }\right) . \end{aligned}$$

We now simplify using Lemma 3.1.3(b) again and then expand the summation to get:

B.3.14 $$\begin{aligned} \begin{aligned} \sum _{s \in S_{12}} \omega (a)^{-s} c_s&= (-1)^{q^{\times }\,/4}\omega (\psi )^{q^{\times }\,/2}q \\&\quad \cdot \left( -\frac{4}{q^{\times }} + \frac{1}{q^{\times }} \sum _{{\mathop {4k \not \equiv 0 ~({\text {mod}}~{q^{\times }})}\limits ^{k=0}}}^{q-2} \omega (4\psi )^{4k} q^{-1}g(4k)^2g(-8k) \right) . \end{aligned} \end{aligned}$$

We finally reindex the sum with $m=-k$, yielding

B.3.15 $$\begin{aligned} \begin{aligned} \sum _{s \in S_{12}} \omega (a)^{-s} c_s&= (-1)^{q^{\times }\,/4}\omega (\psi )^{q^{\times }\,/2}\\&\quad \cdot q\left( -\frac{4}{q^{\times }} + \frac{1}{q^{\times }} \sum _{{\mathop {4m \not \equiv 0 ~({\text {mod}}~{q^{\times }})}\limits ^{m=0}}}^{q-2} \omega (4\psi )^{-4m} q^{-1}g(-4m)^2g(8m) \right) \\&= (-1)^{q^{\times }\,/4}\omega (\psi )^{q^{\times }\,/2}q H_q\left( \tfrac{1}{8},\tfrac{3}{8},\tfrac{5}{8},\tfrac{7}{8};0,\tfrac{1}{4},\tfrac{1}{2},\tfrac{3}{4}\,|\,\psi ^{-4}\right) \end{aligned} \end{aligned}$$

relating back to the finite-field hypergeometric sum. $\square$

#### Step 3: Count points when at least one coordinate is zero

##### Lemma B.3.16

Let *q* be an odd prime that is not 7. Then

- If $$q\equiv 3 \pmod 4$$, then
  $$\begin{aligned} \#X_{{\mathsf {L}}_2{\mathsf {L}}_2, \psi }({\mathbb {F}}_q) - \#U_{{\mathsf {L}}_2{\mathsf {L}}_2, \psi }({\mathbb {F}}_q) = 4q. \end{aligned}$$
- If $$q\equiv 1 \pmod 4$$, then
  $$\begin{aligned} \#X_{{\mathsf {L}}_2{\mathsf {L}}_2, \psi }({\mathbb {F}}_q) - \#U_{{\mathsf {L}}_2{\mathsf {L}}_2, \psi }({\mathbb {F}}_q) = 12q - 4. \end{aligned}$$

##### Proof

Suppose that $x_1=0$ and the rest are nonzero. Then, by using Theorem 3.3.3, we can see that there are $(q-1)((-1)^{q^{\times }\,/2} +1)$ such points. Since there are four choices of one coordinate being zero, this counts $4(q-1)((-1)^{q^{\times }\,/2} +1)$ points.

Suppose now that $x_1=x_2=0$ and the rest nonzero, then by Theorem 3.3.3 again, we have $((-1)^{q^{\times }\,/2} +1)$ points. By symmetry, this is the same as the case where $x_3=x_4=0$ and the rest nonzero, so we now count $2((-1)^{q^{\times }\,/2} +1)$.

Next, suppose $x_1=x_3=0$ and the rest nonzero. Automatically, the polynomial vanishes, hence there are $q-1$ such points. There are 4 such cases from choosing one of $x_1$ and $x_2$ and another from $x_3$ and $x_4$ to equal zero, hence we count $4(q-1)$ points. Finally, the four points where three coordinates are zero are all solutions, hence we count 4 more points. Thus,

$$\begin{aligned} \#X_{{\mathsf {L}}_2{\mathsf {L}}_2, \psi }\left( {\mathbb {F}}_q\right) - \#U_{{\mathsf {L}}_2{\mathsf {L}}_2, \psi }\left( {\mathbb {F}}_q\right)= & {} 4\left( q-1\right) \left( \left( -1\right) ^{q^{\times }\,/2} +1\right) + 2\left( \left( -1\right) ^{q^{\times }\,/2} +1\right) \\&+ 4\left( q-1\right) + 4. \end{aligned}$$

If $q\equiv 3 \pmod 4$, then $(-1)^{q^{\times }\,/2} = -1$, so $\#X_{{\mathsf {L}}_2{\mathsf {L}}_2, \psi }({\mathbb {F}}_q) - \#U_{{\mathsf {L}}_2{\mathsf {L}}_2, \psi }({\mathbb {F}}_q) = 4q$. If $q\equiv 1 \pmod 4$, then $(-1)^{q^{\times }\,/2} = 1$, so $\#X_{{\mathsf {L}}_2{\mathsf {L}}_2, \psi }({\mathbb {F}}_q) - \#U_{{\mathsf {L}}_2{\mathsf {L}}_2, \psi }({\mathbb {F}}_q) = 12q-4$. $\square$

#### Step 4: Combine Steps 2 and 3 to reach the conclusion

##### Proof of Proposition 3.6.2

If $q \not \equiv 1 \pmod 4$, then by Lemmas B.3.5 and B.3.16, we have that

B.3.17 $$\begin{aligned} \#X_{{\mathsf {L}}_2{\mathsf {L}}_2,\psi }\left( {\mathbb {F}}_q\right)= & {} \left( q^2 -3q+1 + H_q\left( \tfrac{1}{4}, \tfrac{1}{2}, \tfrac{3}{4}; 0,0,0 \,|\, \psi ^{-4}\right) - qH_q\left( \tfrac{1}{4}, \tfrac{3}{4}; 0, \tfrac{1}{2} \,|\, \psi ^{-4}\right) \right) \nonumber \\&+ 4q= q^2 + q+1 + H_q\left( \tfrac{1}{4}, \tfrac{1}{2}, \tfrac{3}{4}; 0,0,0 \,|\, \psi ^{-4}\right) \nonumber \\&- qH_q\left( \tfrac{1}{4}, \tfrac{3}{4}; 0, \tfrac{1}{2} \,|\, \psi ^{-4}\right) . \end{aligned}$$

If $q \equiv 1 \pmod 4$, then by Lemmas B.3.5 and B.3.16, we have that

B.3.18 $$\begin{aligned} \#X_{{\mathsf {L}}_2{\mathsf {L}}_2,\psi }\left( {\mathbb {F}}_q\right)&= q^2-3q+5 + H_q\left( \tfrac{1}{4}, \tfrac{1}{2}, \tfrac{3}{4}; 0,0,0 \,|\, \psi ^{-4}\right) + qH_q\left( \tfrac{1}{4}, \tfrac{3}{4}; 0, \tfrac{1}{2} \,|\, \psi ^{-4}\right) \nonumber \\&\quad + 2 \left( -1\right) ^{q^{\times }\,/4} \omega \left( \psi \right) ^{q^{\times }\,/2} q H_q\left( \tfrac{1}{8},\tfrac{3}{8},\tfrac{5}{8},\tfrac{7}{8};0,\tfrac{1}{4},\tfrac{1}{2},\tfrac{3}{4}\,|\,\psi ^{-4}\right) + 12q-4 \nonumber \\&= q^2+9q+1 + H_q\left( \tfrac{1}{4}, \tfrac{1}{2}, \tfrac{3}{4}; 0,0,0 \,|\, \psi ^{-4}\right) + qH_q\left( \tfrac{1}{4}, \tfrac{3}{4}; 0, \tfrac{1}{2} \,|\, \psi ^{-4}\right) \nonumber \\&\quad + 2 \left( -1\right) ^{q^{\times }\,/4} \omega \left( \psi \right) ^{q^{\times }\,/2} q H_q\left( \tfrac{1}{8},\tfrac{3}{8},\tfrac{5}{8},\tfrac{7}{8};0,\tfrac{1}{4},\tfrac{1}{2},\tfrac{3}{4}\,|\,\psi ^{-4}\right) . \end{aligned}$$

$$\square $$

### B.2. The pencil ${\mathsf {L}}_4$

#### Proposition B.4.1

The number of ${\mathbb {F}}_q$ points on ${\mathsf {L}}_4$ for *q* odd is given in terms of hypergeometric functions as follows.

- If $$q\not \equiv 1\pmod 5$$, then
  $$\begin{aligned} \#X_{{\mathsf {L}}_4,\psi }({\mathbb {F}}_q)=q^2+3q+1+H_q\left( \tfrac{1}{4},\tfrac{1}{2},\tfrac{3}{4};0,0,0\,|\,\psi ^{-4}\right) . \end{aligned}$$
- If $$q\equiv 1 \pmod 5$$, then
  $$\begin{aligned} \#X_{{\mathsf {L}}_4,\psi }({\mathbb {F}}_q)= & {} q^2+3q+1+H_q\left( \tfrac{1}{4},\tfrac{1}{2},\tfrac{3}{4};0,0,0\,|\,\psi ^{-4}\right) \\&+4qH_q\left( \tfrac{1}{5},\tfrac{2}{5}, \tfrac{3}{5},\tfrac{4}{5} ;0, \tfrac{1}{4},\tfrac{1}{2}, \tfrac{3}{4}\,|\,\psi ^{4}\right) . \end{aligned}$$

#### Remark B.4.2

As before, we can identify the parameters of the hypergeometric function

$$\begin{aligned} H_q\left( \tfrac{1}{5},\tfrac{2}{5}, \tfrac{3}{5},\tfrac{4}{5} ;0, \tfrac{1}{4},\tfrac{1}{2}, \tfrac{3}{4}\,|\,\psi ^{4}\right) \end{aligned}$$

with the parameters of the second Picard–Fuchs equation in Proposition A.3.2. If we use Theorem 3.4 of [3] again to shift parameters, then we see that in fact all of the Picard–Fuchs equations satisfied by the non-holomorphic periods correspond to this same hypergeometric motive over ${\mathbb {Q}}$.

Also notice that in the discussion following Proposition A.3.2, we see two periods that are “missed” by the Griffiths–Dwork method, and here they clearly correspond to the two additional trivial factors coming from the 3*q* term in the point count.

#### Step 1: Computing and clustering the characters

Again, we compute the solutions to the system of congruences given by Theorem 3.3.3. We obtain

- If $q\not \equiv 1\pmod 5$, the solution set is
  (i) the set $$S_1 = \{ k(1,1,1,1,-4) : k \in {\mathbb {Z}}/ q^{\times }{\mathbb {Z}}\}$$.
- If $q\equiv 1\pmod 5$, then clusters of solutions are
  (i) the set $S_1 = \{ k(1,1,1,1,-4) : k \in {\mathbb {Z}}/ q^{\times }{\mathbb {Z}}\}$ and
  (ii) four sets of the form $S_{13} = \{ k(1,1,1,1,-4) + \frac{q^{\times }}{5}(1,2,4,3,0) : k \in {\mathbb {Z}}/ q^{\times }{\mathbb {Z}}\}$.

#### Step 2: Counting points on the open subset with nonzero coordinates

##### Lemma B.4.3

Suppose $\psi \in {\mathbb {F}}_q^\times$. For *q* odd, we have:

- If $$q\not \equiv 1\pmod 5$$, then
  B.4.4 $$\begin{aligned} \#U_{{\mathsf {L}}_4, \psi }({\mathbb {F}}_q) = q^2-3q+3 + H_q\left( \tfrac{1}{4}, \tfrac{1}{2}, \tfrac{3}{4}; 0,0,0 \,|\, \psi ^{-4}\right) . \end{aligned}$$
- If $$q\equiv 1\pmod 5$$, then
  B.4.5 $$\begin{aligned} \#U_{{\mathsf {L}}_4, \psi }\left( {\mathbb {F}}_q\right) = q^2-3q+3 + H_q\left( \tfrac{1}{4}, \tfrac{1}{2}, \tfrac{3}{4}; 0,0,0 \,|\, \psi ^{-4}\right) + 4q H_q\left( \tfrac{1}{5},\tfrac{2}{5}, \tfrac{3}{5},\tfrac{4}{5} ;0, \tfrac{1}{4},\tfrac{1}{2}, \tfrac{3}{4}\,|\,\psi ^{4}\right) . \end{aligned}$$

##### Proof

When $q \not \equiv 1 \pmod 5$, we know that there is only one cluster of characters, $S_1$. By Lemma 3.4.4, we know that

B.4.6 $$\begin{aligned} \#U_{{\mathsf {L}}_4, \psi }({\mathbb {F}}_q) = \sum _{s \in S_1} \omega (a)^{-s} c_s= q^2-3q+3 + H_q\left( \tfrac{1}{4}, \tfrac{1}{2}, \tfrac{3}{4}; 0,0,0 \,|\, \psi ^{-4}\right) . \end{aligned}$$

When $q\equiv 1 \pmod 5$, we have two types of clusters of characters. By Lemmas 3.4.4 and B.4.8,

B.4.7 $$\begin{aligned} \#U_{{\mathsf {L}}_4, \psi }\left( {\mathbb {F}}_q\right)&= \sum _{s \in S_1} \omega \left( a\right) ^{-s} c_s + 4 \sum _{s \in S_{13}} \omega \left( a\right) ^{-s} c_s \nonumber \\&= q^2-3q+3 + H_q\left( \tfrac{1}{4}, \tfrac{1}{2}, \tfrac{3}{4}; 0,0,0 \,|\, \psi ^{-4}\right) \nonumber \\&\quad + 4q H_q\left( \tfrac{1}{5},\tfrac{2}{5}, \tfrac{3}{5},\tfrac{4}{5} ;0, \tfrac{1}{4},\tfrac{1}{2}, \tfrac{3}{4}\,|\,\psi ^{4}\right) . \end{aligned}$$

$$\square $$

We now just need a hypergeometric way to write the point count associated to the cluster $S_{13}$.

##### Lemma B.4.8

If $q \equiv 1 \pmod 5$ and *q* is odd, then

B.4.9 $$\begin{aligned} \sum _{s\in S_{13}} \omega (a)^{-s} c_s = qH_q\left( \tfrac{1}{5},\tfrac{2}{5}, \tfrac{3}{5},\tfrac{4}{5} ;0, \tfrac{1}{4},\tfrac{1}{2}, \tfrac{3}{4}\,|\,\psi ^{4}\right) . \end{aligned}$$

##### Proof

By using the hybrid hypergeometric definition, this equality is found quickly:

B.4.10 $$\begin{aligned} \sum _{s\in S_{13}} \omega \left( a\right) ^{-s} c_s&= \frac{1}{qq^{\times }} \sum _{k=0}^{q-2} \omega \left( -4^4\psi ^4\right) g\left( k+\tfrac{q^{\times }}{5}\right) g\left( k+\tfrac{2q^{\times }}{5}\right) \nonumber \\&\quad \cdot g\left( k+\tfrac{3q^{\times }}{5}\right) g\left( k + \tfrac{4q^{\times }}{5}\right) g\left( -4k\right) \nonumber \\&= \frac{1}{qq^{\times }} \sum _{k=0}^{q-2} \omega \left( 4^4\psi ^4\right) g\left( k+\tfrac{q^{\times }}{5}\right) g\left( k+\tfrac{2q^{\times }}{5}\right) \nonumber \\&\quad \cdot g\left( k+\tfrac{3q^{\times }}{5}\right) g\left( k + \tfrac{4q^{\times }}{5}\right) g\left( -4k\right) \nonumber \\&= \frac{q}{q^{\times }} \sum _{k=0}^{q-2} \omega \left( 4^4\psi ^4\right) \nonumber \\&\quad \cdot \frac{g\left( k+\tfrac{q^{\times }}{5}\right) g\left( k+\tfrac{2q^{\times }}{5}\right) g\left( k+\tfrac{3q^{\times }}{5}\right) g\left( k + \tfrac{4q^{\times }}{5}\right) }{q^2}g\left( -4k\right) \nonumber \\&= \frac{q}{q^{\times }} \sum _{k=0}^{q-2} \omega \left( 4^4\psi ^4\right) \nonumber \\&\quad \cdot \frac{g\left( k+\tfrac{q^{\times }}{5}\right) g\left( k+\tfrac{2q^{\times }}{5}\right) g\left( k+\tfrac{3q^{\times }}{5}\right) g\left( k + \tfrac{4q^{\times }}{5}\right) }{g\left( \tfrac{q^{\times }}{5}\right) g\left( \tfrac{2q^{\times }}{5}\right) g\left( \tfrac{3q^{\times }}{5}\right) g\left( \tfrac{4q^{\times }}{5}\right) } g\left( -4k\right) \nonumber \\&= qH_q\left( \tfrac{1}{5},\tfrac{2}{5}, \tfrac{3}{5},\tfrac{4}{5} ;0, \tfrac{1}{4},\tfrac{1}{2}, \tfrac{3}{4}\,|\,\psi ^{4}\right) . \end{aligned}$$

The last line uses the hybrid definition (Definition 3.2.7) of the hypergeometric function

$$\begin{aligned} H_q\left( \tfrac{1}{5},\tfrac{2}{5}, \tfrac{3}{5},\tfrac{4}{5} ;0, \tfrac{1}{4},\tfrac{1}{2}, \tfrac{3}{4}\,|\,\psi ^{4}\right) . \end{aligned}$$

Even though the hypergeometric function is defined over ${\mathbb {Q}}$, we can get to the relation much more quickly using the hybrid definition.

#### Step 3: Count points when at least one coordinate is zero.

##### Lemma B.4.11

If *q* is odd and not 7, then

$$\begin{aligned} \#X_{{\mathsf {L}}_4,\psi }({\mathbb {F}}_q) - \#U_{{\mathsf {L}}_4,\psi }({\mathbb {F}}_q) = 6q-2. \end{aligned}$$

##### Proof

First, we count the number of rational points when exactly variable equals zero. Without loss of generality, assume $x_1=0$. Then we want solutions of

$$\begin{aligned} x_2^3 x_3 + x_3^3 x_4 = 0 \end{aligned}$$

which we can solve for $x_4$. Since $x_4$ is completely determined by $x_2$ and $x_3$, we can normalize $x_2=1$ and see there are exactly $q-1$ solutions when only $x_1$ is zero. By symmetry, this shows that there are $4q-4$ solutions when exactly one variable equals zero. If two consecutive variables are zero (say $x_1=x_2=0$), then we want solutions of the form $x_3^3x_4 = 0$ which implies that a third variable equals zero. Thus, there are 4 solutions with 3 variables equaling zero and no solutions when exactly two variables equal zero and those variables are consecutive. Lastly, if two non-consecutive variables are zero then any other solution works. For any pair of non-consecutive variables (of which there are two), we then have $q-1$ solutions. Therefore,

$$\begin{aligned} \#X_{{\mathsf {L}}_4,\psi }({\mathbb {F}}_q) - \#U_{{\mathsf {L}}_4,\psi }({\mathbb {F}}_q) = 4q-4 + 4 + 2(q-1) = 6q-2. \end{aligned}$$

$$\square $$

#### Step 4: Combine Steps 2 and 3 to find conclusion

We now prove Proposition 3.6.3.

##### Proof of Proposition 3.6.3

Combining Lemmas B.4.3 and B.4.11, we have

- If $$q\not \equiv 1\pmod 5$$, then
  B.4.12 $$\begin{aligned} \#X_{{\mathsf {L}}_4, \psi }\left( {\mathbb {F}}_q\right)= & {} \left( q^2-3q+3 + H_q\left( \tfrac{1}{4}, \tfrac{1}{2}, \tfrac{3}{4}; 0,0,0 \,|\, \psi ^{-4}\right) \right) + \left( 6q-2\right) \nonumber \\= & {} q^2+3q+1 + H_q\left( \tfrac{1}{4}, \tfrac{1}{2}, \tfrac{3}{4}; 0,0,0 \,|\, \psi ^{-4}\right) . \end{aligned}$$
- If $$q\equiv 1\pmod 5$$, then
  B.4.13 $$\begin{aligned} \#X_{{\mathsf {L}}_4, \psi }\left( {\mathbb {F}}_q\right)= & {} \left( q^2-3q+3 + H_q\left( \tfrac{1}{4}, \tfrac{1}{2}, \tfrac{3}{4}; 0,0,0 \,|\, \psi ^{-4}\right) \right. \nonumber \\&\left. + 4q H_q\left( \tfrac{1}{5},\tfrac{2}{5}, \tfrac{3}{5},\tfrac{4}{5} ;0, \tfrac{1}{4},\tfrac{1}{2}, \tfrac{3}{4}\,|\,\psi ^{4}\right) \right) + \left( 6q-2\right) \nonumber \\= & {} q^2+3q+1 + H_q\left( \tfrac{1}{4}, \tfrac{1}{2}, \tfrac{3}{4}; 0,0,0 \,|\, \psi ^{-4}\right) \nonumber \\&+ 4q H_q\left( \tfrac{1}{5},\tfrac{2}{5}, \tfrac{3}{5},\tfrac{4}{5} ;0, \tfrac{1}{4},\tfrac{1}{2}, \tfrac{3}{4}\,|\,\psi ^{4}\right) . \end{aligned}$$

This completes the proof. $\square$
